# Evolution of an amphibian-specific family of epidermal differentiation-associated genes

**DOI:** 10.1093/molbev/msag196

**Published:** 2026-08-06

**Authors:** Attila Placido Sachslehner, Julia Steinbinder, Anja Wagner, Klaus Kratochwill, Leopold Eckhart

**Affiliations:** Department of Dermatology, Medical University of Vienna, Waehringer Guertel 18-20, Vienna 1090, Austria; Department of Evolutionary Biology, University of Vienna, Vienna, Austria; Department of Dermatology, Medical University of Vienna, Waehringer Guertel 18-20, Vienna 1090, Austria; Core Facility Proteomics, Medical University of Vienna, Vienna, Austria; Core Facility Proteomics, Medical University of Vienna, Vienna, Austria; Division of Pediatric Nephrology and Gastroenterology, Department of Pediatrics and Adolescent Medicine, Comprehensive Center for Pediatrics, Medical University of Vienna, Vienna, Austria; Department of Dermatology, Medical University of Vienna, Waehringer Guertel 18-20, Vienna 1090, Austria

**Keywords:** protein evolution, gene family, sequence complexity, skin, cornification

## Abstract

The surface layer of the skin and hard skin appendages of land-dwelling vertebrates depend on epithelial differentiation-associated proteins, which increase the mechanical resilience of cells upon cornification. These proteins, exemplified by keratin-associated proteins (KRTAPs) in mammals and epidermal differentiation complex (EDC) proteins in all tetrapods, have low amino acid sequence complexity and interact with other proteins to form stable supramolecular structures. No KRTAPs and only few EDC proteins exist in amphibians, suggesting an alternative mechanism of epithelial cornification. Here, we used comparative genomics and proteomics to identify a previously uncharacterized group of epidermal differentiation-associated proteins that are specific for amphibians. These proteins are encoded by genes of the amphibian epidermal differentiation cluster (AEDC), which has evolved by tandem duplications and sequence modifications of an ancestral gene at a locus syntenic with that of *adipogenesis regulatory factor* in other vertebrates. AEDC genes are specifically expressed in the epithelium of the skin and its appendages, such as the cornified spines within nuptial pads of frogs. Internal sequence duplications within AEDC proteins have led to amino acid sequence repeats similar to those of EDC proteins and KRTAPs. We conclude that epidermal differentiation-associated proteins have originated multiple times and evolved similar sequence features in amphibians and other clades of vertebrates.

## Introduction

Mechanically and chemically resilient surface structures are formed by various mechanisms in different clades of animals (Akat et al. 2022; Cohen et al. 2025; Bai et al. 2026). One mechanism is the extracellular deposition of biomaterials, such as chitin and proteins in the exoskeleton of arthropods (Roer et al. 2015; Qi and Liu 2025), calcium carbonate and proteins in the shell of snails (Clark et al. 2020), and calcium phosphate (apatite) and proteins in the enamel of vertebrate teeth (Pandya and Diekwisch 2021). Another mechanism is based on the intracellular remodeling of epithelia, commonly known as cornification. The outermost epidermal layer of tetrapods and hard skin appendages, such as hair, feathers and claws of tetrapods, breeding tubercles of fishes, and horny teeth of cyclostomes, are built by cornified epithelial cells (Alibardi 2003; Akat et al. 2022; Holthaus et al. 2025; Sachslehner and Eckhart 2025).

Cornification converts metabolically active cells into inactive but highly resilient cell ghosts that remain stably interconnected (Candi et al. 2005; Matsui and Amagai 2015). Mechanistically, this remodeling is driven by the intracellular accumulation, binding and cross-linking of specific proteins, followed by the breakdown of organelles. The quantitatively dominant proteins of epithelial cells are keratins, which form intermediate filaments to build the cytoskeleton (Feng et al. 2013). Specific keratins are expressed prior to cornification of epidermal keratinocytes (Fischer et al. 2014) and hair follicle epithelial cells (trichocytes) (Eckhart and Ehrlich 2018). However, keratin filaments alone do not suffice as structural components of cornified cells. Additional proteins, here referred to as cornification proteins, are produced during terminal differentiation to form a proteinaceous envelope around the cytoplasm and a matrix between keratin filaments (Kypriotou et al. 2012; Fraser and Parry 2018). Examples of cornification proteins are loricrin and its orthologs in amniotes (Hohl et al. 1991; Strasser et al. 2014), keratin-associated proteins (KRTAPs) in mammals (Bornschlögl et al. 2016; Plowman 2018), and KRTAP-like (KRTAPL) proteins in cyclostomes (Sachslehner et al. 2025). Although the sequence features critical for the function of these proteins are not fully understood, cornification proteins have in common a lack of a conventional protein folding domain, low sequence complexity, the presence of sequence repeats, and a biased amino acid composition, suggesting that they largely consist of intrinsically disordered regions (Shamilov et al. 2021). Many but not all cornification proteins contain elevated numbers of amino acid residues amenable to covalent cross-linking, such as transglutamination at glutamine (Q) and lysine (K) residues and disulfide bond formation at cysteine residues (Holthaus et al. 2025).

Cornification proteins are encoded by genes arranged in clusters, indicating that they have evolved by gene duplications and diversifying mutations. The epidermal differentiation complex (EDC) is a cluster of genes located between S100A genes in tetrapods (Kypriotou et al. 2012; Strasser et al. 2014). Comparative genomics and molecular phylogenetics have revealed that EDC genes have evolved from an S100A gene and subsequently diverged into S100 fused-type proteins (SFTPs), such as filaggrin (Smith et al. 2006), and single-coding exon EDC (SEDC) genes, such as loricrin and corneous beta proteins (CBPs), with the latter being confined to sauropsids (Strasser et al. 2014; Lin et al. 2021; Parry 2021). KRTAP genes form a cluster flanked by type I hair keratins, and they are supposed to have evolved from the first exon of a keratin gene (Rogers et al. 2004; Eckhart and Ehrlich 2018; Wu and Irwin 2018). KRTAPL genes of cyclostomes (lampreys and hagfish) are also arranged in clusters, but the gene from which they have evolved is not known (Sachslehner et al. 2025). In line with their independent evolutionary origins, the exon–intron structures differ between cornification protein genes. In EDC genes, one (SEDC) or two (SFTP) protein-coding exons are preceded by a noncoding exon (Strasser et al. 2014). KRTAP genes consist of a single exon containing the entire coding sequence (Eckhart and Ehrlich 2018) , and KRTAPL genes of cyclostomes consist of two exons containing the coding sequence (Sachslehner et al. 2025).

**Figure 1:**
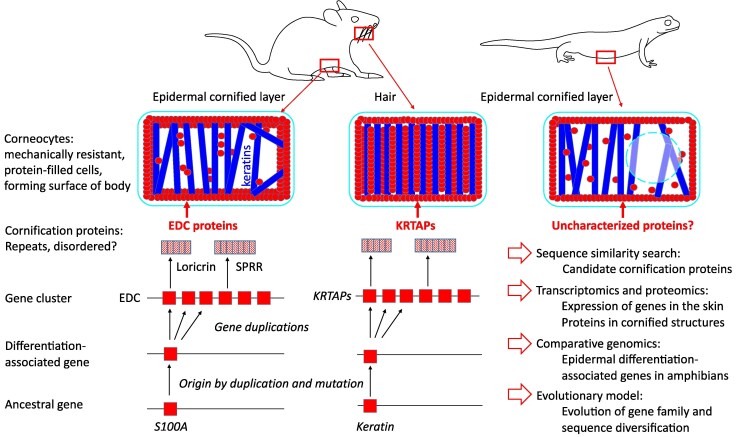
Schematic depiction of protein-dependent cornification in mammals and the approach for the identification of epithelial differentiation-associated proteins in amphibians. The evolution and function of mammalian cornification proteins are schematically depicted. The localization of cornification proteins (red circles) relative to keratin intermediate filaments (blue bars) within cornified cells (corneocytes) is schematically depicted. As only a small number of EDC protein homologs exist in amphibians (Mlitz et al. 2017; Sachslehner and Eckhart 2022), we tested the hypothesis that the differentiation of amphibian skin epithelia is associated with other proteins regarded as candidate cornification proteins.

Amphibians have a cornified cell layer on the skin surface, and subclades of amphibians develop cornified skin appendages, such as claws, spines, and horny teeth (Alibardi 2003; Maddin et al 2009; Holthaus et al. 2025). In contrast to epithelial keratins, which exist in great diversity including some specifically expressed in hard skin appendages (Suzuki et al. 2017; Carron et al. 2024), cornification proteins have been identified in only a few amphibians. One or two EDC genes of the SFTP-type are present in all amphibians investigated so far (Mlitz et al. 2017; Sachslehner and Eckhart 2022), and two to six additional EDC genes of the SEDC-type exist in caecilians (*Gymnophiona*) but not in frogs and toads (*Anura*) and salamanders (*Caudata*) (Sachslehner and Eckhart 2022). The low number of cornification proteins of amphibians (*n* = 1–8 EDC proteins) relative to amniotes (*n* > 50 EDC, CBP, or KRTAPs) (Holthaus et al. 2025) appears to correlate with the extent of skin cornification. Amphibians have a thin cornified layer of the epidermis and small, hard cornified appendages, such as claws in *Xenopus* frogs (Maddin et al. 2009), whereas amniotes develop an epidermis with multiple layers of cornified keratinocytes and large cornified skin appendages, such as claws, scales, feathers, and hair. However, it is also conceivable that the full range of cornification proteins of amphibians has not been discovered yet, and their epidermal cornification requires a larger diversity of structural proteins than currently assumed (Fig. 1).

We hypothesized that the scarcity of EDC protein homologs in amphibians is compensated by as yet uncharacterized proteins. Here, we identify epidermal differentiation-associated proteins that have evolved in amphibians and share important traits with canonical EDC proteins.

## Results

### Identification of candidate cornification proteins in amphibians

**Figure 2:**
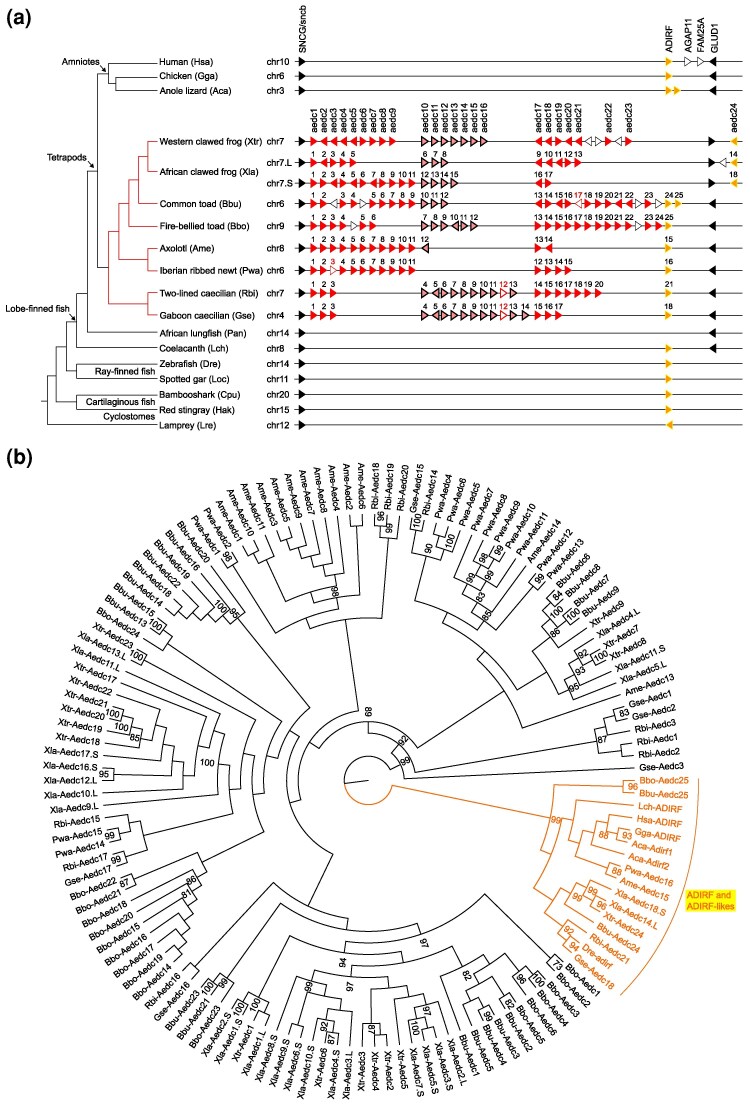
Synteny and phylogenetic analysis of the AEDC. (a) ADIRF/AEDC locus of vertebrates. Genes are depicted as triangles that point in the direction of transcription. Red triangles with black contour indicate genes for glycine-rich AEDC proteins, and red triangles indicate genes for other AEDC proteins. Orange triangles with yellow contour indicate ADIRF-like AEDC genes. White triangles with red contour indicate mutated AEDC genes. chr, chromosome. (b) Phylogenetic analysis of AEDC proteins and ADIRF based on maximum likelihood. Glycine-rich AEDC proteins were not included. Bootstrap values above 80 are indicated on the branch nodes. Species: human (*H. sapiens*, Hsa), chicken (*G. gallus*, Gga), anole lizard (*A. carolinensis*, Aca), western clawed frog (*X. tropicalis*, Xtr), African clawed frog (*X. laevis*, Xla), common toad (*B. bufo*, Bbu), fire-bellied toad (*B. bombina*, Bbo), axolotl (*A. mexicanum*, Ame), Iberian ribbed newt (*P. waltl*, Pwa), two-lined caecilian (*R. bivittatum*, Rbi), Gaboon caecilian (*G. seraphini*, Gse), African lungfish (*P. annectens*, Pan), coelacanth (*L. chalumnae*, Lch), zebrafish (*D. rerio*, Dre), spotted gar (*L. oculatus*, Loc), brownbanded bamboo shark (*C. punctatum*, Cpu), red stingray (*H. akajei*, Hak), and far eastern brook lamprey (*L. reissneri*, Lre).

To identify potential cornification proteins of amphibians, we searched the genomes of *Xenopus tropicalis*, *X. laevis*, the common toad (*Bufo bufo*), the fire-bellied toad (*Bombina bombina*), the axolotl (*Ambystoma mexicanum*), the Iberian ribbed newt (*Pleurodeles waltl*), the two-lined caecilian (*Rhinatrema bivittatum*), and the Gaboon caecilian (*Geotrypetes seraphini*) for genes encoding proteins with sequence similarities to mammalian cornification proteins. The automated gene annotation algorithm used by GenBank (https://www.ncbi.nlm.nih.gov/refseq/annotation_euk/gnomon/, last accessed on 2026 March 31) has annotated several genes as potential homologs or cornification protein-like genes, as illustrated in Figure S1. The predicted “loricrin” of *X. laevis* (Figure S1a), the “small proline-rich protein (SPRP) 2B” of *X. tropicalis* (Figure S1b), and “KRTAP5-5” of the common toad (*B. bufo*) (Figure S1c) display sequence similarity to mammalian proteins. The similarity is mainly caused by enrichment for the same amino acid residues and the absence of complex sequence motifs. We found that all these amphibian genes are located in a gene cluster, which we termed the amphibian epidermal differentiation cluster (AEDC) (Fig. 2a). The individual genes of this cluster were termed “aedc” followed by consecutive numbers according to the position in the cluster (Tables S1 and S2), with the names of the corresponding proteins (“Aedc1” etc.) being capitalized (Figure S2). The length of AEDC proteins varies from 57 (*X. tropicalis* Aedc1) to 751 (*R. bivittatum* Aedc13) amino acid residues, but the majority of these proteins are <100 amino acid residues long (Figure S2 and Table S3).

### AEDC genes share local synteny with the *adipogenesis regulatory factor* gene

The AEDC is flanked by the genes *sncg* (*synuclein gamma*) and *glud1* (*glutamate dehydrogenase 1*) in anurans (*Xenopus* frogs, common toad, and fire-bellied toad), caudates (axolotl and Iberian ribbed newt), and caecilians (Gaboon caecilian and two-lined caecilian) (Fig. 2a). In *Xenopus* frogs, *glud1* is inverted, and one AEDC gene is located outside of the chromosomal segment ranging from *sncg* to *glud1* (Fig. 2a). The number of AEDC genes varies between species of amphibians with up to 24 genes, forming the AEDC in *X. tropicalis*. However, some of these genes contain premature stop codons, indicating pseudogenization. In the allo-tetraploid *X. laevis*, an AEDC is conserved on both chromosomes 7L and 7S (Fig. 2a).

Comparative genome analysis shows that the AEDC is syntenic with the gene *adipogenesis regulatory factor* (*ADIRF*) in amniotes and vertebrates phylogenetically basal to tetrapods ranging from the cyclostomes (jawless vertebrates) to the coelacanth (Fig. 2a). Two paralogs of *Adirf* are present in the anole lizard, whereas no gene is located between *sncg* and *glud1* in the lungfish (Fig. 2a). Human *ADIRF* is expressed in a broad range of cell types and encodes a poorly characterized protein (Maeda et al. 1996; Ni et al. 2013; Kamimura et al. 2021; Teng et al. 2023; Yuan et al. 2026), which was identified as an intrinsically disordered protein in the HaCaT keratinocyte cell line (Samulevich et al. 2022).

AEDC genes were categorized by hierarchical clustering based on the amino acid composition of the proteins that they encode (Figure S3 and Table S3). The majority of the AEDC genes code for proteins with high contents of proline, glutamine, and cysteine, similar to the examples shown in Figure S1b and c. The other AEDC genes, which are located in the center of the gene cluster in all species examined except for the Iberian ribbed newt (Fig. 2a), encode proteins with high contents of glycine, similar to the example shown in Figure S1a.

A phylogenetic analysis of AEDC proteins was possible upon exclusion of the glycine-rich subgroup of proteins, the amino acid sequences of which could not be reliably aligned to the other sequences. Instead, ADIRF proteins of amniotes were included in the analysis. Maximum likelihood-based phylogenetics revealed that one AEDC gene of each amphibian species (or two genes in the case of the common toad) and ADIRFs of nonamphibian vertebrates (Figure S4) form a monophyletic clade to the exclusion of all other AEDC genes (Fig. 2b). The ADIRF-like AEDC genes are consistently located on the 3′-side of the *glud1* gene in all species of amphibians investigated (Fig. 2a).

### Duplications of regions within exons and duplications of exons have led to amino acid sequence repeats in AEDC proteins

**Figure 3:**
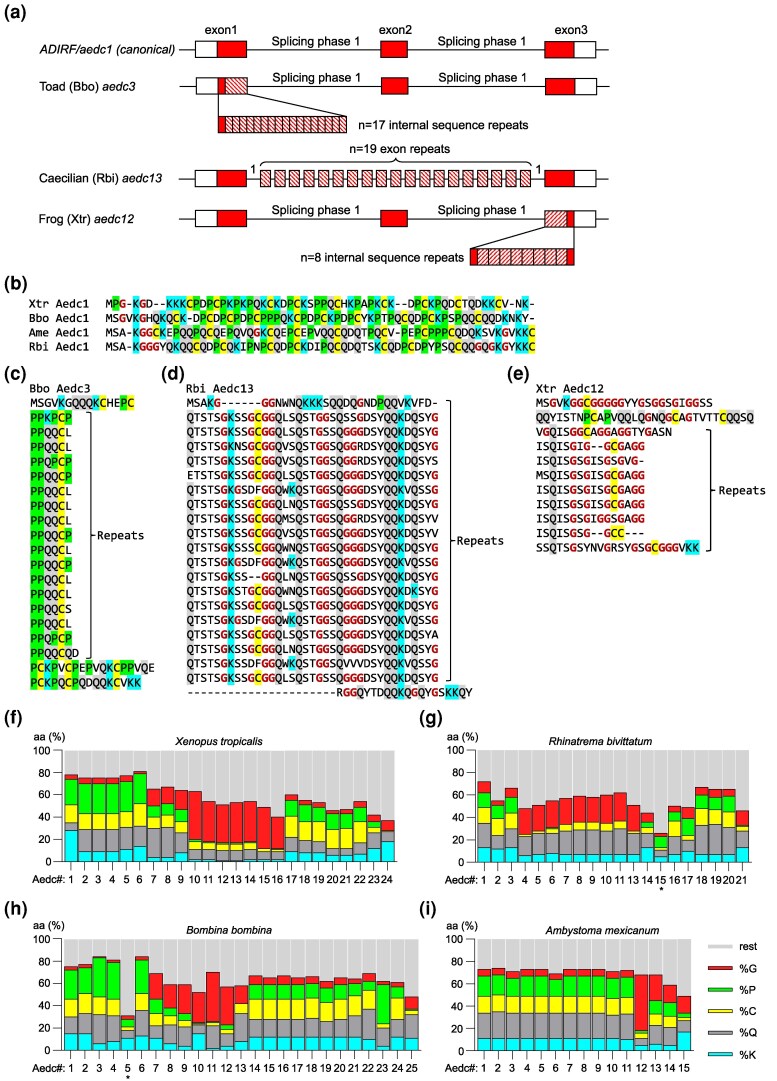
Structure of AEDC genes and amino acid sequence features of AEDC proteins. (a) Exon–intron organization and repeats of AEDC genes. Exons are depicted by boxes, in which, the coding region is depicted in red or in with red stripes indicating repeats. Introns are depicted by horizontal lines. (b) Amino acids sequences of AEDC proteins. Potential sites of cross-linking (cysteine [C], glutamine, Q; lysine, K) and abundant residues (proline, P; glycine, G) are highlighted by colors. The presence of sequence repeats (c–e) is indicated by line breaks after each repeat. The amino acid composition of all AEDC proteins of *X. tropicalis* (f), *R. bivittatum* (g), *B. bombina* (h), and *A. mexicanum* (i) is shown by bar graphs. Species: western clawed frog (*X. tropicalis*, Xtr), fire-bellied toad (*B. bombina*, Bbo), axolotl (*A. mexicanum*, Ame), two-lined caecilian (*R. bivittatum*, Rbi). Detailed information about amino acid contents is provided in Table S4.

The analysis of the exon–intron structures showed that most AEDC genes have the same structure as *ADIRF* genes, with three exons all of which are spliced in phase 1 (after the first nucleotide of a codon) during mRNA maturation (Fig. 3a). Deviations from this canonical exon–intron structure are caused by the amplification of exon 2, as exemplified by the *aedc13* gene of the two-lined caecilian (*R. bivittatum*) (Fig. 3a). Independent amplifications of internal sequence regions within exons 1 and 3 are present in *aedc3* of the fire-bellied toad (*B. bombina*) and *aedc12* of *X. tropicalis*, respectively (Fig. 3a).

The absence or presence of nucleotide sequence repeats within AEDC genes as described above (Fig. 3a) is associated with major differences in amino acid sequences of the corresponding proteins (Fig. 3b–e). Aedc1 proteins (Fig. 3b) are short (57–63 amino acid residues) and display low sequence complexity due to high contents of cysteine, glutamine, lysine, and/or proline (Fig. 3b). The lengths of *B. bombina* Aedc3 (Fig. 3c), *R. bivittatum* Aedc13 (Fig. 3d), and *X. tropicalis* Aedc12 (Fig. 3e) are increased by the presence of imperfect repeats of 6 to 37 amino acid residues (Fig. 3c–e) due to the repeats in different parts of the corresponding genes (Fig. 3a). Similar repeats are also present in many other AEDC proteins (Figure S2).

The amino acid composition of AEDC proteins shows a bias toward few specific residues and generally differs between the glycine-rich proteins and the remaining AEDC proteins in *X. tropicalis* (Fig. 3f), *R. bivittatum* (Fig. 3g), *B. bombina* (Fig. 3h), and *A. mexicanum* (Fig. 3i). The extremely high glycine content of axolotl Aedc12 (49.7% glycine) exceeds that of the most glycine-rich human EDC protein, loricrin (46.5% glycine). The three amino acids involved in covalent protein cross-linking during cornification (Candi et al. 2005; Holthaus et al. 2025), ie cysteine, glutamine, and lysine, are abundant in AEDC proteins and represent around 30%–50% of the total amino acids in the glycine-poor group of AEDC proteins. The spectrum of amino acid compositions of AEDC proteins is similar to that of EDC proteins of amniotes (Strasser et al. 2014).

### AEDC genes are specifically expressed in the skin

**Figure 4:**
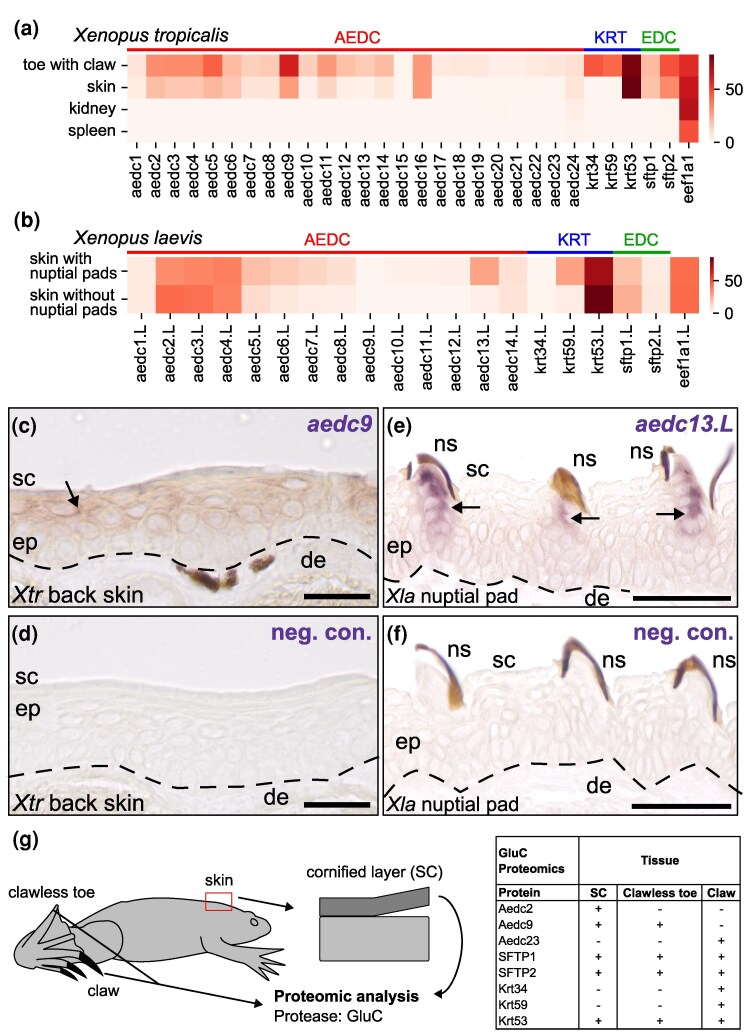
AEDC genes are expressed in the skin and skin appendages of *Xenopus* frogs. Expression levels of the AEDC genes of the western clawed frog (*X. tropicalis*) (a) (Table S5) and the African clawed frog (*X. laevis*) (b) (Tables S6 and S7) were determined by the analysis of RNA-seq data. The heatmaps show the cube root-transformed mean of read counts of three biological replicates. Only data of AEDC genes of chromosome 7.L are shown here, whereas RNA-seq results of AEDC genes of chromosome 7.S are shown in Figure S5. (c) mRNA in situ hybridization of *aedc9* in the back skin of *X. tropicalis* (Xtr). Purple staining indicates expression of *aedc9* (arrow). (d) Negative control (neg. con.) experiment using *aedc9* sense probe. (e) mRNA in situ hybridization of *aedc13.L* in the nuptial pad of the forelimb of *X. laevis* (Xla). Purple staining indicates expression of *aedc13.L* (arrows). (f) Negative control (neg. con.) experiment using *aedc13.L* sense probe. Dashed lines indicate the border between the epidermis and the dermis. Abbreviations: de, dermis; ep, epidermis; ns, nuptial spines; sc, stratum corneum. Scale bars, 20 μm (c and d), 50 μm (e and f). (g) Proteomic analysis using GluC protease for protein digestion. The detection of proteins is summarized in a table with “+” and “–” indicating presence and absence, respectively, of the proteins.

Next, we determined the tissue expression pattern of AEDC genes in *Xenopus* frogs. We analyzed RNA-seq datasets from clawed toes (Carron et al. 2024) and the skin and other organs of *X. tropicalis* (Fig. 4a), and we performed RNA sequencing of skin with cornified nuptial pads in comparison to skin without nuptial pads of *X. laevis* (Fig. 4b). AEDC genes are expressed at high levels in the skin and only at minute levels in internal organs (Fig. 4a). Like keratins *krt34* and *krt59* (Carron et al. 2024), several AEDC genes are expressed at higher levels in the clawed toes than in skin lacking hard cornified structures (Fig. 4a). Similarly, some of the AEDC genes of *X. laevis* are expressed at higher levels in the nuptial pads than in skin without nuptial pads (Fig. 4b; Figure S5).

To test the hypothesis that the expression of AEDCs is associated with epithelial cornification, we performed mRNA in situ hybridization for two AEDC genes that were selected to represent expression independent of hard skin appendages (*X. tropicalis aedc9*, Fig. 4a) or expression linked to hard skin appendages (*X. laevis aedc13.L*, Fig. 4b). The mRNA of *aedc9* was detected in the suprabasal layers of the epidermis, where keratinocytes undergo differentiation toward the stratum corneum (Fig. 4c). *aedc13.L* was expressed in keratinocytes differentiating into the cornified tips of epithelial spines within the nuptial pads, recently shown to be an expression site of hair keratin homolog *krt59* (Steinbinder et al. 2026) but not in the epidermis between the spines (Fig. 4e). Negative controls confirmed the specificity of the detection (Fig. 4d and f). These data show that AEDC genes are expressed at sites of epithelial cornification and individual AEDC genes differ in their expression patterns.

To substantiate the association of the AEDC with cornification at the protein level, we performed mass spectrometry-based proteomics on tissue samples from *X. tropicalis*. We investigated the cornified layer (stratum corneum) of the epidermis after its separation from the underlying living layers and toes bearing cornified claws (toes I through III on the hindlimbs) in comparison to toes lacking claws (toes IV and V on the hindlimbs). In the isolated stratum corneum, peptides corresponding to Aedc2 and Aedc9 were detected (Fig. 4g; Figure S6). Clawed toes but none of the other samples contained Aedc23, whereas Aedc9 was detected in the clawless toes but not in the claw-bearing toes (Fig. 4g; Figure S6). These results demonstrate that AEDC proteins of *Xenopus* frogs are incorporated into cornified epithelial structures.

**Figure 5:**
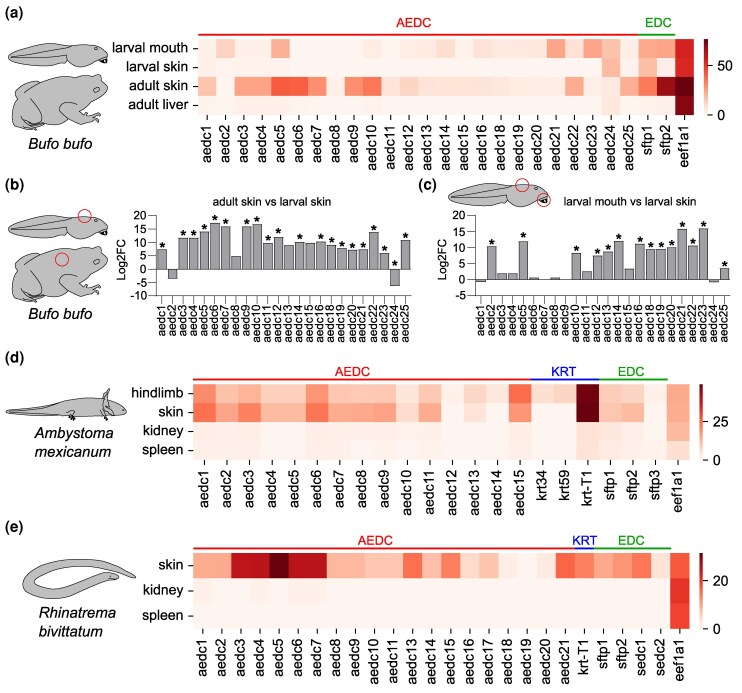
AEDC genes are specifically expressed in the skin and skin appendages across amphibians. The expression levels of the AEDC genes were determined in tissues of the common toad (*B. bufo*) (a–c), axolotl (*A. mexicanum*) (d), and the two-lined caecilian (*R. bivittatum*) (e). RNA-seq data from larval tissues of *B. bufo* (determined in this study) and datasets previously generated by us and others were used to generate heatmaps (Tables S8–S10). The scales on the right show cube root-transformed mean of read counts (a and d) and transcripts per million (e). Selected keratin (KRT), EDC genes, and the housekeeping gene *eef1a1* are included for comparison (a, d, and e). The expression levels of AEDC genes in adult versus larval skin (b) and larval mouth including oral apparatus versus larval skin (c) were compared (larval tissues *n* = 3, adult tissues, *n* = 5) (Tables S11 and S12). Log2 fold changes (FC) are shown in bar charts (b and c). *, adjusted *P*-value <0.05.

Next, we investigated AEDC expression in the common toad (*B. bufo*), which forms a cornified oral apparatus during its tadpole stage of development (Bonacci et al. 2008). The larval mouth and skin were subjected to RNA sequencing, and the results were compared to RNA-seq data of adult toads (Fig. 5a). Most AEDC genes were expressed at higher levels in the cornified adult skin than in the noncornified larval skin (Fig. 5a and b). The expression of 14 out of 25 AEDC genes was also higher in the larval mouth (containing cornified epithelium) than in the noncornified larval skin (Fig. 5a and c). The analysis of RNA-seq data from the axolotl (*A. mexicanum*) (Fig. 5d) and the two-lined caecilian (*R. bivittatum*) (Fig. 5e) confirmed that AEDC genes are also expressed in the skin but not or only at minute levels in internal organs of nonanuran amphibians. Like in *X. tropicalis* (Fig. 4a) and the common toad (Fig. 5a), the AEDC genes were coexpressed with keratins and EDC genes in both the axolotl (Fig. 5d) and the caecilian (Fig. 5e).

## Discussion

Cornification is a cell differentiation process and a mechanism for the production of protein-dependent biomaterials within durable structures that serve critical functions in the protection against the environment (cornified layer of the epidermis, hair, and feathers) and in defensive or aggressive interactions with other organisms (horns, claws, beaks, and horny teeth). The products of epithelial differentiation genes play central roles in building and stabilizing cornified proteinaceous structures, and, accordingly, the identification and characterization of these genes are important for the understanding of cornification in the various biological contexts (Candi et al. 2005; Bornschlögl et al. 2016; Plowman 2018; Holthaus et al. 2025; Sachslehner et al. 2025; Parry 2026). The present study identifies genes for a previously unknown class of epidermal differentiation-associated proteins, which share amino acid sequence features with established cornification proteins of the EDC and KRTAP families. However, the evolution of these genes from an ancestral gene unrelated to the evolutionary precursors of EDC and KRTAP genes and their confinement to amphibians make AEDC proteins unique.

**Figure 6:**
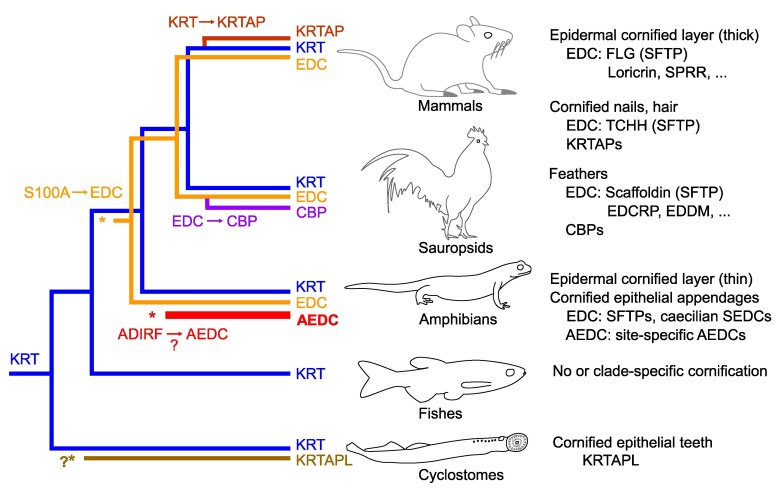
Evolution of epidermal differentiation-associated proteins in vertebrates. The origin (*) and further diversification of structural protein families in diverse vertebrate lineages is depicted by phylogenetic trees. A question mark indicates uncertainty about the origin of KRTAPLs of cyclostomes. The main expression sites are listed beside the animal symbols. KRT, keratin; EDC, epidermal differentiation complex; KRTAP, keratin-associated protein; CBP, corneous beta protein; AEDC, amphibian epidermal differentiation cluster.

We propose a model for the evolution of AEDC proteins in comparison to known cornification proteins, as depicted in Fig. 6. Previous studies have defined the origin of EDC proteins in a common ancestor of tetrapods (Mlitz et al. 2017; Sachslehner and Eckhart 2022), the origin of KRTAPs in mammals (Khan et al. 2014; Holthaus et al. 2025), and the origin of KRTAPLs in an ancestor of modern cyclostomes (Sachslehner et al. 2025). Based on the distribution of the AEDC in extant species, we conclude that this gene cluster originated in a common ancestor of amphibians (Fig. 6). The local synteny of the gene locus (Fig. 2a), identical exon–intron structure (Fig. 3a), and sequence similarity (Figure S4) suggest that the AEDC may have evolved by duplication of an ancestral *ADIRF* gene in stem amphibians and subsequent duplications of the derived gene during the evolution of amphibians. The sequence differences among AEDC proteins (Fig. 3) indicate that AEDC genes have undergone diversifying evolution, which appears to have occurred in parallel to the diversification of EDC genes in amniotes and KRTAPs in mammals. The differences in the numbers of genes for glycine-rich and other AEDC proteins in the eight species of amphibians investigated in the present study suggest lineage-specific gene duplications and/or gene loss in the AEDC. In view of the large number of species of amphibians (*n* > 9,000, https://amphibiaweb.org/amphibian/speciesnums.html, last accessed on 2026 July 12), an expansion of taxon sampling is required in follow-up studies to achieve a comprehensive analysis of the evolution of the AEDC gene family.

The identification of more than 20 AEDC proteins greatly expands the repertoire of epidermal differentiation-associated proteins in *X. tropicalis*, the main diploid model species of amphibians. Two EDC proteins, named SFTP1 and SFTP2, were predicted previously (Mlitz et al. 2017) and confirmed to be present in the stratum corneum of *X. tropicalis* in the present study (Fig. 4g). AEDC proteins differ from SFTPs by the lack of an S100 domain, thereby resembling EDC proteins, such as loricrin and SPRPs, which do not have an ortholog in frogs (Sachslehner and Eckhart 2022). Differences in the abundance of residues suitable for covalent and noncovalent binding indicate differences in protein:protein interactions of individual AEDC proteins. Due to their low sequence complexity, AEDC proteins—like EDC proteins and KRTAPs—are not predicted to fold into stable three-dimensional structures but rather to contain intrinsically disordered regions (Shamilov et al. 2021). The structural flexibility of disordered regions may be important for establishing stable interactions of cornification proteins with keratin filaments and other proteins, serving either as a matrix between cytoskeletal proteins or a proteinaceous envelope at the cell periphery.

The differential expression of individual AEDC genes at different skin sites suggests mechanisms to control the transcription of the genes in concert with other body site-specific epithelial differentiation-associated genes. Recently, we reported that the expression of hair keratin homologs and the development of cornified claws and cornified nuptial pads depend on the transcription factor Hoxc13 in *X. tropicalis* (Carron et al. 2024; Steinbinder et al. 2026). Reanalysis of the transcriptomes of normal claw-bearing toes versus their clawless counterparts of Hoxc13-deficient frogs showed that the expression of *aedc23* is decreased approximately 1000-fold in the absence of Hoxc13. The neighboring gene *aedc22* was also significantly downregulated whereas *aedc7* was slightly but significantly upregulated in Hoxc13-deficient toes (Figure S7a and Table S13). The proximal promoter of *aedc23* contains a potential binding site of Hoxc13, which is conserved in the orthologous genes of five other frogs of the *Pipidae* family (Figure S7b). These data suggest that *aedc23* may be a direct target of transcriptional regulation by Hoxc13 to induce its expression in parallel to that of the hair keratin homologs *krt34* and *krt59*. Further studies are needed to test this hypothesis. Importantly, in situ hybridization experiments demonstrate that the expression of *aedc13.L* (Figure 4e) colocalizes with that of the known cornification gene *krt59* (Steinbinder et al. 2026) in the nuptial pads of *Xenopus* frogs, confirming that Aedc13.L is a hard cornification-associated protein.

The present study has several limitations due to features of AEDC proteins that are shared with EDC proteins and KRTAPs. The biased amino acid composition, low sequence complexity, and the presence of sequence repeats indicate that these proteins are intrinsically disordered, which means that the analysis of their evolution requires an approach different from that established for proteins with stable domains (Daughdrill et al. 2007; Cascarina and Ross 2025; Mughal and Caetano-Anollés 2025). The independent emergence of the aforementioned features in AEDC and EDC proteins as well as KRTAPs suggests a form of convergent evolution, but this hypothesis requires further investigation. The limited length and the low complexity of amino acid sequences complicate not only the comparison between different groups of cornification-associated proteins. They also make it difficult to obtain sequence alignments useful for the analysis of phylogenetic relationships within the AEDC protein family. Accordingly, the results of molecular phylogenetics do not fully inform about the routes of evolutionary diversification of AEDC genes in amphibians (Fig. 2b). The sequence similarities to mammalian EDC proteins and KRTAPs (Figure S1) and the presence in cornified structures (stratum corneum, claws, nuptial pads, and oral apparatus of tadpoles) (Figs. 4 and 5) suggest functions of AEDC proteins in epithelial cornification. However, it remains to be determined whether AEDC proteins mainly function as protein components of the mature cornified material (equivalent to loricrin in human epidermal corneocytes) or as regulators of a terminal differentiation step of keratinocytes (equivalent to filaggrin in human epidermal corneocytes). Studies of mammalian EDC proteins have shown that the experimental investigation of individual proteins in epithelial cornification is difficult (Hohl et al. 1991; Smith et al. 2006). Gene knockout studies need to consider the existence of multiple paralogs, which may compensate the loss of a single member of a cornification protein family (Jarnik et al. 2002; Ishitsuka et al. 2016).

## Conclusion

AEDC proteins have evolved features similar to those of other proteins implicated in skin epithelial differentiation, namely EDC proteins, KRTAPs and KRTAPLs. The most significant shared traits of these protein families are low sequence complexity, diversification into subfamilies with differentially biased amino acid compositions, and specific expression in stratified and cornified epithelia. As the common features of AEDC proteins, EDC proteins, KRTAPs, and KRTAPLs have emerged independently in different phylogenetic lineages, these features appear to be important for epithelial cornification. The insights into the evolution of epithelial differentiation-associated proteins help to guide future studies of the molecular mechanisms by which living cells are converted into resilient and stable cornified epithelial structures.

## Materials and methods

### Investigation of the ADIRF/AEDC locus in vertebrate genomes

AEDC genes were initially identified by searching for amphibian genes that had been annotated as “SPRP-like”, “KRTAP-like” or “loricrin” by the GNOMON algorithm in GenBank (https://www.ncbi.nlm.nih.gov/refseq/annotation_euk/gnomon, last accessed on 2026 March 31). The loci these genes were compared and found to belong to a single gene cluster conserved at least in *X. laevis*, *X. tropicalis*, and *B. bufo*. Subsequently, the amino acid sequences of the proteins encoded by these genes were used as queries in BLAST searches (https://blast.ncbi.nlm.nih.gov/Blast.cgi, last accessed on 2026 March 31), in which, the filter for low sequence complexity was deactivated. The genomes of the following amphibians were investigated: western clawed frog (*X. tropicalis*, GCF_000004195.4, Mitros et al. 2019), African clawed frog (*X. laevis*, GCF_017654675.1, International Xenopus Sequencing Consortium), common toad (*B. bufo*, GCF_905171765.1, Wellcome Sanger Institute), fire-bellied toad (*B. bombina*, GCF_027579735.1, Vertebrate Genomes Project), axolotl (*A. mexicanum*, GCF_040938575.1, Smith et al. 2019), Iberian ribbed newt (*P. waltl*, GCF_031143425.1, Brown et al. 2025), Gaboon caecilian (*G. seraphini*, GCF_902459505.2, Wellcome Sanger Institute), and two-lined caecilian (*R. bivittatum*, GCF_901001135.1, Vertebrate Genomes Project). The genomes of the following vertebrates were also investigated: human (*Homo sapiens*, GCF_000001405.40, International Human Genome Sequencing Consortium), chicken (*Gallus gallus*, GCF_016699485.2, Vertebrate Genomes Project), green anole lizard (*Anolis carolinensis*, GCF_035594765.1), African lungfish (*Protopterus annectens*, GCF_019279795.1, Wang et al. 2021), coelacanth (*Latimeria chalumnae*, GCA_037176945.1, Vertebrate Genomes Project), zebrafish (*Danio rerio*, GCF_049306965.1, National Human Genome Research Institute), spotted gar (*Lepisosteus oculatus*, GCF_040954835.1, Vertebrate Genomes Project), brownbanded bamboo shark (*Chiloscyllium punctatum*, GCF_047496795.1, Niwa et al. 2025), red stingray (*Hemitrygon akajei*, GCF_048418815.1), and Far Eastern brook lamprey (*Lethenteron reissneri*, GCF_015708825.1, Zhu et al. 2021). Where available in GenBank, the mapping of RNA-seq reads against genome sequences was used to predict exons of AEDC genes, leading to corrections relative to the automated exon predictions in GenBank. The corrections are summarized in Supplementary Tables S1 and S2. The coding sequence coordinates of the amphibian AEDCs are available in Table S1. Manually corrected coordinates of exons are available in Table S2. The amino acid sequences of AEDC proteins are provided in Figures S2, respectively.

### Amino acid composition analysis of AEDC protein sequences

The amino acid contents of AEDC proteins (Figure S2) were subjected to hierarchical clustering using the Python package/module seaborn (version: 0.13.2) implemented in Python (version: 3.13), and the results were visualized as a cluster map (Table S14). Bar charts were designed using GraphPad Prism 8 (Version: 8.0.1).

### Amino acid sequence alignments and molecular phylogenetics

The amino acid sequences of AEDC proteins (Figure S2) were subjected to a multiple sequence alignment using MAFFT (Katoh et al. 2002) with the settings --maxiterate 1000 and --localpair. The tool Block Mapping and Gathering with Entropy (Criscuolo and Gribaldo 2010) was used with a BLOSUM30 matrix to automatically trim the alignment (Figure S8). The amino acid substitution model according to Jones, Taylor, and Thornton (JTT) (Jones et al. 1992) was determined as the best fitting amino acid substitution model. A phylogenetic analysis based on maximum likelihood was performed with IQ-TREE 2, setting 1,000 bootstraps (version: 2.4.0, Minh et al. 2020). FigTree (http://tree.bio.ed.ac.uk/software/figtree/, last accessed on 2026 February 2) was used to visualize the phylogenetic trees, which were further edited with Inkscape (version 1.0.0.0; https://inkscape.org/de/, last accessed on 2026 February 2). Sequence alignments shown in the figures were generated with MUSCLE, provided by EMBL-EBI (https://www.ebi.ac.uk/jdispatcher/msa/muscle, last accessed on 2026 February 2).

### Tissue sampling

Skin samples from *X. tropicalis* were available from a previous study (Carron et al. 2024). Tissues of *X. laevis* were freshly dissected and fixed in 7.5% neutral-buffered formalin (catalog number 18727.00500, Morphisto, Germany) over night and subsequently embedded in paraffin.

### RNA extraction from *X. laevis* and cDNA synthesis

Freshly dissected tissue of adult *Xenopus* frogs and common toad tadpoles was placed in RNA-later (Invitrogen), stored first over night at 4 °C, and afterward at −80 °C until RNA extraction. RNA was isolated with the Trizol reagent (VWR) from homogenized tissues according to a published protocol (Chomczynski 1993). cDNA was synthesized with the iScript cDNA synthesis kit (Quantabio).

### RNA sequencing of common toad tadpoles

Three samples of the oral apparatus and three dorsal skin samples of common toad tadpoles were subjected to RNA sequencing according to a published protocol (Carron et al. 2024) at the Core Facility Genomics, Medical University of Vienna. Briefly, sequencing libraries from total RNA of the samples were prepared with the NEBNext Poly(A) mRNA Magnetic Isolation Module and the NEBNext UltraExpress RNA Library Prep Kit for Illumina with unique dual indices according to the manufacturer's protocols (New England Biolabs, Ipswich, MA, USA). The quality of the libraries was controlled on a Bioanalyzer 2100 (Agilent, Santa Clara, CA, USA) using a High Sensitivity DNA Kit (Invitrogen, Waltham, USA). The libraries were sequenced on a P4 flowcell of a NextSeq2000 instrument in 1 × 50 bp single end sequencing mode. On average, 22 million reads per sample were generated. The Illumina bcl2fastq command line tool (Illumina, v2.19.1.403) was used to generate the fastq files. The raw reads were uploaded to NCBI GenBank and are available under the accession number PRJNA1335918.

### RNA-seq analysis of *X. tropicalis*, *X. laevis*, *B. bufo*, and *R. bivittatum*

Using the AEDC gene sequences predicted in this study (Tables S1 and S2 and Figure S9), we analyzed the RNA-seq datasets of the following species: western clawed frog (*X. tropicalis*, submitter: Amphibian Research Center, Hiroshima University), Hoxc13-deficient *X. tropicalis* (NCBI GenBank accession number PRJNA1055414) (Carron et al. 2024), *X. laevis* (PRJNA1335124, Steinbinder et al. 2026), axolotl (*A. mexicanum*) (Ye et al. 2022), common toad (*B. bufo*) (this study and PRJNA1019526, Chondrelli et al. 2024), and the two-lined caecilian (*R. bivittatum*) (Torres-Sánchez et al. 2019) (Table S15). The publicly available datasets were downloaded from NCBI GenBank and subsequently converted to fastq files with the prefetch and fastq-dump package from SRA Toolkit (version: 3.1.1, https://github.com/ncbi/sra-tools, last accessed on 2026 February 2), respectively. From this point on, the same procedures were applied to the datasets downloaded from GenBank and the datasets generated in this study. The read quality was controlled with FastQC (version 0.12.1, https://www.bioinformatics.babraham.ac.uk/projects/fastqc/, last accessed on 2026 February 2). Nextera adapters and Trueseq3 adapters for paired end data were removed from the RNA-sequencing data of the axolotl and the adult common toad, respectively, using Trimmomatic (version: 0.40, Bolger et al. 2014) with the following settings: ILLUMINACLIP: “adapter-file.fasta”:2:30:10:1:true, LEADING:3, TRAILING:3, MAXINFO:40:0.8, MINLEN:36. The reads were aligned to their corresponding reference genomes (*X. tropicalis*: GCF_000004195.4, *X. laevis*: GCF_017654675.1, *B. bufo*: GCF_905171765.4, *A. mexicanum*: GCF_040938575.1, and *R. bivittatum*: GCF_901001135.1) using Salmon (version: 1.10.3, Patro et al. 2017) with the setting “--keepDuplicates” to avoid filtering of AEDC genes with similar sequences. Differential gene expression analyses were performed for the following datasets: *X. tropicalis* wildtype claw-bearing toes versus corresponding toes of Hoxc13-deficient frogs (Table S13); *X. laevis* ventral forelimb skin with nuptial pads versus ventral hindlimb skin without nuptial pads (Table S6); common toad adult skin versus larval skin (Table S11); common toad larval mouths (including cornified oral apparatus) versus noncornified larval skin (Table S12). The module PyDESeq2 (Muzellec et al. 2023) implemented in Python (version: 3.11) was used for the differential gene expression analyses. The Wald test, followed by multiple testing according to Benjamini–Hochberg, was applied to assess statistical significance (Chen et al. 2011). All genes with an adjusted *P*-value <0.05 were considered significant. RNA-seq results were visualized as heatmaps with the package seaborn (version: 0.13.2) implemented in Python.

### Generation of riboprobes

The coding sequence and parts of the flanking untranslated regions of *X. tropicalis aedc9* (forward primer: 5′-CCATGTCTGCTAAAGGAAGCC-3′, reverse primer: 5′-CAGCAAGATTTTGTGAAATCAGC-3′) and *X. laevis aedc13.L* (forward primer: 5′-CCACATCTCAGTCATGTCTGG-3′, reverse primer: 5′-CTTGCAATAGTATTATGTTAGATGC-3′) were amplified by reverse transcription (RT)-PCR. The PCR products were ligated into the pGEM-T Easy vector (Promega, USA) and cloned and amplified in *E. coli* (catalog number L2001, Promega). The plasmid DNA containing the insert was isolated with the QIAprep spin miniprep kit 250 (catalog number 27106, Qiagen, Netherlands) and subsequently subjected to Sanger sequencing (Microsynth, Switzerland) with SP6 primers. Correct inserts were linearized via PCR with M13 primers (forward: 5′-CGCCAGGGTTTTCCCAGTCACGAC-3′ and reverse: 5′-CAGGAAACAGCTATGAC-3′) and transcribed with the DIG RNA labeling mix (catalog number 11175025910, Roche, Switzerland) using either T7 or SP6 RNA polymerase depending on the insert orientation in the plasmid. After in vitro transcription, the riboprobes were precipitated in ethanol supplemented with 4 M LiCl overnight at −20 °C. Afterward, the probes were washed first with 70% ethanol, followed by a wash with 100% ethanol. The probes were resuspended in RNase-free water and stored at −80 °C.

### In situ hybridization

Samples for mRNA in situ hybridization were prepared from paraffin-embedded tissues with a Microm HM 335E microtome (Zeiss, Germany) at a thickness of 6 μm on super-adhesive slides (Menzel, Germany). mRNA in situ hybridization was performed according to a published approach (Sachslehner et al. 2024) with minor modifications. Briefly, paraffin sections were heated at 60 °C for 1 h to melt the paraffin, deparaffinized in xylene (catalog number 1330-20-7, Fisher Chemical, USA) for 20 min, and subsequently hydrated in a descending ethanol series. Afterward, specimens were digested with 20 μg/ml proteinase K (catalog number 03654672103, Roche, Switzerland) for 10 min at 37 °C. To neutralize charged probe binding, specimens were then incubated for 10 min in 1% triethanolamine (catalog number 90279, Sigma-Aldrich, USA) in phosphate-buffered saline (PBS), for 5 min in 1% triethanolamine supplemented with 0.15% acetic anhydride (catalog number 21390293, Prolabo, USA), and for 5 min in 1% triethanolamine supplemented with 0.3% acetic anhydride. Specimens were postfixed with 4% paraformaldehyde in PBS for 40 min and dehydrated in an ascending ethanol series before they were dried for 1 h at room temperature. Specimens were prehybridized in hybridization buffer at 60 °C for 2 h. Corresponding sense and antisense riboprobes were prepared at a concentration of 2 ng/μl and preheated in hybridization buffer (4 M urea, catalog number 0568, VWR, USA; 5× saline sodium citrate [SSC], catalog number 10541, Roth, Germany; 100 μg/ml heparin, catalog number H3149, Sigma-Aldrich, USA; 5 mM EDTA, catalog number 80401, Roth, Germany; 1× Denhardt's block reagent, Sigma-Aldrich, D2531, USA; 100 μg/ml salmon sperm DNA, catalog number 201190, Agilent, USA; 5% dextran sulfate, catalog number D8906, Sigma-Aldrich, USA) at 85 °C for 10 min. The hybridization was performed overnight at 60 °C. Samples were washed thrice with 4 M urea and 4× SSC, once with a solution containing 4 M urea and 2× SSC, and once with 4 M urea and 1× SSC at 58 °C for 15 min each, followed by one wash with 1× SSC at 37 °C for 15 min to remove unbound probes. To prevent nonspecific anti-digoxigenin antibody binding, samples were incubated for 2 h in 0.1 M maleic acid buffer (MAB, catalog number M0375, Sigma-Aldrich, USA), pH 7.5 containing 2% skim milk (catalog number 70166, Sigma-Aldrich, USA). Subsequently, specimens were incubated with an anti-digoxigenin antibody conjugated to alkaline phosphatase (1:5,000, catalog number 11093274910, Roche, Switzerland) in 2% MAB block solution overnight at 4 °C. The samples were washed twice with alkaline phosphatase buffer for 15 min at room temperature. The staining was achieved with an alkaline phosphatase buffer (0.5 M Tris pH 9.5; 0.5 M NaCl) supplemented with 0.05 M MgCl_2_, 3.75 μl/ml 5-bromo-4-chloro-3-indolylphosphate (catalog number 11383221001, Roche, Switzerland), and 5 μl/ml nitroblue tetrazolium (catalog number 11383213001, Roche, Switzerland). Sections were mounted with Aquatex (catalog number 108562, Sigma-Aldrich, USA), and photographs were taken with an Olympus UC-90 camera mounted on an Olympus BX63 light microscope.

### Promoter analysis of AEDC genes of pipid frogs

Consensus Hoxc13 binding site sequences (Jave-Suarez et al. 2002; Carron et al. 2024) were identified in the promoters of *X. tropicalis* (GCF_000004195.4) *aedc23*, *X. laevis* (GCF_017654675.1) *aedc13.L*, and homologous genes of *Xenopus borealis* (GCA_024363595.1), *Xenopus clivii (*GCA_046118455.1), *Xenopus petersii* (GCA_038501925.1), and *Hymenochirus boettgeri* (GCA_019447015.1). The *aedc23/13.L* homolog was identified via tBLASTn (NCBI GenBank) against the corresponding genomes of the mentioned species using *X. tropicalis aedc23* as query. Subsequently, 1,000 nucleotides upstream of the start codon were extracted, and a multiple sequence alignment was generated using Clustal Omega (https://www.ebi.ac.uk/jdispatcher/msa/clustalo, last accessed on 2025 October 6) to assess the conservation of the binding sites.

### Proteomics

Tissue for proteomics was dissected freshly from adult *X. tropicalis* frogs. Claw-bearing toes were placed in lysis buffer (7.5 M Tris pH = 8, catalog number 161-0716, Bio-Rad, USA; 7 M urea, catalog number 0568, VWR, USA; 2 M thiourea, catalog number T7875, Sigma-Aldrich, USA; 4% CHAPSO, catalog number 28304, Pierce, USA), which was supplemented with dithiothreitol (DTT) to a final concentration of 0.2 M and 7× proteinase inhibitor cocktail (catalog number 11836153001, Roche, Switzerland) to a final concentration of 1×. The toes were boiled at 70 °C for 3 h on a thermoshaker before lysis. Complete skin was dissected freshly and incubated in 1× dispase II (catalog number 04942078001, Roche, Switzerland) for 30 min at 37 °C. Afterward, the epidermis was separated from the dermis with forceps. To obtain the isolated stratum corneum, the remaining living cells of the epidermis were digested with 0.05% trypsin/EDTA (catalog number 25300054, Thermo Fisher, USA) for 30 min at 37 °C. The isolated stratum corneum and the toes were homogenized with a Precellys homogenizer (VWR) using lysis tubes filled with ceramic beads (catalog number 31-00589, IST Innuscreen GmbH, Germany). After centrifugation at 18,000 × *g* for 15 min at 4 °C, the supernatant was collected, and the pellet was sonicated using a Hielscher Ultrasound Technology sonicator (amplitude 100, two times 30 s). The solution was centrifuged as before, and the resulting supernatant was pooled with the supernatant of the previous homogenization step. Protein identification was performed according to a published protocol (Holthaus et al. 2024) with the following modifications: As the standard protein digestion protocol using trypsin was predicted to yield few peptides useful for the identification of AEDC proteins, the proteins were digested with the protease GluC (catalog number V1651, Promega), which cleaves at the carboxy-terminal side of aspartic acid (D) and glutamic acid (E) (Giansanti et al. 2016). The acquired raw MS data files were processed and analyzed using ProteomeDiscoverer (v2.4.0.305, Thermo Fisher). SequestHT was used as search engine, and following parameters were chosen: database: *X. tropicalis* (GenBank accession number GCF_000004195.4), supplemented with the manually annotated AEDC amino acid sequences (Figure S2); enzyme: GluC; max. missed cleavage sites: 2; static modifications: carbamidomethyl (C); dynamic modifications: oxidation (M), acetyl (protein N-terminus), met-loss (M), met-loss+acetyl (M); precursor mass tolerance: 10 ppm; fragment mass tolerance: 0.02 Da. The precursor ion quantification was performed using the Minora Feature Detector node. The retention time alignment was performed with a maximum RT shift of 10 min and a mass tolerance of 10 ppm.

## Supplementary Material

msag196_Supplementary_Data

## Data Availability

The data underlying this article are available in the following data repositories: RNA-seq data for *X. tropicalis* (PRJDB17814), Hoxc13-deficient *X. tropicalis* (PRJNA1055414), *X. laevis* (PRJNA1335124), adult *B. bufo* (PRJNA1019526), *A. mexicanum* (PRJNA757699), and *R. bivittatum* (PRJNA757699) were downloaded from NCBI GenBank. The RNA-sequencing data generated for common toad tadpoles were uploaded to NCBI GenBank (accession number: PRJNA1335918). The proteomic data generated herein for *X. tropicalis* were uploaded to the PRIDE database and are available under the accession number (PXD073929). The read quantification and proteomic references with manual AEDC gene corrections for *X. tropicalis* are available on the Zenodo repository at https://doi.org/10.5281/zenodo.18630153. The read quantification reference file with manually added coding sequences for the axolotl is available on the Zenodo repository at https://doi.org/10.5281/zenodo.18630201. Reference genomes investigated in this study are available on NCBI GenBank under the following accession numbers: *H. sapiens* (GCF_000001405.40), *G. gallus* (GCF_016699485.2), *A. carolinensis* (GCF_035594765.1), *X. tropicalis* (GCF_000004195.4), *X. laevis* (GCF_017654675.1), *B. bufo* (GCF_905171765.1), *B. bombina* (GCF_027579735.1), *A. mexicanum* (GCF_040938575.1), *P. waltl* (GCF_031143425.1), *G. seraphini* (GCF_902459505.2), *R. bivittatum* (GCF_901001135.1), *X. borealis* (GCA_024363595.1), *X. clivii* (GCA_046118455.1), *X. petersii* (GCA_038501925.1), *H. boettgeri* (GCA_019447015.1), *P. annectens* (GCF_019279795.1), *L. chalumnae* (GCA_037176945.1), *D. rerio* (GCF_049306965.1), *L. oculatus* (GCF_040954835.1), *C. punctatum* (GCF_047496795.1), *H. akajei* (GCF_048418815.1), and *L. reissneri* (GCF_015708825.1). The study of *X. tropicalis* was conducted in accordance with the guidelines and regulations of Ghent University, Ghent, Belgium. The project was approved by the Ethical Committee for Animal Experimentation, Ghent University, Faculty of Sciences (approval number EC2023-041). *X. laevis* frogs were kindly provided by the laboratory of Elly Tanaka (Institute of Molecular Biotechnology of the Austrian Academy of Sciences, Vienna). Tadpoles of the common toad (*B. bufo*) at developmental stage 31 (Gosner 1960) were collected from a pond (coordinates: 48°42′46.2″N 15°39′35.2″E) under permission by the government of Lower Austria, Amt der Niederösterreichischen Landesregierung, Abteilung Naturschutz, 3109 St. Pölten, Austria (license number: RU5-BE-2095/001-2024). Ethyl-3-aminobenzoate methanesulfonate (MS-222, catalog number E10521 Sigma-Aldrich, USA) at a concentration of 1 g/l (Vleminckx 2018) was used to sacrifice the frogs and common toad tadpoles, which is in line with the local laws of Austria and the guidelines of the Medical University of Vienna. No experiments were performed on live animals.
