# Supplementary material for: Evolution of an amphibian-specific family of epidermal differentiation-associated genes
Source: Mol Biol Evol. 2026 Aug 6;43(8):msag196. doi: 10.1093/molbev/msag196 (PMC13487633; doi:10.1093/molbev/msag196)
Supplement: Supplementary file 1 [file msag196_supplementary_data.zip › Supplementary-figures.pdf]

Supplementary Figures

Evolutionary origin and diversification of amphibian-specific epidermal differentiation proteins

Attila Placido Sachslehner, Julia Steinbinder, Anja Wagner, Klaus Kratochwill, Leopold Eckhart

Content:

Supplementary Figures S1-S9

A

```
Hsa Loricrin, NP_000418.2 loricrin [Homo sapiens]
Xla Aedc7L, XP_018080246.2 "loricrin" [Xenopus laevis]

Hsa Loricrin      MSYQKKQPTPQPPVDCVKTSGGGGGGGGSGGGGCGFFGGGGSGGGSSGSGCGYSGGGGYS
Xla Aedc7L        MS-------------------GVKGGCGGGGSGC----GKSSDQYKNTNPC--APVQQYQ
                  **                   *  ** *.**.**    * .*.  .. . *  :    *.

Hsa Loricrin      GGGCGGGSSGGGGGGGIGGCGGGSGGSVKYSGGGGSSGGGSGCFSSGGGGSGCFSSG-GG
Xla Aedc7L        GNNIVQGCT-----GTLNPCQQGQVGQISGSGGANISQISGGCI---GGSAGANTSQISG
                  *..   *.:     * :. *  *. *.:. ***.. *  ..**:   **.:*. :*  .*

Hsa Loricrin      GSSGGGSGCFSSGGGGSSGGGSGCFSSGGGGFSGQAVQCQSYGGVSSGGSSGGGSGCFSS
Xla Aedc7L        GCTGG------SGAGDTSQISGSCIGGSGG-----------YGTSQISGSGGYGTSQISG
                  *.:**      **.*.:*  ...*:...**           **  . .**.* *:. :*.

Hsa Loricrin      GGGGGSVCGYSGGGSGCGGGSSGGSGSGYVSSQQVTQTSCAPQPSYGGGSSGGGGSGGSG
Xla Aedc7L        SGGYGT--------SQISGGCSGGSGGG-----------CSSQISGSGGSYG--------
                  .** *:        *  .**.*****.*           *:.* * .*** *

Hsa Loricrin      CFSSGGGGGSSGCGGGSSGIGSGCIISGGGSVCGGGSSGGGGGGSSVGGSGSGKGVPICH
Xla Aedc7L        ----------SGCGG-----------------VTGGQNGGGGA-----------------
                            *****                  **..****.

Hsa Loricrin      QTQQKQAPTWPSK
Xla Aedc7L        -----------KK
                             .*
```

B

```
Hsa SPRR2B, NP_001375127.1 small proline-rich protein 2B [Homo sapiens]
Xtr Aedc19, XP_031762298.1 small proline-rich protein 2B-like [Xenopus tropicali

Xtr Aedc19       MSGVKGGRSHCHQKTYCPDPCQSQVVCKDPCHPQVVCKDPCHPQVVCKDPCQPLVVCKDP
Hsa SPRR2B       MS-------------------YQQQQCKQPCQPPPVCPTP-----KCPEPCPP-PKCPEP
                 **                  .*   **:**:*  **  *      * :** *   * :*

Xtr Aedc19       CELAICQDPCQPAQHYHCNTVHCTQKV-HCDPCNPCNPCHRYGKY
Hsa SPRR2B       CPPPKCPQPCPPQQ--------CQQKYPPVTPSPPCQPKYPPKSK
                 *  . * :** * *        * **      *. **:* :    .
```

C

```
Hsa KRTAP5-5, NP_001001480.2 keratin-associated protein 5-5 [Homo sapiens]
Bbu Aedc22, XP_040293943.1 "keratin-associated protein 5-5-like" [Bufo bufo]

Hsa KRTAP5-5      MGCCGCSGGCGSGCGGRGSGCGGCGSGCGGCGSGCGGCGSGCGGCGGCGSGCAGCGGCGS
Bbu Aedc22        ------------------------------------------------------------


Hsa KRTAP5-5      GCCVPVCCCKPMCCCVPACSCSSCGKGGCGSCGGSKRGCVSCGVSKGACGSCGGSKGGCG
Bbu Aedc22        ---------------------------------------------------MSGVKGGHQ
                                                                      * ***

Hsa KRTAP5-5      SCGGSKGGCGSCGGSKGGCGSCGGSKGGCGSYGCSQSSCCKPCCCSSGC-GSSCCQSSCC
Bbu Aedc22        KAQ-CKDPCQKQTICQDPCQKQTICQDPCQKQTICQDPCQKQTICQDPCQKQTICQDPCQ
                      .*  * .       * .       * .   .*..* *   *.. *   .: **..*

Hsa KRTAP5-5      KPYCCQSSCCKPYCCQSSCCKPCSCFSGCGSSCCQSSCYKPCCCQSSCCVPVCCQCKI
Bbu Aedc22        KQTICQDPCQKQTICQDPCQDPCKPVQQCKDPC------NPCVPD-----PCQSQGKY
                  *   **..* *   **..* .**. .. * ..*      :**  :      *  .* *
```

Figure S1. The automated gene annotation algorithm of GenBank identifies proteins with amino acid sequence similarities to epidermal differentiation complex (EDC) proteins and keratin-associated proteins (KRTAPs). Amphibian proteins similar to mammalian cornification proteins were identified by their current annotation in GenBank. The amino acid sequences of three proteins encoded by genes of the amphibian epidermal differentiation cluster (AEDC) in the Western clawed frog (Xtr, *Xenopus tropicalis*) (**A**), the African clawed frog (Xla, *X. laevis*) (**B**), and the common toad (Bbu, *Bufo bufo*) (**C**) were aligned to those of human (Hsa, *Homo sapiens*) loricrin (**A**), small proline-rich protein 2B (**B**), and keratin-associated protein 5-5 (KRTAP5-5) (**C**). Accession numbers and current annotations are provided above the alignments. "*", ":" and "." below the sequences indicate identity, high similarity and low similarity, respectively, of amino acid residues.

A

>Xtr-Aedc1
MPGKGDKKKCPDPCPKPKPQKCKDPCKSPPQCHKPAPKCKDPCKPQDCTQDKKCVNK

>Xtr-Aedc2
MSGVKGQQKKCPEPCPPQPCNPCPPPQKCQDPCKDPCPKPIPQCQDPCQRTPTQQQGQNQPHHH

>Xtr-Aedc3
MSGVKGQQKKCPEPCPPQPCNPCPPPQKCQDPCKDPCPKPIPQCQDPCQRTPTQQQGQNQPHHH

>Xtr-Aedc4
MSGVKGQQKKCPEPCPPQPCNPCPPPQKCQDPCKDPCPKPIPQCQDPCQRTPTQQQGQNQPHHH

>Xtr-Aedc5
MSGVKGQQKKCPEPCPPQPCNPCPPPQKCQDPCKDPCPKPIPQCQDPCQKTPTQQQGQNQPHHH

>Xtr-Aedc6
MSGVKGQQKKCQEQCPPPCDPCAPKCPPPQKCDPCPPPQKCDPCAPKCPPPQKCDPCAPKCCPPQQKCQDPCAKQVPQCQDPCKQQQH

>Xtr-Aedc7
MSAKGGSQQYKDPCVQVQPCQGTQVQPCQGTQVQPCQGVQVQPCQPQVDPCVQSNPCPTQCQGQSGQVTYTGGCQTQGQGYGGQSYGSAKK

>Xtr-Aedc8
MSAKGGSQQYKDPCVQVQPCQGTQVQPCQGTQVQPCQGIQVQPCQPQDPCVQSNPCPTQCQGQSGQVTYTGGCQTQGQGYGQSCGSAKK

>Xtr-Aedc9
MSAKGSQCKDPDPCHPVVQCQDPKPQCKDPCAPVDPCQQQGQGHGQGHQGHHHHHHQHGGQSGQGCGGAKK

>Xtr-Aedc10
MSGAKGGSGIIGGGGCGGSSGSSGGLGCGGSGAISTQCGNTGINIPVQPCQGTVQQGGTANPCLQSQGGGSFISGGGSSYGGGCGGQIGGSSYGVGCGGQTGGSQIS
GSSCGGGCGGQTGGSGYGGGCGGQTGGSGYGGGCGGQTGGGSAKK

>Xtr-Aedc11
MSGVKGGCGGGGGYYGSGGSGIGGSSQQYISTNPCAPVQQLQGNQGCAGTVTTCQQGQVGQISGGCAGGAGGTYGASNISQISGTGICGSGAGGISQISGSGISGCG
AGGISQISGSGISGCGAGGIGQISGSGIGGSGAGGISQISGSGICGSGAGGISQISGSGGCSSSQTSGSYNVGGSYGSGCGGGVKK

>Xtr-Aedc12
MSGVKGGCGGGGGYYGSGGSGIGGSSQQYISTNPCAPVQQLQGNQGCAGTVTTCQQSQVGQISGGCAGGAGGTYGASNISQISGIGGCGAGGISQISGSGISGSGVG
MSQISGSGISGCGAGGISQISGSGISGCGAGGISQISGSGISGCGAGGISQISGSGIGGSGAGGISQISGSGGCCSSQTSGSYNVGRSYGSGCGGGVKK

>Xtr-Aedc13
MSGVKGGCGGGGGYYGSGGSGIGGSSQQYISTNPCAPVQQLQGNQGCAGTCQQGQVGQISGGCAGGAGGTYGASNISQISGIGGSGLGGINQISGSGISGCGAGISQ
ISGSGISGCGAGISQISGSGISGCGAGGISQISGSGIGGCGTGGISQISGSGICGSGAGGISQISGSGGCCSSQTSGSYNVGGSYGSGCGGGVKK

>Xtr-Aedc14
MSGVKGGCGGGGGYYGSGGSGIGGSSQQYISTNPCAPVQQLQGNQGCAGTVTTCQQGQVGQISGGCTGGAGGTYGASNISQISGIGGSGVGISQISGSGISGCGAGG
ISQISGSGISGCGAGGISQISGSGIGGCGTGGISQISGSGIGGCGTGGISQISGSGGCCSSQTSGSYNIGGSYGSGCGGGVKK

>Xtr-Aedc15
MSGVKGGCGGGGYYGSGGSGIGGSSQHYINTNRIVDTCQQGQVGQISGGYAGGIGGSYGTGDISQISGSGIGGSRLGGISQISGSGIGGSGLGGISQISGSGISGCR
LGGIRQISGSGIGGSGLGGIRQISGSGIGGSGLGGIRQISGSGIAGIGLGGINQISGIGGSAGGYLNQISGRGGCSSSGGSYGLDCGGIKK

>Xtr-Aedc16
MSGVKGGGGGKRGSFDKGKNTNPCVPDQPYQGSNTAQACTAGPVETFQQGQVGQISGGYTSAGGSYGADFGRDDISQISGSGISQITGSSISGSGGSDVIQISGSGN
SGYGGSDISQISGSGISGYGGSGISQISGSGISGCGGSDISQISGSSIGGSAGSNISQISGSGIGGSAGSNISQISGSGISGCGGSDISQISGSGIGGSAGSNISQI
SGSGISGCGGSDISQISGSGISGSAGSNISQISGSGGCSSQISGSYSSGGSYGSGSGGTKK

>Xtr-Aedc17
MSGVKGGRSHCHQKTYCPDPCQPQVVCKDPCHPQVVCKDPCQPQVVCKDPCQSQVVCKDPCKLTVCHDPCQPTQYFHCQTGHCPQKVHCDPCHPCHPCQCYGKY

>Xtr-Aedc18
MSGVKGGRSHCQKTYCPDPCHPQVVCKDPCHPQVVCKDPCHPQVVCKDPCHPHVVCKDPCQPLVVCKDPCQQTVCYDPCQPTQHLHCQTVHSFQKVYYDPCNPCNPS
QCYGKR

>Xtr-Aedc19
MSGVKGGRSHCHQKTYCPDPCQSQVVCKDPCHPQVVCKDPCHPQVVCKDPCQPLVVCKDPCELAICQDPCQPAQHYHCNTVHCTQKVHCDPCNPCNPCHRYGKY

>Xtr-Aedc20
MSVFKGGRSHCHQKTYCPDPCHPHVVCKDPCNPQVVCNDPCQLAICHDPCQPAHHYHCDTVHCTHKVHCDPCNPCNPCNPWQFYGKY

>Xtr-Aedc21
MSVFKGGRSHCHQKTYCPDPCHPQVVCKDPCHPQVVCNDPCQATICHDPCQPAHHYHCNTVHCTHKVHCDPCNPCNPCHFYGKY

>Xtr-Aedc22
MSGIKGGRSHCHQKTYCPDPCHPHVVCKDPCHPQVVCKDPCQSTICCDPCQPAHHLHCQTVHCTQKVYCDPCNPCQPCQFYGKR

>Xtr-Aedc23
MSGVKGGHQQKSHSQEPCKDSCQKHEGHEQSHKSESHDSHHCHQEASHQQKDHSEQSHHEPPKKH

>Xtr-Aedc24
MSFLKKGLGDIKTDEIVKQGKEAVEESVNQCIQEGQKFVQEVVDKGTEATKDAANKAVTDIKAGFKQK

B

>Xla-Aedc1.L
MPGKADKKKCPDPCPKPKPQKCKDPCKTTPKCPKPAPKCKDPCKPQDCTQDKKCVKKC

```
>Xla-Aedc2.L
MSGVKGQQKKCPDPCPPPPKCQDPCKDPCHKTVPQCQDPCQKTHQKQVHSQDHIQDQNPPHH

>Xla-Aedc3.L
MSGVKGQQKKCQEQTPPQPCNPCPPQPCNPCPPPKCQDPCPKPVPQCPDPCKQHQTHGGQH

>Xla-Aedc4.L
MSAKGGSHQSKDPCVPVQPTPCVEVPTCQDPCVISDPCQTQSHGPGAQVTYSGGCQTQSQGHQQHHGGGSAKK

>Xla-Aedc5.L
MSAKGGQQKQDQCKDPDPCKPVQQCQDTKPQCKDPCATTNPCQKQGQGQGQGHEHHGHHGHHGGQGNHGGGGAKK

>Xla-Aedc6.L
MSGTKGGSGIIGGGGCGVSSGGGSGGTGCGGTGSVSAQCGTTGTNIPIQQCQGTSTVQQTSTGTGTANPCLGQGGNTGGGCLSGGGSGGCGGQTGGGSAKK

>Xla-Aedc7.L
MSGVKGGCGGGGSGCGKSSDQYKNTNPCAPVQQYQGNNIVQGCTGTLNPCQQGQVGQISGSGGANISQISGGCIGGSAGANTSQISGGCTGGSGAGDTSQISGSCIG
GSGGYGTSQISGSGGYGTSQISGSGGYGTSQISGGCSGGSGGGCSSQISGSGGSYGSGCGGVTGGQNGGGGAKK

>Xla-Aedc8.L
MSGVKGGCGGGGSGSGGSSDQYKNTNPCTPVQQYQGNNTVQGCTGTVNPCQQGQVGQITGSGGANISQISGGCIGGSGGANTSQSSGGCTGGSGAIDTSQISGSCIG
GSGGYGTSQISGSGGYGTSQISGSGGYGTSQISGSGGYGTSQISGGCTGGSGGGCSSQISVSGGSYGSGCGGVSGGQNGGGGAKK

>Xla-Aedc9.L
MSGVKGGRSQCQQKTYCPDPCQPQVLCKDPCQPQVVCKDPCQPQVVCKDPCQPQVVCKDPCQHTICHDPCQPAQHFHCQTVHCTQKVHYDPCHSCHPWQFHGKH

>Xla-Aedc10.L
MSGVKGGHSQCQQKFYCPDVCQPQLICKDPCQPHVICKDPCQPQVVCKDPCQPQVVCKDPCQLTIFHDPCQPAQHCHYKTVHCPQKVCHDPCHPCQCYGKH

>Xla-Aedc11.L
MSGVKGGRSQCQQKTYCPDPCQPQVVCKDPCQPQVVCKDPCQPQVLCKDPYQQVVYKDPLQPQFVCKDPCQRTICNDPCQPAQHYHCKTVHCPQKVHCDPCHPCQCY
GKH

>Xla-Aedc12.L
MSGVKGGRSQCQQKTYCPDPCQPQVVCKDPCQPQVICKDPCQPQVICKDPCQLTICHDSCQPAQHYHCQTVHCTQKVHNPCHPCQCYGKH

>Xla-Aedc13.L
MSGVKGGHQQKSHCQEPCKDPCQKHEPCHEPCHKSECHDPCKPAQHHQDPCCQKKEHCEPCHPEPCKKH

>Xla-Aedc14.L
MSFLKKGLGDIKPDGIVKQGKEAVEDTVNQCIQEGQKCVQEVVDKGTEAAKDAANKAVTDIKIGFKQK

>Xla-Aedc1.S
MPGTGDKKKCPDPCPKPKPQKCKDPCKSTPQCPKPTPKCKDPCKPQDCTQDKKCVKKC

>Xla-Aedc2.S
MPGTGDKKKCPDPCPKPKPQKCKDPCKSTPQCPKPTPKCKDPCKPQDCTQDKKCVKKC

>Xla-Aedc3.S
MSGVKGHQTKCPDPCPPPQKCKDPCKDPCPKPVPQCHDPCQKTHQHQGHGQDHSQGHSQGNNQSHH

>Xla-Aedc4.S
MSGVKGQQKKCQEQCPPQPCNPCPPQPCNPCPPPQKCPDTCPKPVPQCQDPCKQQQTHGGQHQH

>Xla-Aedc5.S
MSGVKGHQTKCPDPCPPPQKCKDPCKDPCPKPVPQCQDPCQKTHQHQGHGQDHTQGHNQGNNQPHH

>Xla-Aedc6.S
MSGVKGQQKKCQEQCPPQPCNPCPPQPCNPCPPPQKCQDPCPKPVPQCQDPCKPHQTHGGQHQH

>Xla-Aedc7.S
MSGVKGHQTKCPDPCPPPQKCKDHCKDPCPKPVPQCHDPCQKTHQHQGHGQGHSQGHSQGNNQPHH

>Xla-Aedc8.S
MSGVKGQQKKCQEQCPPQPCNPCPPQPCNPCPPPQKCQDPCPKPVPQCQDPCKPHQTHGGQHQH

>Xla-Aedc9.S
MSGVKGQQKKCQEPAPPQPCNPCPPQPCNPCPPPQKCPDPCPKPVPQCQDPCKPHQTHGGQHQH

>Xla-Aedc10.S
MSGVKGQQKKCQDQAPPQPCNPCPPQPCNPCPPPQKCPDPCPKPVPQCQDPCKQKQTHGGQHQH

>Xla-Aedc11.S
MSAKGGSQCKQDQCKDPDPCIPVQKCQQAKPICKDPCQTTNPCQQQGQGHEHQGHHGGGGGGGGGAAKK

>Xla-Aedc12.S
MSGTKGGSGIIGGGGCGSSSGGGSGGSGTISAQCGTTGTNIPIQQCQGTSTVQQTNPCQTTNPCQQQSQGHEHHGHHGHHGGQGSHGGGGAKK

>Xla-Aedc14.S
MSGTKGGSGIIGGGGCGSSSGGGSGGYGCVGSGIISAQCGDTGTNIPIQQCQGTSTVQQTSTDPCQTTNPCQQQGQGHEHHGHHGHHGHHGGQGSHGGGGAKK

>Xla-Aedc15.S
MSGVKGGGGTYYGGGGSGSGGSSDQCKDTNPCVPVQQYQGNNAGQGSTGTVNPCQQGQVGQISGGYTSGAGGTYGSSGITQISGSGGGNISQLSGGCIGGSGGSDTS
QISGSGGYGTSQISGGCLSGSGGGNTSQISGSGGYGTSQISGGCIGGSGGGNTSQISGSCIGGSGGYGTRQISGSGSGGSGCTSQITGIYSSGVSYGSGCGGSSGGQ
NGGGGAKK

>Xla-Aedc16.S
```

MSGVKGGRSQCQQKTYCPDPCQPQIFCKDPCQKQVVCKDPCQPQVVCKDPCQPQVVCKDPCQPQFVCKDPCQLTMCHDTCQPVQHYHCKTVHCPQKVHCDPCHPCQC
YGKH

>Xla-Aedc17.S
MSGVNGGRSQCQQKTYCPDPCQLQVVCKDPCQPQVVYKDPCQPQVVYKDPCQPQVVCKDPCQLTICHDPCQPAQHYHCQTVHCTQKVHYDPCHPCQCYGKH

>Xla-Aedc18.S
MSFLKKGLGDVKTDEIVKQGKEAVEDTINQCIQEGQQYVQEVVDKGTEAAKDAANKAVTDFKTGFKQK

C

>Bbu-Aedc1
MSGVKGHQKKTPEPCAPPPQQHQDPCKPEPQCEEPIQQVCEPEPVCEQQCPPPQEICTQSSQKKDNIGVKK

>Bbu-Aedc2
MSGVKGQQKKCPEPCPPPKECQDPCKPKPVTPEPRCPSPQTHKPEPSHPEPKCPHPQEQTKPAPRCHEPQDKKCVKK

>Bbu-Aedc3
MSGVKGQQKKCPEPCPPPQQDCQDQCEPVQVCPEPKCPPQVVQCPPPQCQNQQDKQYVKK

>Bbu-Aedc4
MSGVKGQQKKCPEPCPPPQQDCQDQCEPVQVCPEPKCPPQVVQCPPPQCQNQQDKQYVKK

>Bbu-Aedc5
MSGVKGQKKCPEPCPPPQKCQDPCKQVCPEPKCPPPQVCVPVPVCPEPKCPPPQVQCKPVPQCQQQHGK

>Bbu-Aedc6
MSAKGQGKNPCCPPPVCQDQTPKCIIVDPCAPCPKVQCPPPPCCPDPCQQGGYGQGYGGQGGYGYGGNMK

>Bbu-Aedc7
MSAKGQGKNPCCPPPVCQDQTPKCIIVDPCAPCPKVQCPPPPCCPNPCQQGGYGQGYGGQGGYGYGGNMK

>Bbu-Aedc8
MSAKGQGKNPCCPPPVCQDQTPKCIIVDPCAPCPKVQCPPPPCCPDPCQQGGYGQGYGGQGGYGYGGNMK

>Bbu-Aedc9
MSAKGQGKNPCCPPPVCQDQTPKCIIVDPCAPCPKVQCPPPPCCPNPCQQGGYGQGYGGQGGYGYGGNMK

>Bbu-Aedc10
MATKGGKCNPCAQVPQYIQDPCKEPCPPVNPCQVQGQAVGGMGGISGQVSQGGYGGPGGFGGQISQGGYDSAGGFGGQVSQGGYDSAGGFGGQVSQGGYDSAGGFGG
QVSQGGYGGPGGFSGQVSQGGYGGAGGIGGQVSQGGYGGAGGFSGQVSQGGYGGAGGLGGQVSQGGYGEARGFGGVKK

>Bbu-Aedc11
MSDIKCKSGGGYQKGSSGDDCGGGYQKGGSGSGSSGGGCQSSGSGDGCKLGSSGGGFQQGSSGGDCSGGFQQGGSGGGYQKGSSGSDCGGAYQQGGSGGGYQQGSSG
GDCGGGYQQGGSGGGYQQGSSGGDCGGGYGKQNMTLCGDDQQKGGSCTSGGSGQQCQDQGSYGSGSKSGGGAGWGQDSCDGSKSDASQGKGTGACKK

>Bbu-Aedc12
MSGVKGKSGGGNQKGSSGDDCGGGYQKGGSGSGSSGGGCQSSGSGDGCKLGSSGGGFQQGSSGGDCSGGFQQGGSGGGYQKGSSGSDCGGGYQQGGSGGGYQQGSSG
GDCGGGYQQGGSGGGYQQGSSGGDCGGGYQKDASGGDCGGGYGKQNMTLCGDDQQKGGSCTSGGSGQQCQDQGSYGSGSKSGGGAGWGQDSCDGSKSDASQGKGTGA
CKK

>Bbu-Aedc13
MSGIKGGRCGQKIQCQNPCKDPCQKVPICVNPCQDPCKFEIQNHGSHHNHHGQHHFHKQDPCNPCCPDPCQDQGSYSFN

>Bbu-Aedc14
MSGVKGGHQKAQCKDPCQKQTICQDPCQKQTICQDPCQKQTFYQDPCQKQTICQDPCQKQTFYQDPCQKQTICQDSCQDPCKPIQQCQGYQKDPCNPCVPDPCQSQG
KYY

>Bbu-Aedc15
MSGIKGGRCGQKIQCQNPCKDPCQKVPICVNPCQDTCKFEIQNHGSHHNHHGQHHFHKQDPCNPCCPNPCQDQGSYSFNK

>Bbu-Aedc16
MSGVKGGHQKAQCKDPCQKQTICQDPCQKQTICQDPCQKQTFYQDPCQKQTICQDPCQKQTICQDPCQKQTFYQDPCQKQTICQDSCQDPCKPIQQCQGYQKDPCNP
CVPDPCQSQGKYY

>Bbu-Aedc18
MSGVKGGHQKAQCKDPCQKQTICQDPCQKQTICQDPCQKQTFYQDPCQKQTICQDSCQDPCKPIQQCQGYQKDPCNPCVPDPCQSQGKY

>Bbu-Aedc19
MSGVKGGHQKAQCKDPCQKQTICQDPCQKQTICQDPCQKQTFYQDPCQKQTICQDSCQDPCKTIQQCQGYQKDPCNPCVPDPCQSQGKY

>Bbu-Aedc20
MSGVKGGHQKTQCKDPCQKQTICQDPCQKQTISQDPCQKQTIYQDPCQKQTICQDSCQDPCKPIQQCQGYQKDPCNPCVPDPCQSQGKYY

>Bbu-Aedc21
MSGIKGKPGFKQPNPVQPILEPQPICEDIAQVTPQPPAPCPVPVTPQPCAPVQVTPQPPAPVPVAPQPCVPVQVTPQPPAPVPVAPQPCAPEPTVNQEKKGCKK

>Bbu-Aedc22
MSGVKGGHQKAQCKDPCQKQTICQDPCQKQTICQDPCQKQTICQDPCQKQTICQDPCQKQTICQDPCQKQTICQDPCQKQTICQDPCQDPCKPVQQCKDPCNPCVPD
PCQSQGKY

>Bbu-Aedc23
MSGIKGKPGFKQPNPVQPILEPQPICEDIAQVTPKHPAPCPVPMTTQPCAPVQVTPQPPAPVPVTPQPCAPVQVTPQPPAPVPVTPQPCAPVQVTPQPPAPVPVTPQ
PCAPVQVTPQPPAPVPVAPQPCAPVQVTPQPPAPVPVAPQPCVPVQVTPQPPAPVPVAPQPCAPEPTVNQGKKGCKK

>Bbu-Aedc24
MSFLNKGVNLNTSNLVQQGKEAAQDSANQCVQEGQKIAQEVVDKGCQVAKDAGGKAVDQLKGGIKNK

>Bbu-Aedc25
MSSFLNQAQQKADETVKQGEQTVQDCVDKGKEVGQQVIDQGCQTAKNTCDQVTGQVGDKLTGGWKKQS

D

>Bbo-Aedc1
MSGVKGHQKQCKDPCDPCPDPCPPPQKCPDPCKPDPCYKPTPQCQDPCKPSPQQCQQDKNKY

>Bbo-Aedc2
MSGVKGHQKQCPDPCHDPCPPPQKCQDPCKQDPCPKPVPQCQDPCKPEPQQCQQDTKCVKKC

>Bbo-Aedc3
MSGVKGQQQKCHEPCPPKPCPPPQQCLPPQQCLPPQPCPPPQQCPPPQQCLPPQQCLPPQQCLPPQQCPPPQQCLPPQQCPPPQQCLPPQQCLPPQQCSPPQQCLPP
QPCPPPQQCQDPCKPVCPEPVQKCPPVQEPCKPQCPQDQQKCVKK

>Bbo-Aedc4
MSGVKGQQQKCHEPCPPKPCPPPQQCPPPQQCLPPQQCPPPQQCLPPQQCSPPQQCLPPAEMPTPQPCPPPQQCQDPCKPVCPEPVQKCPPVQEPCKPQCPQDQQKC
VKK

>Bbo-Aedc5
MSGVKGQQKKCPEPCPPSHALHHHSSARILANKSQSVLNLFKNALLLKNNANPSAHKTSRNEQKMDLNTWQILLSI

>Bbo-Aedc6
MSGVKGQQKKCPEPCPPQPCPPPPQQCQDPCKQEPKCPEPVQKCPPPQEQCKPQCPQDQQK

>Bbo-Aedc7
MSGTKGGHQKVDKCKDPCVPDVQCQDPCKVVPTPQCQDPCKVVPTPQCQDPCAPDPCQKQGHGGHGGGGGGGGHGGHGGGTKK

>Bbo-Aedc8
MSGTKGGGYSQQQVNKCTDPCAPVQKCVDPCQVQATVQCKVPDPCAPVDPCQQQGGGYQSQGHGSHGSGGYQSQGHGSHGSGGYQSQSQGAGYGGSCKK

>Bbo-Aedc9
MSGTKGGGYSQQQVNKCTDPCAPVQTCVDPCQVQATVQCKVPDPCAPVDPCQQQGGGYQSQGHGSHGSGGYQSQGHGSHGSGGYQSQGHGSHGSGGYQSQGHGSHGS
GGYQSQSQGGGYGGSCKK

>Bbo-Aedc10
MSASKGGSHQQSKGHDHAEDHVKEAHQDKGHGGQEKGHEAQEKGHGGQDKGHEAQEKGHGGQDKCHQGHSGGDGSHDKKGHDDSSKGHGGQDKGHGGDKGGHDEKGH
GGHGGDNKVHKGPAKK

>Bbo-Aedc11
MPAAKGGGQQKHDQCKDPCQQQGHGSQGQGGHGHGGQDHGGQGHCDQGHGGQDHGGQGHGGQGHGGQGHCDQSQGGQGHGGHGGQGHCDQGHGGQSHGEQGHGGKGH
GDQSHGGQGHGGQGHGSQGHGGQSHGGQGHGGQGGQSHGGQSHGGQGHGGQGLGGQGHGGQGGHC

>Bbo-Aedc12
MSGVKGGCGGGGGGGGGGGGGGGYKANPCVTVDPCQNVNISTQKGVVDPCQQSGGGQGSYGSGGGFGGQVSYGGGSQGGQVSQCTGVVDPCQQSGGGQGSYGSGGGF
GGQVSYGGGSQSGQVSQCTGVVDPCQQSGGGQGSYGSGGGFGGQVSYGGGSQGGQDAFRYLGIWISSDPSAWYDLNVLPVLKKVMEAFKNWQKLPLSLSGPKAQNST
GTSPCRTVVTIIMSGVKGGCGGGGGGGGGGGGGYKANLDPCQNVNISTQKCAGVVDPCQQSGGGQGGYGSGGGFGGQVSYGGGSQGGQVSQCTGVVDPCQQSGGGQG
GYGSGGGFGGQVSYGGGSQGGQVSQSGQASHGGGCAKK

>Bbo-Aedc13
MSGVKGGHGQQSQQCQKGQFGQESQQCHQGLQSISQGKDLCQQQQKGVCQDAFGAGQQCQEQSSFGSKSAFGAGQQCQEQSSCGSKSAFGAGQQCQEQSSCGSKSAF
GAGQQCQEQSSCGSKSAFGAGQQCQEQSSCGSKSAFGDGQQCQEKSSCGSKSDACQSQAKAQGVKK

>Bbo-Aedc14
MSGVKGGHCHQKTQCQDPCKDPCQKQSVCQDPCQKQGACHDPCHPVQHCHDQSPCGKKKDPCNPCCPDPCQSQGKNH

>Bbo-Aedc15
MSGVKGGHCHQKTQCQDPCKDPCQKQSVCQDPCQKQSACHDPCHPVQHCHDQSPCGKKKDPCNPCCPDPCQSQGKHH

>Bbo-Aedc16
MSGVKGGHCHQKTQCQDPCKDPCQKQSVCQDPCQKQGACHDPCHPVQHCHDQSPCGKKKDPCNPCCPDPCQSQGKHH

>Bbo-Aedc17
MSGVKGGHCHQKTQCQDPCKDPCQKQSACQDPCQKQSACHDPCHPVQHCHDQSPCGKKKDPCNPCCPDPCQSQGKHH

>Bbo-Aedc18
MSGVKGGHCHQKTQCQDPCKDPCQKQNVCQDPCQKQSACHDPCQPVQHCHDQSPCGKKKDPCNPCCPDPCQSQGKHH

>Bbo-Aedc19
MSGVKGGHWHQKTQCQDPCKDPCQKQNVCHDPCQKQSACHDPCHPVQHCHDQSPCGKKKDPCNPCCPDPCQSQGKHH

>Bbo-Aedc20
MSGVKGGHCHQKTQCQDPCKDPCQKQSACHDPCQKQSACQDPCHPVQHCHEQNPCGKKKDPCNPCCPDPCQSQGKHH

>Bbo-Aedc21
MSGVKGGHCHQKTQCQDPCKDPCQKQSVCQDPCQKQSACHDPCKPVQHCQNQSTCVQKKDPCHPDPCQSQAKHC

>Bbo-Aedc22
MSGVKGGHCHQKTQCQDPCQKQSVCQDPCQKQSVCQDPCQKQSVCQDPCQKQSVCQDPCKPAQHCQAQSAGGQQKDQCHQDPCQSQGKHC

>Bbo-Aedc23
MSGVKGGPHKQPAQQTTVQPAPQPTPQPTPQPTCVPTPAPQPTPQPTCQSVPTPAPQPTPQPTCVPTPAPQPTPQPTCVPTPAPQPTPQPTCVPTPAPQPTPQTTCV
PTPAPQPAPQPTPQSTCDSSKPAPVPHQPCQPAPHPAPQPAPQPQLNPVPVVTPAPTPVQPPKGQTKTGVKK

>Bbo-Aedc24
MSGVKGGQSQCHQNVQCHDPCKDPCQKHDPCKDLCNDPCQKHDPCKDSCHPVQQHCQDHSQKCYKQDPCDPCQTQTHCCKK

>Bbo-Aedc25

MSAFKGISDLQNQAQQKTQEAANQGQQVIQDCVDKGKEVGQQVIDQGCDAAQKTCDQVSGQVKEKIPGGWRK

E

>Ame-Aedc1
MSAKGGCKEPQQPQCQEPQVQGKCQEPCEPVQQCQDQTPQCVPEPCPPPCQDQKSVKGVKKC

>Ame-Aedc2
MSAKGGCKEPQQPQCQEPQVQGKCQEPCKPVQQCQDQTPQCVPEPCPPPCQDQQSVKGVKKC

>Ame-Aedc3
MSAKGGCKEHQQPHCQEPQVQGKCQEPCKPVQQCQDQTPQCIPEPCPPPCQDQQSVKGAKKC

>Ame-Aedc4
MSAKGGCKEPQQPHCQEPQVQGKCQEPCKPVQQCQDQTPQCIPEPCPPPCQDQQSVKGAKKC

>Ame-Aedc5
MSAKGGCKEPQQPHCQEPQVQGKCQEPCKPVQQCQDQTPQCIPEPCPPPCQDQQSVKGAKKC

>Ame-Aedc6
MSAKGGCKEPQQPQCQETQVQAKCQEPCKPVRQCQDQTAQCIPEPCPPPCQDQQSVKGVKKC

>Ame-Aedc7
MSAKGGCKEPQQPHCQEPQVQGKCQEPCKPVQQCQDQTPQCIPEPCPPPCQDQQSVKGVKKC

>Ame-Aedc8
MSAKGGCKEPQQPHCQEPQVQGKCQEPCKPVQQCQDQTPQCIPEPCPPPCQDQQSVKGVKKC

>Ame-Aedc9
MSAKGGCKEPQQPHCQEPQVQGKCQEPCKPVQQCQDQTPQCIPEPCPPPCQDQQSVKGVKKC

>Ame-Aedc10
MSAKGGCKEPQQPHCQEPQVQGKCQEPCEPVQQCQDQTPKCIPEPCPPPCQDQQSVKGVKKC

>Ame-Aedc11
MSAKGGCKEPQQPHCQEPQVQGKCQEPCEPVQQCQDQTPQCVPEPCPPPCQDQQSVKGVKKC

>Ame-Aedc12
MSGFKGGGLGGGGGGGKGCGGGGYYGSGGKGYNGGGYGGGGYGGSGYYGSGGKGYNGGGYGGGGYGGSRGKGYGGGGYGLGGYGSGSFGGGGFGGGNQKWQGQGACQ
GGNTNPCGGFLDCILDPCESFSPCSGQGQQWGGCQK

>Ame-Aedc13
MSAKGGGHQKTQCNEPCPPAPCQQVDQCQDQQPQICPEPCNPDPCQGAQKSQGNLQAGGGGGIGGGYGGSGGGSSKKC

>Ame-Aedc14
MSAKGGGGGGHHHHHHQKTQCEEPQNPCQDPCAPVQQCQDSRKGSQSVDPCNPCPPNPCQDQQGVGQYGGGRK

>Ame-Aedc15
MSLPKGFKGLPKQAETVAQDAVGQGEQVAKDCLEHGKAVGQEVIDKGCKAAKDSGEHVAGQIKGQIPGLKKK

F

>Pwa-Aedc1
MSAKGGYRETQKTQCQDTKVVVKCQDPCAPVVVCQDPVPQCEPQCEPQCEPVPCAPVCQDQHSVKGSRKC

>Pwa-Aedc2
MSAKGGHRETQKTQCQDTKVVVKCQEPCAPVVVCQDPVPQCEPQCEPQCEPAPCAPVCQDQQSVKGGRKC
>Pwa-Aedc3p
MSAKGGHRETQETQCQDAKVVVKCQEPCAPVVVPGPSPSM

>Pwa-Aedc4
MSAKGGGHSQKTQVQESCAQGPCKTVDPCQGQQYGGKGQDACMPDPCQGAQTQQQQQQSGASKKY

>Pwa-Aedc5
MSAKGGGYSQKTQVQESCAQGPCKTVDPCQDQQYGGKGQDACMPDPCQGAQTQQQQQQSGGSKKY

>Pwa-Aedc6
MSAKGGGYSQKTQVQESCAQGPCKTVDPCQGQQYGGKGQDACMPDPCQGAQTQQQQQQSGASKKY

>Pwa-Aedc7
MSAKGGGHHHQKTQCNDPCQQTNPCQDPCAPVQQCQDNSQKCAPVDPCNPCIPDPCQDQQRPGHYIGGKK

>Pwa-Aedc8
MSAKGGGHHHQKTQCNDPCQQTNPCQDPCAPVPQCQDNTQKCAPVDPCNPCPPNPCQDQNQGQYYGSKKC

>Pwa-Aedc9
MSAKGGGHHHQKPQCNDPCQQTNPCQDPCAPVPQCQDNTQKCAPGGSLQPMSA

>Pwa-Aedc10
MSAKGGGHHHQKPQCNDPCQQTNPCQDPCAPVPQCQDNTQKCAPVDPCNPCPPNPCQDQNRGQYYGSKKC

>Pwa-Aedc11
MSAKGGGHHHQKTQCNDPCQQTNPCQDPCAPVPQCQDNTQKCAPVDPCNPCPPDPCQDQNRGQYYGSKKC

>Pwa-Aedc12
MSAKGGGHHQQQKQKPQCPEPCPPEPCQEPCEPVQQCQDQAVQQCQDQAPKCPEPVQPWNPCPPEPCQDQQKPAQKGGCKK

>Pwa-Aedc13

MSAKGGGHHQQHHQKPQCPEPCPPEPCQEPCKPVQQCQDEVQKCPTPVEPCKPSPPVHCQEPQKPAHQGGCKK

>Pwa-Aedc14
MSYQGHHHHQGSYCQDPCDKQHQPCLDPCRPGQRHGHDHGYKCDRPDPCYPSPPHHHAHGKFTNNSAGCKK

>Pwa-Aedc15
MSHQGHHHQGPHHHQGSHCQDPCDKQHQPCHDPCKQGHDHCHDHGHKNDKPDPCHPCPPHQHDHGKCTHGGGGCKK

>Pwa-Aedc16
MSFAKGCKGLTKQAQNLTDEAVAQGEQVAKDCMEQGKAAGQEAIDKGCKAAKESGEHVAGQVKDQFAGMKKK

G

>Rbi-Aedc1
MSAKGGGYQKQQCQDPCQKIPNPCQDPCKDIPQCQDQTSKCQDPCDPYPSQCQQGQGKGYKKC

>Rbi-Aedc2
MSAKGGGGYQKQQCQDDPCQKIPDPCLKTSDPCLKISDPCLKISDPCQNIQDPCQKIADPCQKIADPCQKLADPCLDPCKTVSYCQEKTSICQDPCDPVPTQCQEKH
CKGYTKNC

>Rbi-Aedc3
MSAKGGGHHKQQCQDPCQKISDPCQTSCKDVPQCQDQRPKCPDPCDPSPPQCHQDQGKGYTKKC

>Rbi-Aedc4
MSAKGGGGQWNQKTTSQQDQDNNQVKAFDQTSTIGKGGDGGGQWSQTTGSQSQGQTTSGGKWSQTTGSQSQGQTTTSGKGGDCGGQWSQTTGSQSTGQTTSGGQWSQ
TTGSQSQGQTTTSTTSTTSGKSGDCGGQWSQTTGSQSYGQTTTSSTSTTSGKGGDCGGQWSQTTGSQSYGQTTTSGKGGDCGGQSIGGSQVGGDSSQQKGQSYSQTS
ITSKAGSDGGQCGQQSHGSHDDHKKGHGGGHHTEHHKQGQSGTKKQY

>Rbi-Aedc5
MSAKGGGQWNQKSKGQQDQDDDHSKGHHEHTSSSGKGGDCGGQWSQTTGSSQVSVDSSQQKSQGQTTTSGKGGDCGGQWSQTTGSQSQGQTTTSGKGGDSGGQWSQT
TGSQSIGQTTTSGKGGDSGGQWSQTTGSQSQGQTTTSGKGGDCGGQMSQTTGGSQVGGQSYSQTSITSKAGSDGGQCGQQSHHSHEEHSHKKSQGGGQCGDQQKQSQ
TGSKKQY

>Rbi-Aedc6
MSAKGGGGQKSKCQQDQDDDHSKGHHEQRSPTGKGSDCGGQWSQTTGSQSQGQRSPTGKGSDCGGQWSQSTGGQGQSQTTTSGKGGDCGGQWSQTTGSQSHSQTTTS
GKGGDCGGQLSQTTGSQSQGQTTTSGKGGDCGGQWSQTTGGSQVGSDSCQKGQSQGQTNVSSKGGDDGGHCGQQSHEDHSHKKGQSGGQCGDQQKQSQSGSKKLY

>Rbi-Aedc7
MSAKGGGQCNQNTKCQQDQGNDPCQVKAFDQTTTSGKGGDCGGQWSQTTGSQSHSQTTTSGKGGDCGGQSTGSQSYSQTTTSGKGGNCGGQWSQTTGGSQAGSDSCQ
QKGQSYGQTSTSGKGSGCGGQLNQSTGGSQVGADSCQQKGQNYSQTNTSSKSGNDGGQCGQQSQCGQQSQGSQGAGNQCQQKGQGGAQYTEQKQGQCGSKKQY

>Rbi-Aedc8
MSAKGGGQCNQKTTCQQDQGNDPCQVKAFDQTSTSGKVSNCGGQWNQNTGGSQVGVDSCQQKGQSYGQTSTSGKGSSCGGQWSQSTGGSQVGVDSCQQKGQSYGQTS
TSGKGSGCGGQSYQSTGGSQVGVDSCQQKGQGYSQTSTSGKGSACGGQSYQSTGGSQVGGDSCQQKGHGYGQSSISSKGGNDGGQCGQQSQCGQQSQCSQGVGNQCQ
QKGQSGNQYTDQQKQGQCGSKKQY

>Rbi-Aedc9
MSAKGGRQCNQKTTCQQDQGNDSCQVKAFDQTSTSGKGSGCGGQWSQSTGGSQVVIDSCQQKDQSYGQTSTSGKGSACGGQWNQSTGGSQIGVDSCQQKGQGYSQTS
TSGKGSACGGQWNQSTGGSQIGVDSCQQKGQGYSQTSTSGKGSACGGQWNQSTGGSQVGVDSCQQKGQGYSQTSTSGKGSGCGGQSYQSTGGSQVGVDSCQQKGQAY
GQSSISSKGGNDGGQCGQQSQCGQQSQCSQGVGNQCQQKGQSGNQYTEQQKQGQCGSKKQY

>Rbi-Aedc10
MSAKGGGQCNQMTKCQQDQGNDPCQGKIFDQTSTSGKGSGCGGQSYQSTGGSQVDLNSCQQKAQTYGQTSTSGKGSGCGGQSYQSTGGSQVGADSCQQKGQGYGQTS
NSGKGSGCGGQLNQSTGGSQVGLNSCQQKETSTSGKGSGCGGQWNQTTGGSQVGVDSCQQKGQGYGQTSTSGKGNGCGGQLNQSTGGSQVGVASCQQKGQSCGQTNT
SSKGGNDGGQCGQQSQCGQQSQCSQGVGNQCQQKGQGGGQYTEQQKQGQCGSKKQY

>Rbi-Aedc11
MSAKGGGQCNQMTKCQQGQGNDPCQGKIFDQTSTSGKGSVCGGQSYQSTGGSQGGMNSCQQKGQTYGQTSTSGKGSGCGGQSYQSTGGSQVGVVSCQQKGQGYGQTS
TSGKGSGCGGQLNQSTGGSQVGLNSCQQKGQTYGQTSTSGKGSGCGGQLNQSTGGSQVGVASCQQKGQSCGQTNTSSKGGNDGGQCGQQSQCGQQSQCGQQSQCSQG
VGNQCQQKGKGGGQYTEQQKQGQCGSKKQY

>Rbi-Aedc13
MSAKGGGNWNQKKKSQQDQGNDPQQVKVFDQTSTSGKSSGCGGQLSQSTGGSQSSGDSYQQKDQSYGQTSTSGKSSGCGGQLSQSTGSSQGGGDSYQQKDQSYGQTS
TSGKNSGCGGQVSQSTGGSQGGRDSYQQKDQSYGQTSTSGKSSGCGGQVSQSTGGSQGGRDSYQQKDQSYSETSTSGKSSGCGGQLSQSTGGSQGGGDSYQQKDQSY
GQTSTSGKGSDFGGQWKQSTGGSQGGGDSYQQKVQSSGQTSTSGKSSGCGGQLNQSTGGSQSSGDSYQQKDQSYGQTSTSGKSSGCGGQMSQSTGGSQGGRDSYQQK
DQSYVQTSTSGKSSGCGGQVSQSTGGSQGGGDSYQQKDQSYVQTSTSGKSSSCGGQWNQSTGGSQGGGDSYQQKDQSYGQTSTSGKGSDFGGQWKQSTGGSQGGGDS
YQQKVQSSGQTSTSGKSSGGQLNQSTGGSQSSGDSYQQKDQSYGQTSTSGKSTGCGGQWNQSTGGSQGGGDSYQQKDKSYGQTSTSGKSSGCGGQLSQSTGGSQGGG
DSYQQKDQSYGQTSTSGKGSDFGGQWKQSTGGSQGGGDSYQQKVQSSGQTSTSGKSSGCGGQLSQSTGSSQGGGDSYQQKDQSYAQTSTSGKSSGCGGQLNQSTGGS
QGGGDSYQQKDQSYGQTSTSGKSSDFGGQWKQSTGGSQVVVDSYQQKVQSSGQTSTSGKSSGCGGQLSQSTGSSQGGGDSYQQKDQSYGRGGQYTDQQKQGQYGSKK
QY

>Rbi-Aedc14
MSGKGGRRQRPKKKDCQQNPGDNPCNVEFEVLDQTEISDKNVAAAAAAAAAAAGKSNQKSQCSQNPVDSPYKKSSQTQDQMDVIAKGDNDGGQCNQKSQCSQRAAGD
PCKKSNKKQSG

>Rbi-Aedc15
MAHRGNSWTNNYSNRSNHHFNSNRPFSNYQPNLYFGLTSPQPQPYYHHHHHHPNRPSNSYVYYSSPVVEKPNNQTCCQDNSKGNKK

>Rbi-Aedc16
MFGCGRQRQCYQNRPLCQDLCYRQDPCAIQDPCNDPCKPTYYNQRQSNQYVYYRNPGQCYQSYCQDRGKKCKK

>Rbi-Aedc17
MSGQGHSGHCQGHKPHCQDLSDKQDEGPYPYKPDHHHPLQQHDQHGPHPKPDPYHPSHGHDHVKECKK

>Rbi-Aedc18
MSAKGGYSQCQQQSQCSDPCQNIVIPGQCQDPCVPVQQWQCQDQTPQCPKQVSCQPCPSQCQDQVKAYPGGCKK

>Rbi-Aedc19

MSAKGGHGQFQQQSQCSQPCQQIPDQCQDPCAPVQQCQDQTPECLKSVPCQSYQSQCQEQVKPYPGGCKK

>Rbi-Aedc20
MSAKGGHSHCQQQSQFSEPCQQIPNQCQDSCVPIQQCQDQTPQCSKPDPCQPCPSQCQNQVKPFPGGCKK

>Rbi-Aedc21
MAGLKKGFGNFKHQAESQVQEAASQGEQIVQDCIDKGKQYGQQVVDEACHSMQEGCEQGAEKVKSHIPGLKK

H

>Gse-Aedc1
MSAKGGGYQKQRCQDPCQDPCQDPCKDPCQKIADPCKDPCQKIADPCKDPCQQIADPCRDPCQKIADPCKDPCQKIADPCKDSCKDVPLCQDQISKCQNPCDPCDPI
QTPCQQEKGYKKY

>Gse-Aedc2
MSSKGSGYQKQPCQDPCQDPSQQIGDPGKDSLKGVSQCQDQTSKCQNPCDQVSSQSQQDQNKGYQKC

>Gse-Aedc3
MSAKGQYTKQACQDPCQKTSVPCQQPCQNVSQCQDQSSSCQDPCAPPQNPCQNQGQGKGYKKC

>Gse-Aedc4
MSSSKGGWKQSQQDESYEKITSGSHGGSQGGSQGDDCGGKSWNQGSGKVGVDASYQQGSSHTTTTTTSSSSSSSGKGGDCGGDQSWTSQGSQVTVGDAGQKSSHHSG
QTITTSTTSSGGKGGDCGGSSQVITDQEYSQTTSSSKGSQDQGQCGQKSQHEHHSHHTHHEESHQKGHQGGNQKQCTSGGKK

>Gse-Aedc5
MSSKGGSGQWNQQEQGNSSCQNKGQSYEQTSTSTSTQGSGCGGQWSQSSQSSQGGAGSCQQNTQSSGKTSTTVQSSGCGGQGSQSSQSSQSSQSGGGSCQQNTQITG
QTTTTSQGSDCGGKWSQSSNSSQISQGGGSSYQQGGQSSGQTTTTSQGSDCGGKWSQSSNSSQISQGGGSSYQQGGQSSGQTTTTSQGSDCGGKWSQSSNSSQISQG
GGSSCQQGGQSSGQTTTTSQGSDCGGKWSQSSNSSQISQGGGSSYQQGGQSSGQTTTTSQGSDCGGKWSQSSNSSQISQGGGSSCQQGGQSSGQTTTTSQGSDCGGK
WSQSSNSSQISQGGGSSCQQGGQSSGQISTSGHGSGSGGQWSQSSQSSQSTVSGSCQDKGQSYTQTRSSSKSGNDGGQCGQTSQGGSYESSQNKGQGGCQSTGGSKK

>Gse-Aedc6
MSAKGGRGSSQCNQQTTYQQNQGNISCQNISQGYDQISTTGQGSGCGTQWGQSSQSSQVAVDSCQQKGQVSVQIGTGQGSGCGNQWSQNSQSSQVVQVQQGQVSGQT
STTIQGSGCGGQWNQSSQAGVNSCQQKGQVSGQTSTTIQGSGCGGQWNQSSQAGVNSCQQKGQVSGQITTTNQSSGCGGQQSQSSQSSQSGIGSCQQINQASTQIGM
GSNCGGSWNQQTTQSSQNLVSNFYQNNGQGANQSMGGYDGGQCGQTSQYSQSGAYDSFQNKGQGGCQNTAQQKQWIGGAKK

>Gse-Aedc7
MSAKGGRGSSQCNQQTTYQQNQGNISCQNKNQDYEQTSTTGQGNACGGQWGQSSQSSQVVVVVDSCQQKGQVSGQIGTTGQGSGCGNQWSQNSQSSQVVVNDSCQQG
QVSGQASTTIQSSGCGGQWSQSSQSSQVGVNSCQQRGQVSQQIITTGQSSGCGGQQSQSSQSSQVGVGSCQQKDQVSANIGKGSDCGGSWNQQTTQSSQNLVNNSCQ
NNGQGSNRGSQYTDQQKQCTGAAKK

>Gse-Aedc8
MSAKGNSGSGQCSQQTSSQQNQGNNSCQTKGQSYNQTSTTGQGSGCGGQGSQSSHSSQVTVNNSCQQNSQVSGKTSTAIQGSGCGGQGSQISQNSQGQQRGQGSVQT
STTSQVSGCGGQGSQSSQSSQVVAGSCQQKGQVSGQTSTSSQSSSCGQGSQSSQSSQVVVNNSCQQKGQVSEQTSCGGQQSQSSQVSVTNSCQINSQASGQTSTTSQ
GSGCGGQQSQSSQSSQGVVNNSCQQRSQVSGQSSSSSKGDNHGGQCGQTSQYSQGGAYNSSQNKGQGECQNTDQQKQYTGGVKK

>Gse-Aedc9
MSAKGGNGAGLCNQQTSSQQNQGNNSCQNIGQGYNQTSTSSQDNACGGQRSQSSQNSQVVVNNSCPQKGQVSGQTSTIGQVSSCGGQGNQSSQSSQVIAGSCQQKSQ
VIGQISTSGQGSGCGGQQSQSSQSSQVVVNNSSQQKGQMSGQSSTFVQSSGCGGQQNQSSQISQVSVNNFCQKNNQVSGQTSTSVQGSGCGGQWSQSSQSSQVVAGS
CQQKGQLSGQTSISVQGGNCGGQSSQSSQSSQVGVGSCQQNSQVSGRGCQNTDQQKQCTGGSKK

>Gse-Aedc10
MSAKGGNGAGQCNQQTSSQQNQGNNSCQNIGQGYNQTSTSSQGNACGGQMSQSSQNSQVVVNNSCQQKGQVSGQTSTFGQGSSCGGQGNQSSQSSQVIAGSCQQKGQ
VSGQISTSGQGSGCGGQQSQSSQSSQVVVNNSSQQKGQMSGQSSTFVQSSGCGGQQNQSSQISQVSVNNFCQKNNQVSGQTSTSVQGSGCGGQWSQSSQSSQVVAGS
CQQKGQLSGQTSTSVQGSSCGGQSSQSSQSSQVGVGSCQQNSQVSGRGCQNTDQQKQCTGGSKK

>Gse-Aedc11
MSAKGGNGAGQCNQQSSSQQNQGNNSCQNKGQDYNQTSSTSQNNACGGQGSQSSQSSQVVVNNSSQQKGQASGQTSTTSQVSGCGGQGSQSSQSSQVVTGSSQQKGQ
VSGQTSSSGQGSGCGGQQSQSSQSSQVVVNNSSQQKGQVSEQTSTFSQGSSCGQGSQSSQSSQVVAGSSQQKGQVSGQTSFGQGSSCGGQESQSSQSSQVVVNNSSQ
QKGQVSEQTSTSVQGSGCGGQKSQSSQSSQVVVNNSSQQKGQVSGQTSTSGQGSGCGGQQSQSAQSSQVVVNNSSQQKGQVSEQSSTSVHGSGCGGQQSQSSQSSQV
SVNNSCQKNKVSEQTSTSVQGSSCVGQSSQSSQSSQVGVGSSQQNNQVSGRGCQNPDQQKQCTGGSKK

>Gse-Aedc12p
MNSCQQKGQTSSQTTSTSQGTGCGNQLSQSSQSSQVSVNSCQQRNEASGQTSSTGQSSSCGNQ

>Gse-Aedc13
MSAKGGSRSSQCNQQTTQQEQGNTSCQNKSQGYDHTSTTGKGSACGGQSSGQTSSTSQSTGCGSQWTQSSQSSQGSSQTSSTGQSSSCGNQRSQSSQNQVSGQSSSS
SSGSYAGGQCGQTSQYSQSGANNSSQNKGQGGCQNTSQQNQCTGGSKK

>Gse-Aedc14
MSAKGGGRSSSQWNQQTTYQQDQGNSSCQNTSQGYDQTSSIGQTSDHGNQLSQSSQSSQVLIDSSKQINQGHGQISSTGQSSGSGNQWGQSSQSSQVAIDSSQQSSG
CGNQWGQSSQSSQVAVDSSQQNYQTSSQISSTGQSSGSGNQWGQSSQSSQVAIDSSQQSSGCGNQWGQSSQSSQVAVDSSQQNYQTSSQISSTGQNSGSGNQWGQSS
QSSQVAIDSSQQSSGCGNQWGQSSQSSQVAIDSSQQNYQTSSQISSTGQNSGSGNQWGQSSQSSQVAIDSSQQSSGCGNQWGQSSQSSQVAIDSSQQNYQTSSQISS
TGQNSGSGNQWGQSSQSSQVAIDSSQQNYQTSSQISSTGQGTGCGNQGGYSSQIQVSDQTFISGQGGASGNQWNQSSQSSQIAVDNLMQQKGPVYGQAFITGQGGAS
GNQWNQSSQSSQSAVNNLMQQKGPVYGQTFTSGQGGASGNQWNQSSQNSQISVDSSMQQKGSGFGQSSSFSQGGYDGGQYGQTSQYSQSGAQNFGQGGYQNTSQQNQ
FIGGQKK

>Gse-Aedc15
MATKGRRRQTAPKAECHQEPGGAPCTIETSVSDHDDISDYNAEVSLMSIHNSTISFKHDDVQNEPDDALRNLGHTRDCGNNGRQYEYNQQSQDSQVSVENQCRQSGH
SQNRGNNGRQYEYNQQSQDSQVSVENQCRQSGHSQNQTDVIYKGGSDEGQCYMQSQCSKAPIENTCTQPSQRQNLSTSGSVSTCQNQSSSSPARSGVQYGDVQKQCP
GTRKN

>Gse-Aedc16
MSFWGRQSQCCPPSPDCCPTQDPCARQNPCYNPCKPAQCYPQNNQYTYYRGIGSWSQNKGCQSPGKYCKK

>Gse-Aedc17
MSDHGHPGHCQEHKPHDHHHKHDMGKHSCKPDHHHPEHEHHHDHHAHHPNAAPCDPHHPSQSHSHVKEHKK

>Gse-Aedc18

MASLKKGLGNIQQQAKSEVQDAACQGEQVVHDCIDKGKQVGQQVLDQASHAVQEACEEVTGQVKSHMPSVHKK

Figure S2. Amino acid sequences of AEDC proteins. Species: (**A**) Western clawed frog (*Xenopus tropicalis,* Xtr), (**B**) African clawed frog (*Xenopus laevis,* Xla), (**C**) common toad (*Bufo bufo*, Bbu), (**D**) fire-bellied toad (*Bombina bombina*, Bbo), (**E**) axolotl (*Ambystoma mexicanum,* Ame), (**F**) Iberian ribbed newt (*Pleurodeles waltl*, Pwa), (**G**) two-lined caecilian (*Rhinatrema bivittatum,* Rbi), (**H**) Gaboon caecilian (*Geotrypetes seraphini*, Gse).

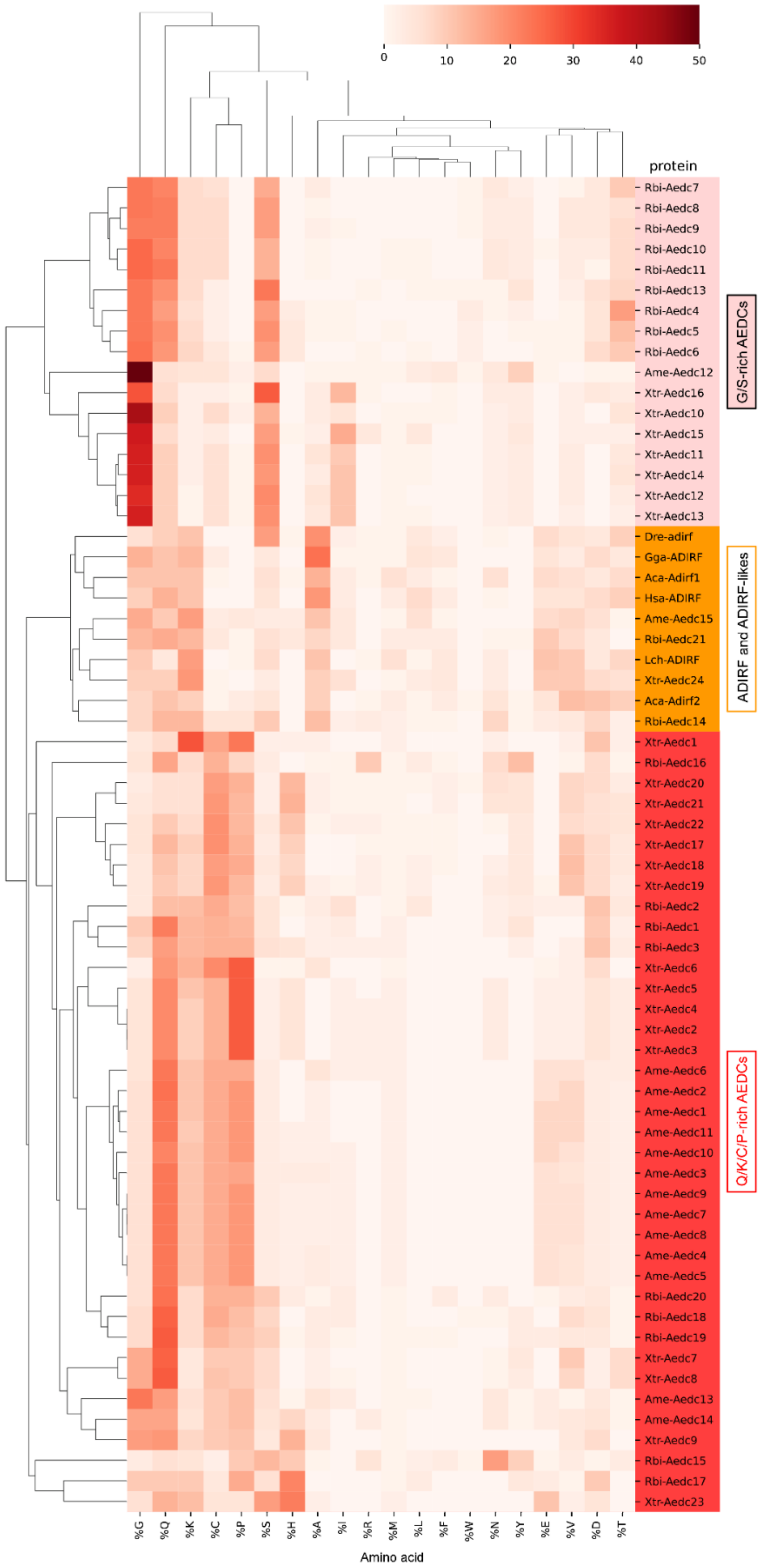

Figure S3. Hierarchical clustering of the AEDC proteins and amniote ADIRF according to their amino acid composition. Similarities of the AEDC proteins are indicated by hierarchical clustering. The amino acid composition was used to compute the clustermap. The three main protein groups are indicated on the right: ; G/S-rich AEDC proteins, ADIRF and ADIRF-like AEDC proteins and Q/K/C/P-rich AEDC proteins. The one-letter code for amino acids is used. Species: human (*Homo sapiens,* Hsa), chicken (*Gallus gallus,* Gga), anole lizard (*Anolis carolinensis,* Aca), western clawed frog (*Xenopus tropicalis,* Xtr), african clawed frog (*Xenopus laevis,* Xla), axolotl (*Ambystoma mexicanum,* Ame), two-lined caecilian (*Rhinatrema bivittatum,* Rbi), coelacanth (*Latimeria chalumnae*, Lch), zebrafish (*Danio rerio*, Dre).

```
Hsa-ADIRF      MAS--KGLQ--DLKQQVEGTAQEA------------VSAAGAAAQQVVDQATEAGQKAMDQLAKTTQETIDKTANQAS
Gga-ADIRF      MLG--RGFQ--GLKEQAEGAAKDA------------ASALGQATQDAVSQITDAGQKAVDKACKTAQDGAEKAAGQAA
Aca-ADIRF1     MSG--KNIQ--GFAQQTERTMHDA------------ANSLGKATQKGMNQVSEAGQKAVDQTCKMVQEGVDKATGEAA
Aca-ADIRF2     MF-KWDPVE--GATKMAGDTVQSK------------VDSAGQTVQQVVDQVTDAGQKAYDDVCKNVQDTGEKATRSLN
Amphibians
Bbu-Aedc24     MSFLNKGV---NLN--TSNLVQQG------------KEAAQDSANQCVQEGQKIAQEVVDKGCQVAKDAGGKAVDQLK
Bbu-Aedc25     MSSF---------LNQAQQKADET------------VKQGEQTVQDCVDKGKEVGQQVIDQGCQTAKNTCDQVTGQVG
Bbo-Aedc25     MSAF-KGIS--DLQNQAQQKTQEA------------ANQGQQVIQDCVDKGKEVGQQVIDQGCDAAQKTCDQVSGQVK
Xtr-Aedc24     MSFLKKGLG--DIK--TDEIVKQG------------KEAVEESVNQCIQEGQKFVQEVVDKGTEATKDAANKAVTDIK
Xla-Aedc14.L   MSFLKKGLG--DIK--PDGIVKQG------------KEAVEDTVNQCIQEGQKCVQEVVDKGTEAAKDAANKAVTDIK
Xla-Aedc18.S   MSFLKKGLG--DVK--TDEIVKQG------------KEAVEDTINQCIQEGQQYVQEVVDKGTEAAKDAANKAVTDFK
Ame-Aedc15     MS-LPKGFK--GLPKQAETVAQDA------------VGQGEQVAKDCLEHGKAVGQEVIDKGCKAAKDSGEHVAGQIK
Pwa-Aedc16     MS-FAKGCK--GLTKQAQNLTDEA------------VAQGEQVAKDCMEQGKAAGQEAIDKGCKAAKESGEHVAGQVK
Gse-Aedc18     MASLKKGLG--NIQQQAKSEVQDA------------ACQGEQVVHDCIDKGKQVGQQVLDQASHAVQEACEEVTGQVK
Rbi-Aedc21     MAGLKKGFG--NFKHQAESQVQEA------------ASQGEQIVQDCIDKGKQYGQQVVDEACHSMQEGCEQGAEKVK
Lch-ADIRF      MD-FDKGSNQTSLASKAKETTQNV------------SEKTGGVVKDMAEKAKEMGKQKVEEVVEGVARAAEKAT---P
Dre-ADIRF      MASFKKSLE--GLKNSSQSATKGVTDSA--------VQAAQDAVQQVAESSKEATSTAAEEASRQTQSAIGTAAGKAS
Loc-ADIRF      MASVKKSLE--GLKGTTQAAAKGVTDSA--------VQAAQEAVQQVAESSKETTKMAADEASKQTQSAIGTAAGKAT
Cpu-ADIRF      MTSMKKSFE--ELKNTTQTAAKGMADTTVKLFPHLLVQAAQEAVQQVAESSKETTKTATEEASKQTQTAINSVASKAQ
Hak-ADIRF      MAAIKTSIE--ELKSRTQTAAKGMS-----------VEAAQEAVQQVAESSKDTTKTATEEASKQTQTAIDSAASKAQ
Lre-ADIRF      MDSINKSLG--GLAGQAKTTGEGIVQSV--------QEGAQQAVDQVVDAANKTADTAAKEASKQAQLAADAGLREGE

Hsa-ADIRF      DTFSGIGKKFGLLK---------------------------------------------------------------
Gga-ADIRF      EALSGFGKKCGFKK---------------------------------------------------------------
Aca-ADIRF1     NAISGLEKK--------------------------------------------------------------------
Aca-ADIRF2     PFGQNEEKNAST-----------------------------------------------------------------
Amphibians
Bbu-Aedc24     ---GGIKNK--------------------------------------------------------------------
Bbu-Aedc25     DKLTGGWKKQS------------------------------------------------------------------
Bbo-Aedc25     EKIPGGWRK--------------------------------------------------------------------
Xtr-Aedc24     ---AGFKQK--------------------------------------------------------------------
Xla-Aedc14.L   ---IGFKQK--------------------------------------------------------------------
Xla-Aedc18.S   ---TGFKQK--------------------------------------------------------------------
Ame-Aedc15     GQIPGLKKK--------------------------------------------------------------------
Pwa-Aedc16     DQFAGMKKK--------------------------------------------------------------------
Gse-Aedc18     SHMPSVHKK--------------------------------------------------------------------
Rbi-Aedc21     SHIPGL-KK--------------------------------------------------------------------
Lch-ADIRF      GGFSKFMTK--------------------------------------------------------------------
Dre-ADIRF      DTIKEFGHKLSSK----------------------------------------------------------------
Loc-ADIRF      DAIKEFGQQLGPK----------------------------------------------------------------
Cpu-ADIRF      DSIKGFGQRLMQK----------------------------------------------------------------
Hak-ADIRF      DSIKVLGQRLVQK----------------------------------------------------------------
Lre-ADIRF      GCHGGLQQELRPVSHSCRKEEPHSALLRPPPPPPPPPPLALVEDCVQQFPACVSTSPLTPLLHFALLMKSSARLSLK
```

Figure S4. Amino acid sequence alignment of ADIRFs and ADIRF-like AEDC proteins. The amino acid sequences of ADIRF proteins and ADIRF-like AEDC proteins were aligned. Glutamine (Q) and lysine (K) residues as potential sites of transglutamintation are highlighted gray and blue shading, respectively. Cysteine (yellow shading), proline (green shading) and glycine (red fonts) are also highlighted. The sequences are sorted according to the phylogenetic distance of the respective species to humans. Species: Human (*Homo sapiens,* Hsa), chicken (*Gallus gallus,* Gga), anole lizard (*Anolis carolinensis,* Aca), western clawed frog (*X. tropicalis,* Xtr), African clawed frog (*X. laevis,* Xla), axolotl (*Ambystoma mexicanum,* Ame), Iberian ribbed newt (*Pleurodeles waltl*, Pwa), two-lined caecilian (*R. bivittatum,* Rbi), African lungfish (*Protopterus annectens,* Pan), coelacanth (*Latimeria chalumnae,* Lch), zebrafish (*Danio rerio,* Dre), spotted gar (*Lepisosteus oculatus,* Loc), brownbanded bamboo shark (*Chiloscyllium punctatum,* Cpu), red stingray (*Hemitrygon akajei,* Hak), and Far Eastern brook lamprey (*Lethenteron reissneri,* Lre).

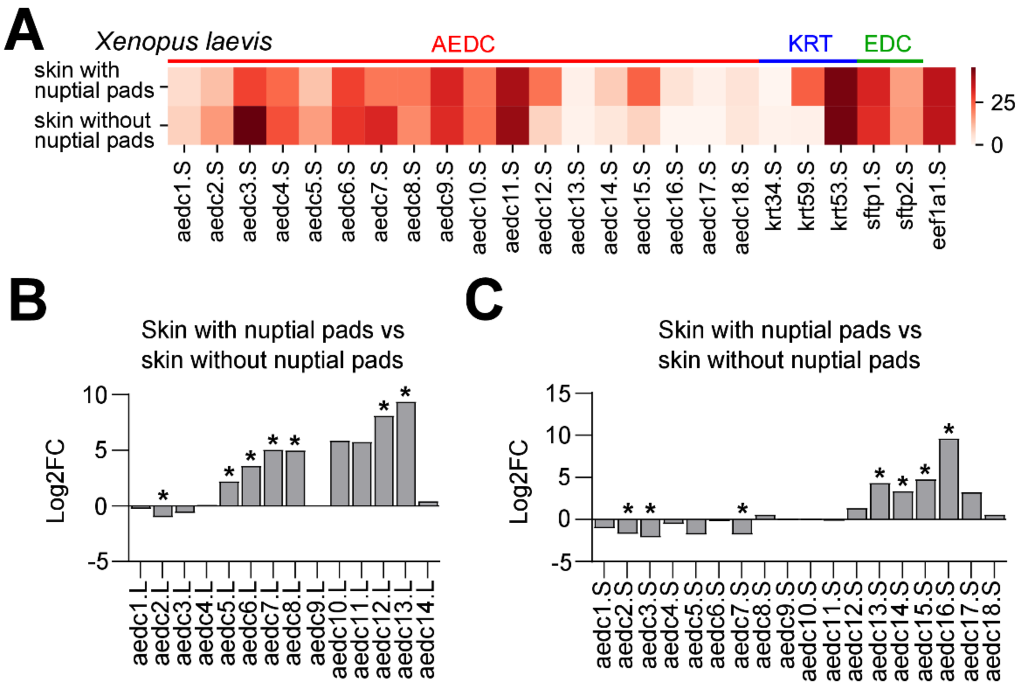

Figure S5. Expression of AEDC genes of chromosome 7.S of *X. laevis* in skin with and without nuptial pads. (A) Expression levels of the AEDC genes of chromosome 7.S of *X. laevis* (Tables S6 and S7) were determined by the analysis of RNA-seq data. The heatmap shows the cube root-transformed mean of read counts of three biological replicates. (B, C) Comparison of expression levels in skin with nuptial pads versus skin without nuptial pads. AEDC genes of chromosome 7.L (B) and chromosome 7.S (C) were compared (Table S7) Log2 fold changes (FC) are shown in bar charts (**B**, **C**). *, adjusted P-value <0.05.

Cornified layer (SC):

```
Aedc2              MSGVKGQQKKCPEPCPPQPCNPCPPPQKCQDPCKDPCPKPIPQCQDPCQRTPTQQQGQNQPHHH
Frog 1 peptide 1                                      PCPKPIPQCQDPCQRTPTQQQGQNQPHHH
Frog 1 peptide 2                                                 PCQRTPTQQQGQNQPHHH
Frog 2 peptide 1                                                 PCQRTPTQQQGQNQPHHH

Aedc9              MSAKGSQCKDPDPCHPVVQCQDPKPQCKDPCAPVDPCQQQGQGHGQGHQGHHHHHHQHGGQSGQGCGGAKK
Frog 1 peptide 1   MSAKGSQCKDPDPCHPVVQCQD
Frog 1 peptide 2   MSAKGSQCKDPD
Frog 1 peptide 3   MSAKGSQCKD
Frog 1 peptide 4             PDPCHPVVQCQD
Frog 2 peptide 1   MSAKGSQCKDPDPCHPVVQCQD
Frog 2 peptide 2   MSAKGSQCKDPD
Frog 2 peptide 3   MSAKGSQCKD
Frog 2 peptide 4               PCHPVVQCQD
```

Clawless toe:

```
Aedc9              MSAKGSQCKDPDPCHPVVQCQDPKPQCKDPCAPVDPCQQQGQGHGQGHQGHHHHHHQHGGQSGQGCGGAKK
peptide 1          MSAKGSQCKD
```

Claw:

```
Aedc23             MSGVKGGHQQKSHSQEPCKDSCQKHEGHEQSHKSESHDSHHCHQEASHQQKDHSEQSHHEPPKKH
peptide 1                                             SHDSHHCHQE
```

Figure S6. Detection of Aedc proteins by mass spectrometry-based proteomics. The cornified layer (stratum corneum, SC) was isolated from the epidermis of trunk skin of two *X. tropicalis* frogs. In addition, clawless toes and cornified claws were prepared. The samples were digested with GluC protease, which cleaves on the carboxy-terminal side of aspartic acid (D) and glutamic acid (E) residues. The sequences of peptides corresponding to AEDC proteins are aligned to the complete protein sequences of Aedc2, Aedc9 and Aedc23. D and E are highlighted by red fonts.

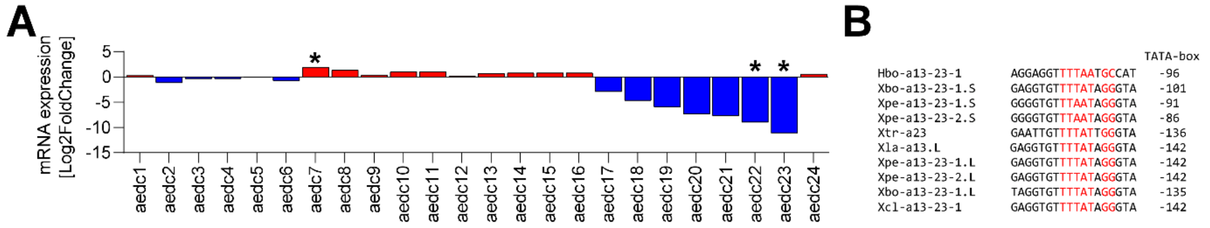

Figure S7. The expression of *Xenopus tropicalis aedc23* depends on the transcription factor Hoxc13. (**A**) mRNA levels of the aedc genes in the toes of wildtype and Hoxc13-deficient *Xenopus tropicalis* frogs (Carron et al. 2024). Bars represent log2-fold changes upon loss of Hoxc13. Asterisks indicate significant differences (adjusted p-value < 0.05). (**B**) Conserved Hoxc13 binding sites in the promoter of the *aedc* (a) genes homologous to Xla *aedc13.L* and Xtr *aedc23* in pipid frogs (a13-23). Red fonts indicate the core motif. Numbers indicate the position of the last nucleotide relative to the TATA box. Species: Hbo, *Hymenochirus boettgeri* (Zaire dwarf clawed frog); Xbo, *Xenopus borealis* (Marsabit clawed frog); Xcl, *Xenopus clivii* (Eritrea clawed frog); Xla, *Xenopus laevis* (African clawed frog); Xpe, *Xenopus petersii* (Peters' platanna); Xtr, *Xenopus tropicalis* (tropical clawed frog).

>Rbi-Aedc21
AGLKKGF-AESQVQE---DKDEACH-------------KVK
>Xla-Aedc18.S
SFLKKGLGGKEAVED---QEDKGTE-------------DFK
>Xla-Aedc14.L
SFLKKGLGGKEAVED---QEDKGTE-------------DIK
>Xtr-Aedc24
SFLKKGLGGKEAVEE---QEDKGTE-------------DIK
>Dre-adirf
ASFKKSL-SQSATKG---QQEEASR-------------KAS
>Lch-ADIRF
MDFDKGSNAKETTQN---EKEEVVE----------------
>Hsa-ADIRF
MASK-GL-VEGTAQE---DQDQLAK-------------QAS
>Gga-ADIRF
MLGR-GF-AEGAAKD---SQDKACK-------------QAA
>Aca-Adirf1
MSGK-NI-TERTMHD---NQDQTCK-------------EAA
>Aca-Adirf2
MFKWDPV-AGDTVQS---DQDDVCK-------------SLN
>Bbo-Aedc1
MSGVKG--HQKQCKDPPPQKPDPCKPQCDPCKPSQQCQQDK
>Bbo-Aedc2
MSGVKG--HQKQCPDPPPQKQDPCKPQCDPCKPEQQCQQDT
>Bbo-Aedc3
MSGVKG--QQQKCHEPPPQQQDPCKQKCEPCKP--QCPQDQ
>Bbo-Aedc4
MSGVKG--QQQKCHEPPPQQQDPCKQKCEPCKP--QCPQDQ
>Bbo-Aedc5
MSGVKG--QQKKCPE----------SVLNNANPS----AHK
>Bbo-Aedc6
MSGVKG--QQKKCPEPPPQQQDPCKQKCEQCKP--QCPQDQ
>Bbo-Aedc14
MSGVKGGHQKTQCQDDPCQKHDPCHPCGDPCNPCDPCQQGK
>Bbo-Aedc15
MSGVKGGHQKTQCQDDPCQKHDPCHPCGDPCNPCDPCQQGK
>Bbo-Aedc16
MSGVKGGHQKTQCQDDPCQKHDPCHPCGDPCNPCDPCQQGK
>Bbo-Aedc17
MSGVKGGHQKTQCQDDPCQKHDPCHPCGDPCNPCDPCQQGK
>Bbo-Aedc18
MSGVKGGHQKTQCQDDPCQKHDPCQPCGDPCNPCDPCQQGK
>Bbo-Aedc19
MSGVKGGHQKTQCQDDPCQKHDPCHPCGDPCNPCDPCQQGK
>Bbo-Aedc20
MSGVKGGHQKTQCQDDPCQKQDPCHPCGDPCNPCDPCQQGK
>Bbo-Aedc21
MSGVKGGHQKTQCQDDPCQKHDPCKTCVDPCH--DPCQQAK
>Bbo-Aedc22
MSGVKGGHQKTQCQDDPCQKQDPCKAGGDQCH--DPCQQGK
>Bbo-Aedc23
MSGVKGGPQQTTVQPQPTPQQTTCVSTCQPCQPATPVQQTK
>Bbo-Aedc24
MSGVKGGQQNVQCHDDPCQKKDSCHQKCDPCDPC---QQTH
>Bbo-Aedc25
MSAFKGI-AQQKTQE---DKDQGCD-------------QVK
>Gse-Aedc1
MSAK-GGGQKQRCQDDPCQKKDSCKSKCNPCDPCTPCQQEK
>Gse-Aedc2
MSSK-GSGQKQPCQDDPSQQKDSLKSKCNPCDQVSQSQQNK
>Gse-Aedc3
MSAK-G--TKQACQD---QKQQPCQSSCDPCAPPNPCQQGK
>Gse-Aedc15
MATK-GRRPKAECHQYEYNQENQCRGQCSQCSKAQPSQQNQ
>Gse-Aedc16
MSFW-G--RQSQCCPDPCARYNPCKNQYGSWSQNKGCQPGK
>Gse-Aedc17
MSDH-G--HPGHCQEDHHHKKHSCKPNAAPCDPHHPSQHVK
>Gse-Aedc18
ASLKKGL-AKSEVQD---DKDQASH-------------QVK
>Pwa-Aedc1
MSAK-GGYQKTQCQD----KQDPCAPQCVPCAP--VCQHSV
>Pwa-Aedc2
MSAK-GGHQKTQCQD----KQEPCAPQCAPCAP--VCQQSV
>Pwa-Aedc4
MSAK-GGGQKTQVQE-----QGPCKGKGDACMP-DPCQQQQ
>Pwa-Aedc5
MSAK-GGGQKTQVQE-----QGPCKGKGDACMP-DPCQQQQ

```
>Pwa-Aedc6
MSAK-GGGQKTQVQE-----QGPCKGKGDACMP-DPCQQQQ
>Pwa-Aedc7
MSAK-GGGQKTQCND---QQQDPCAQKCDPCNPCDPCQQRP
>Pwa-Aedc8
MSAK-GGGQKTQCND---QQQDPCAQKCDPCNPCNPCQNQ-
>Pwa-Aedc9
MSAK-GGGQKPQCND---QQQDPCAQKCGSLQPM-------
>Pwa-Aedc10
MSAK-GGGQKPQCND---QQQDPCAQKCDPCNPCNPCQNR-
>Pwa-Aedc11
MSAK-GGGQKTQCND---QQQDPCAQKCDPCNPCDPCQNR-
>Pwa-Aedc12
MSAK-GGGQKPQCPE-----QEPCEPKCQPWNPCEPCQQKP
>Pwa-Aedc13
MSAK-GGGQKPQCPE-----QEPCKQKCEPCKPSVHCQQKP
>Pwa-Aedc14
MSYQ-GHHQGSYCQD---DKLDPCRYKCDPCYPSPPHHHGK
>Pwa-Aedc15
MSHQ-GHHQGSHCQD---DKHDPCKHKNDPCHPCPPHQHGK
>Pwa-Aedc16
MSFAKGC-AQNLTDE---EQDKGCK-------------QVK
>Rbi-Aedc1
MSAK-GGGQKQQCQD---QKQDPCKSKCDPCDPYSQCQQGK
>Rbi-Aedc2
MSAKGGGGQKQQCQDDPCQKLDPCKSICDPCDPVTQCQHCK
>Rbi-Aedc3
MSAK-GGGHKQQCQD---QKQTSCKPKCDPCDPSPQCHQGK
>Rbi-Aedc14
MSGK-GGRKKKDCQQ---NQDSPYKGQCSQCSQRDPCK---
>Rbi-Aedc15
MAHR-GNSSNHHFNS-----HHPNRNSYKPNNQT--CQNSK
>Rbi-Aedc16
MFGC-G--RQRQCYQDPCAINDPCKNQYGQCYQSY-CQRGK
>Rbi-Aedc17
MSGQ-G--HSGHCQGDLSDKPYPYKPKPDPYHPS----HVK
>Rbi-Aedc18
MSAK-GGYQQSQCSD---QNQDPCVPQCVSCQPCSQCQQVK
>Rbi-Aedc19
MSAK-GGHQQSQCSQ---QQQDPCAPECVPCQSYSQCQQVK
>Rbi-Aedc20
MSAK-GGHQQSQFSE---QQQDSCVPQCDPCQPCSQCQQVK
>Ame-Aedc1
MSAK-GGCQQPQCQE----QQEPCEPQC-PCPP--PCQKSV
>Ame-Aedc2
MSAK-GGCQQPQCQE----QQEPCKPQC-PCPP--PCQQSV
>Ame-Aedc3
MSAK-GGCQQPHCQE----QQEPCKPQC-PCPP--PCQQSV
>Ame-Aedc4
MSAK-GGCQQPHCQE----QQEPCKPQC-PCPP--PCQQSV
>Ame-Aedc5
MSAK-GGCQQPHCQE----QQEPCKPQC-PCPP--PCQQSV
>Ame-Aedc6
MSAK-GGCQQPQCQE----QQEPCKAQC-PCPP--PCQQSV
>Ame-Aedc7
MSAK-GGCQQPHCQE----QQEPCKPQC-PCPP--PCQQSV
>Ame-Aedc8
MSAK-GGCQQPHCQE----QQEPCKPQC-PCPP--PCQQSV
>Ame-Aedc9
MSAK-GGCQQPHCQE----QQEPCKPQC-PCPP--PCQQSV
>Ame-Aedc10
MSAK-GGCQQPHCQE----QQEPCEPKC-PCPP--PCQQSV
>Ame-Aedc11
MSAK-GGCQQPHCQE----QQEPCEPQC-PCPP--PCQQSV
>Ame-Aedc13
MSAK-GGGQKTQCNE-----PAPCQPQIEPCNP-DPCQQGN
>Ame-Aedc14
MSAK-GGGQKTQCEE-----QDPCAKGSDPCNPCNPCQQGV
>Ame-Aedc15
MSLPKGF-AETVAQD---EHDKGCK-------------QIK
>Bbu-Aedc1
MSGVKG--HQKKTPEPPPQQQDPCKPVCQQCPPPEICTQKK
>Bbu-Aedc2
MSGVKG--QQKKCPEPPPKEQDPCKPKCEQTKPAPRCHQDK
>Bbu-Aedc3
MSGVKG--QQKKCPEPPPQQQDQCE-KCVQCPP-PQCQQDK
>Bbu-Aedc4
MSGVKG--QQKKCPEPPPQQQDQCE-KCVQCPP-PQCQQDK
```

>Bbu-Aedc5
MSGVKG---QKKCPEPPPQKQDPCKPKCVQCKPVPQCQHGK
>Bbu-Aedc6
MSAK-GQG------------KNPCCPKCDPCAPCDPCQYGQ
>Bbu-Aedc7
MSAK-GQG------------KNPCCPKCDPCAPCNPCQYGQ
>Bbu-Aedc8
MSAK-GQG------------KNPCCPKCDPCAPCDPCQYGQ
>Bbu-Aedc9
MSAK-GQG------------KNPCCPKCDPCAPCNPCQYGQ
>Bbu-Aedc13
MSGIKGGRQKIQCQNNPCQ--DPCKHHFDPCNPCDPCQQGS
>Bbu-Aedc14
MSGVKGGHQKAQCKDDPCQKQDPCK---DPCNPCDPCQQGK
>Bbu-Aedc15
MSGIKGGRQKIQCQNNPCQ--DTCKHHFDPCNPCNPCQQGS
>Bbu-Aedc16
MSGVKGGHQKAQCKDDPCQKQDPCK---DPCNPCDPCQQGK
>Bbu-Aedc18
MSGVKGGHQKAQCKDDPCQKQDPCK---DPCNPCDPCQQGK
>Bbu-Aedc19
MSGVKGGHQKAQCKDDPCQKQDPCK---DPCNPCDPCQQGK
>Bbu-Aedc20
MSGVKGGHQKTQCKDDPCQKQDPCK---DPCNPCDPCQQGK
>Bbu-Aedc21
MSGIKGKPPQPICED-----PQPCAPVAQPCVPVQPCAQEK
>Bbu-Aedc22
MSGVKGGHQKAQCKDDPCQKQDPCK---DPCNPCDPCQQGK
>Bbu-Aedc23
MSGIKGKPPQPICEDQPCAPPQPCAPVAQPCVPVQPCAQGK
>Bbu-Aedc24
SFLNKGV-GKEAAQD---QEDKGCQ-------------QLK
>Bbu-Aedc25
------M-AQQKADE---DKDQGCQ-------------QVG
>Xla-Aedc1.L
MPG-KA--DKKKCPDPKPQKKDPCKPKCDPCKP-QDCTQDK
>Xla-Aedc2.L
MSGVKG--QQKKCPDPPPPKQDPCKPQCDPCQKTQKQVHSQ
>Xla-Aedc3.L
MSGVKG--QQKKCQEPPP-K-----PQCDPCKQHTHGGQH-
>Xla-Aedc4.L
MSAK-GGS--HQSKDVPVQ-PTPCV---DPCVISDPCQQSQ
>Xla-Aedc5.L
MSAK-GGQKQDQCKD------DPCKPQCDPCATTNPCQQGQ
>Xla-Aedc9.L
MSGVKGGRQKTYCPDDPCQHHDPCQVHCDPCHSCHPWQHGK
>Xla-Aedc10.L
MSGVKGGHQKFYCPDDPCQLHDPCQVHCDPCHPC---QYGK
>Xla-Aedc11.L
MSGVKGGRQKTYCPDDPCQRNDPCQVHCDPCHPC---QYGK
>Xla-Aedc12.L
MSGVKGGRQKTYCPDDPCQLHDSCQVHCNPCHPC---QYGK
>Xla-Aedc13.L
MSGVKGGHQKSHCQEEPCHKHDPCKPCCEHCEPCEPCK---
>Xla-Aedc1.S
MPG-TG--DKKKCPDPKPQKKDPCKPKCDPCKP-QDCTQDK
>Xla-Aedc2.S
MPG-TG--DKKKCPDPKPQKKDPCKPKCDPCKP-QDCTQDK
>Xla-Aedc3.S
MSGVKG--HQTKCPDPPPQKKDPCKPQCDPCQKTQHQGHGQ
>Xla-Aedc4.S
MSGVKG--QQKKCQEPPPQK-----PQCDPCKQQTHGGQHQ
>Xla-Aedc5.S
MSGVKG--HQTKCPDPPPQKKDPCKPQCDPCQKTQHQGHGQ
>Xla-Aedc6.S
MSGVKG--QQKKCQEPPPQK-----PQCDPCKPHTHGGQHQ
>Xla-Aedc7.S
MSGVKG--HQTKCPDPPPQKKDHCKPQCDPCQKTQHQGHGQ
>Xla-Aedc8.S
MSGVKG--QQKKCQEPPPQK-----PQCDPCKPHTHGGQHQ
>Xla-Aedc9.S
MSGVKG--QQKKCQEPPPQK-----PQCDPCKPHTHGGQHQ
>Xla-Aedc10.S
MSGVKG--QQKKCQDPPPQK-----PQCDPCKQKTHGGQHQ
>Xla-Aedc11.S
MSAK-GGSKQDQCKD------DPCIPICDPCQTTNPCQQGQ
>Xla-Aedc16.S
MSGVKGGRQKTYCPDDPCQLHDTCQVHCDPCHPC---QYGK

```
>Xla-Aedc17.S
MSGVNGGRQKTYCPDDPCQLHDPCQVHCDPCHPC---QYGK
>Xtr-Aedc1
MPG-KG--DKKKCPDPKPQKKDPCKPKCDPCKP-QDCTQDK
>Xtr-Aedc2
MSGVKG--QQKKCPEPPPQKQDPCKPQCDPCQRTTQQQQNQ
>Xtr-Aedc3
MSGVKG--QQKKCPEPPPQKQDPCKPQCDPCQRTTQQQQNQ
>Xtr-Aedc4
MSGVKG--QQKKCPEPPPQKQDPCKPQCDPCQRTTQQQQNQ
>Xtr-Aedc5
MSGVKG--QQKKCPEPPPQKQDPCKPQCDPCQKTTQQQQNQ
>Xtr-Aedc6
MSGVKG--QQKKCQEPPPQKCDPCAPQCDPCKQQH------
>Xtr-Aedc7
MSAK-GGS--QQYKDVQVQPVQPCQQPCDPCVQSTQCQQGQ
>Xtr-Aedc8
MSAK-GGS--QQYKDVQVQPVQPCQQPCDPCVQSTQCQQGQ
>Xtr-Aedc9
MSAK-G----SQCKD------DPCHPQCDPCAPVDPCQHGQ
>Xtr-Aedc17
MSGVKGGRQKTYCPDDPCKLHDPCQGHCDPCHPCHPCQYGK
>Xtr-Aedc18
MSGVKGGRQKTYCPDDPCQQYDPCQVHSDPCNPCNPSQYGK
>Xtr-Aedc19
MSGVKGGRQKTYCPDDPCELQDPCQVHCDPCNPCNPCHYGK
>Xtr-Aedc20
MSVFKGGRQKTYCPDDPCQLHDPCQVHCDPCNPCNPCNYGK
>Xtr-Aedc21
MSVFKGGRQKTYCPDDPCQAHDPCQVHCDPCNPCNPCHYGK
>Xtr-Aedc22
MSGIKGGRQKTYCPDDPCQSCDPCQVHCDPCNPCQPCQYGK
>Xtr-Aedc23
MSGVKGGHQKSHSQEEQSHKHD---ASHDHSEQSEPPKH--
```

Figure S8. Amino acid sequence alignment in fast_aln format for phylogenetic analysis. Multiple sequence alignments were generated with MAFFT and automatically trimmed with BMGE. Species: human (*Homo sapiens,* Hsa), chicken (*Gallus gallus,* Gga), anole lizard (*Anolis carolinensis,* Aca), western clawed frog (*Xenopus tropicalis,* Xtr), African clawed frog (*Xenopus laevis,* Xla), common toad (*Bufo bufo*, Bbu), fire-bellied toad (*Bombina bombina*, Bbo), axolotl (*Ambystoma mexicanum,* Ame), iberian ribbed newt (*Pleurodeles waltl*, Pwa), two-lined caecilian (*Rhinatrema bivittatum,* Rbi), Gaboon caecilian (*Geotrypetes seraphini*, Gse), coelacanth (*Latimeria chalumnae*, Lch), and zebrafish (*Danio rerio*, Dre).

A

```
>Xtr-aedc1
ATGCCTGGAAAGGGAGATAAGAAGAAATGTCCAGACCCATGCCCAAAACCAAAGCCCCAGAAGTGCAAAGACCCTTGCAAGAGTCCTCCACAATGCCATAAACCAGC
ACCAAAATGCAAAGATCCCTGCAAGCCCCAAGATTGTACCCAAGACAAAAAATGTGTGAATAAATGA

>Xtr-aedc2
ATGTCTGGAGTTAAGGGTCAGCAAAAGAAATGCCCAGAGCCTTGCCCACCCCAGCCATGCAATCCATGCCCTCCACCACAGAAATGCCAAGATCCTTGCAAGGATCC
TTGCCCTAAGCCAATCCCACAATGCCAAGACCCATGCCAGAGGACTCCGACTCAACAGCAGGGTCAAAACCAACCCCACCATCACTGA

>Xtr-aedc3
ATGTCTGGAGTTAAGGGTCAGCAAAAGAAATGCCCAGAGCCTTGCCCACCCCAGCCATGCAATCCATGCCCTCCACCACAGAAATGCCAAGATCCTTGCAAGGATCC
TTGCCCTAAGCCAATCCCACAATGCCAAGACCCATGCCAGAGGACTCCGACTCAACAGCAGGGCCAAAACCAACCCCACCATCACTGA

>Xtr-aedc4
ATGTCTGGAGTTAAGGGTCAGCAAAAGAAATGCCCAGAGCCTTGCCCACCCCAGCCATGCAATCCATGCCCTCCACCACAGAAATGCCAAGATCCTTGCAAGGATCC
TTGCCCTAAGCCAATCCCACAATGCCAAGACCCATGCCAGAGGACTCCGACTCAACAGCAGGGCCAAAACCAACCCCACCATCACTGA

>Xtr-aedc5
ATGTCTGGAGTTAAGGGTCAGCAAAAGAAATGCCCAGAGCCTTGCCCACCCCAGCCATGCAATCCATGCCCTCCACCACAGAAATGCCAAGATCCTTGCAAGGATCC
TTGCCCTAAGCCAATCCCACAATGCCAAGACCCATGCCAGAAGACTCCGACTCAACAGCAGGGCCAAAACCAACCCCACCATCACTGA

>Xtr-aedc6
ATGTCTGGAGTTAAGGGTCAACAAAAGAAATGCCAAGAGCAATGCCCACCACCCTGTGATCCATGCGCACCAAAGTGCCCTCCACCACAGAAATGTGATCCATGCCC
TCCACCACAGAAATGTGATCCATGCGCACCAAAGTGCCCTCCACCACAGAAATGTGATCCATGCGCACCAAAGTGCTGTCCTCCACAACAGAAATGCCAGGATCCTT
GCGCTAAGCAAGTCCCACAATGCCAAGATCCATGTAAGCAACAGCAACACTAA

>Xtr-aedc7
ATGTCAGCCAAAGGAGGCAGCCAACAATACAAGGACCCATGTGTCCAAGTACAGCCATGCCAGGGTACCCAAGTACAGCCATGCCAGGGTACCCAAGTACAGCCATG
CCAGGGTGTCCAAGTACAGCCATGCCAGCCACAAGTAGATCCTTGTGTGCAAAGTAATCCCTGCCCAACTCAATGTCAGGGGCAGAGCGGGCAAGTCACCTATACCG
GAGGCTGCCAGACACAAGGCCAAGGTTATGGAGGACAATCCTACGGTTCAGCCAAGAAATAAAGAAATCAAAGCTTCATGGCACTGATCTATCTCCTCACATTAAAT
TACAGCTGA

>Xtr-aedc8
ATGTCAGCCAAAGGAGGCAGCCAACAATACAAGGACCCATGCGTCCAAGTACAGCCATGCCAGGGTACCCAAGTACAGCCATGCCAGGGTACCCAAGTACAACCATG
CCAGGGTATCCAAGTACAGCCATGCCAGCCACAAGTAGATCCTTGTGTGCAAAGTAATCCCTGCCCAACTCAATGTCAGGGGCAGAGCGGGCAAGTCACCTATACCG
GAGGCTGCCAGACACAAGGCCAAGGTTATGGACAATCCTGCGGTTCAGCCAAGAAATAA

>Xtr-aedc9
ATGTCTGCTAAAGGAAGCCAATGCAAAGATCCTGACCCCTGCCATCCAGTGGTGCAATGCCAGGATCCCAAGCCCCAGTGCAAAGATCCTTGTGCACCAGTTGATCC
CTGCCAGCAACAAGGGCAAGGGCATGGTCAAGGGCATCAGGGTCACCACCATCATCATCACCAGCATGGAGGACAAAGTGGCCAAGGATGTGGAGGAGCCAAGAAGT
AA

>Xtr-aedc10
ATGTCTGGAGCCAAGGGTGGAAGTGGCATCATCGGTGGTGGGGGTTGTGGTGGCAGCAGTGGCAGCAGTGGCGGATTGGGTTGTGGTGGCAGTGGAGCAATATCTAC
TCAATGTGGAAACACTGGCATAAATATACCAGTTCAACCATGCCAGGGCACTGTCCAGCAAGGAGGCACAGCTAATCCCTGTCTGCAAAGCCAAGGTGGAGGAAGTT
TCATTAGTGGTGGTGGATCAAGCTATGGAGGAGGCTGTGGGGGCCAGATTGGTGGATCAAGCTATGGAGTAGGCTGTGGAGGCCAGACCGGTGGATCCCAGATTAGT
GGATCAAGCTGTGGAGGAGGCTGTGGAGGGCAGACTGGTGGATCAGGTTATGGAGGAGGCTGTGGAGGCCAGACTGGTGGCTCAGGTTATGGAGGAGGCTGTGGAGG
CCAGACTGGTGGAGGTTCAGCCAAAAAGTGA

>Xtr-aedc11
ATGTCTGGAGTCAAAGGTGGTTGTGGTGGAGGGGGTGGATATTATGGCAGTGGAGGCAGTGGAATAGGAGGATCCTCACAACAATACATAAGTACTAACCCATGTGC
ACCAGTTCAGCAACTCCAAGGTAACCAGGGATGCGCAGGCACTGTTACCACTTGCCAACAAGGTCAGGTTGGTCAGATAAGTGGAGGTTGCGCAGGAGGTGCTGGAG
GAACTTATGGAGCAAGTAATATCAGCCAGATTAGTGGAACTGGCATTTGTGGAAGTGGAGCAGGTGGCATCAGCCAGATTAGTGGAAGTGGCATCAGTGGATGCGGA
GCAGGTGGCATCAGCCAGATTAGTGGAAGTGGCATCAGTGGATGCGGAGCAGGTGGCATCGGCCAGATTAGTGGAAGTGGCATTGGTGGAAGCGGAGCAGGTGGCAT
CAGCCAGATTAGCGGAAGTGGCATTTGCGGAAGCGGAGCAGGTGGCATCAGCCAGATTAGTGGAAGCGGAGGCTGCAGCAGCAGCCAGACAAGTGGAAGCTACAATG
TAGGAGGAAGCTATGGATCAGGCTGTGGAGGAGGAGTCAAAAAATGA

>Xtr-aedc12
ATGTCTGGAGTCAAAGGTGGTTGTGGTGGAGGAGGTGGATATTATGGCAGTGGAGGCAGTGGAATAGGAGGATCCTCACAACAATACATAAGTACTAACCCATGTGC
ACCAGTTCAGCAACTCCAAGGTAACCAGGGATGTGCAGGCACTGTTACCACTTGCCAACAAAGTCAGGTTGGCCAGATAAGTGGAGGTTGCGCAGGAGGTGCTGGAG
GAACTTATGGAGCAAGCAATATCAGCCAGATTAGTGGCATCGGTGGATGCGGAGCAGGTGGCATCAGCCAGATTAGTGGAAGTGGCATCAGTGGAAGCGGAGTAGGC
ATGAGCCAGATAAGTGGAAGTGGCATCAGTGGATGCGGAGCAGGTGGCATCAGCCAGATTAGTGGAAGTGGCATCAGTGGATGCGGAGCAGGTGGCATCAGCCAGAT
TAGTGGAAGTGGCATCAGTGGATGCGGAGCAGGTGGCATCAGCCAGATTAGTGGAAGTGGCATTGGTGGAAGTGGAGCAGGTGGCATCAGCCAGATTAGTGGAAGTG
GAGGCTGCTGCAGCAGCCAGACGAGTGGAAGCTACAATGTTGGAAGAAGCTATGGATCAGGCTGTGGAGGAGGAGTTAAAAAATGA

>Xtr-aedc13
ATGTCTGGAGTCAAAGGTGGTTGTGGTGGAGGAGGTGGATATTATGGCAGTGGAGGCAGTGGAATAGGAGGATCCTCACAACAATACATAAGTACTAACCCATGTGC
ACCAGTTCAGCAACTCCAAGGTAACCAGGGATGTGCAGGCACTTGCCAACAAGGTCAGGTTGGCCAGATAAGTGGAGGTTGCGCAGGAGGTGCTGGAGGAACTTATG
GAGCAAGTAATATTAGCCAGATTAGTGGCATCGGTGGAAGCGGACTAGGTGGCATCAACCAGATTAGTGGAAGTGGCATCAGTGGATGCGGAGCAGGCATCAGCCAG
ATTAGTGGAAGTGGCATCAGTGGATGCGGAGCAGGCATCAGCCAGATTAGTGGAAGTGGCATCAGTGGATGCGGAGCAGGTGGCATCAGCCAGATTAGTGGAAGTGG
CATTGGCGGATGTGGAACAGGTGGCATCAGCCAGATTAGTGGAAGTGGCATTTGTGGAAGCGGAGCAGGTGGCATCAGCCAGATTAGTGGAAGCGGAGGCTGCTGCA
GCAGCCAGACGAGTGGAAGCTACAATGTTGGAGGAAGCTATGGATCAGGCTGTGGAGGAGGAGTCAAAAAATGA

>Xtr-aedc14
ATGTCTGGAGTCAAAGGTGGTTGTGGTGGAGGAGGTGGATATTATGGCAGTGGAGGCAGTGGAATAGGAGGATCCTCACAACAATACATAAGTACTAACCCATGTGC
ACCAGTTCAGCAACTCCAAGGTAACCAGGGATGTGCAGGCACTGTTACCACTTGCCAACAAGGTCAGGTTGGCCAGATAAGTGGAGGTTGCACAGGAGGTGCTGGAG
GAACTTATGGAGCAAGTAATATCAGCCAGATTAGTGGCATCGGTGGAAGCGGAGTAGGCATCAGCCAGATTAGTGGAAGTGGCATCAGTGGATGCGGAGCAGGTGGC
ATCAGCCAGATTAGTGGAAGTGGCATCAGTGGATGCGGAGCAGGTGGCATCAGCCAGATTAGTGGAAGTGGCATTGGCGGATGCGGAACAGGTGGCATCAGCCAGAT
TAGTGGAAGTGGCATTGGCGGATGTGGAACAGGTGGCATCAGCCAGATTAGTGGAAGCGGAGGCTGCTGCAGCAGCCAGACGAGTGGAAGCTACAATATTGGAGGAA
GCTATGGCTCAGGCTGTGGAGGAGGAGTCAAAAAATGA

>Xtr-aedc15
ATGTCTGGAGTCAAAGGTGGTTGTGGTGGAGGTGGATATTATGGCAGTGGAGGCAGTGGAATAGGCGGATCCTCACAGCACTACATAAATACTAACCGCATTGTTGA
CACTTGCCAGCAAGGTCAGGTTGGCCAGATAAGTGGAGGTTATGCAGGTGGTATTGGAGGAAGCTATGGAACAGGTGACATCAGCCAGATTAGTGGAAGTGGCATCG
GTGGAAGCAGACTAGGTGGCATCAGCCAGATTAGTGGAAGTGGCATTGGTGGAAGTGGACTAGGTGGCATCAGCCAGATTAGCGGAAGTGGGATCAGCGGATGCAGA
CTAGGTGGCATCAGACAGATTAGCGGAAGTGGCATCGGTGGAAGTGGACTAGGTGGCATCAGACAGATTAGCGGAAGTGGCATTGGTGGAAGTGGACTAGGTGGCAT
```

CAGACAGATTAGCGGAAGTGGCATCGCTGGAATCGGACTAGGTGGCATCAACCAGATTAGTGGCATTGGTGGAAGTGCAGGAGGTTACCTCAACCAGATTAGTGGAA
GAGGAGGCTGTAGCAGTAGTGGAGGAAGCTATGGATTAGACTGTGGAGGAATCAAAAAATGA

>Xtr-aedc16
ATGTCTGGAGTCAAGGGTGGTGGAGGTGGAAAAAGAGGATCATTTGACAAGGGCAAAAATACTAACCCATGTGTACCTGATCAGCCATACCAAGGTAGCAACACTGC
CCAGGCATGCACAGCAGGCCCTGTTGAGACTTTCCAGCAAGGCCAGGTTGGCCAGATAAGTGGCGGTTACACAAGTGCTGGAGGAAGCTATGGAGCAGACTTTGGAA
GAGATGATATAAGCCAGATTAGTGGAAGTGGCATCAGCCAGATTACTGGAAGTAGCATCAGTGGAAGTGGAGGAAGTGATGTCATCCAGATTAGTGGAAGTGGCAAC
AGTGGATATGGAGGAAGTGATATCAGCCAGATAAGTGGAAGTGGCATCAGTGGATATGGAGGAAGTGGCATCAGCCAGATTAGTGGAAGTGGCATCAGTGGATGTGG
AGGAAGTGATATCAGCCAGATTAGTGGAAGCAGCATCGGTGGAAGTGCAGGAAGTAATATCAGCCAGATTAGTGGAAGCGGCATCGGTGGAAGTGCAGGAAGTAATA
TCAGCCAGATTAGTGGAAGTGGCATCAGTGGATGTGGAGGAAGTGATATCAGCCAGATTAGTGGAAGCGGCATCGGTGGAAGTGCAGGAAGTAATATCAGCCAGATT
AGTGGAAGTGGCATCAGTGGATGTGGAGGAAGTGATATCAGCCAGATTAGTGGAAGCGGCATCAGTGGAAGTGCAGGAAGTAATATTAGCCAGATTAGTGGAAGTGG
AGGCTGCAGCAGCCAAATTAGTGGGAGTTACAGCAGTGGAGGAAGCTATGGATCTGGCAGTGGAGGAACGAAAAAATGA

>Xtr-aedc17
ATGTCTGGAGTCAAAGGAGGCCGCAGCCACTGCCATCAGAAGACTTACTGCCCAGATCCATGCCAGCCACAAGTCGTCTGCAAAGATCCATGCCACCCACAAGTTGT
CTGTAAAGATCCTTGCCAGCCGCAAGTTGTCTGTAAAGATCCATGCCAGTCACAAGTTGTCTGCAAAGATCCTTGCAAACTTACAGTATGCCATGACCCCTGCCAAC
CTACCCAGTATTTCCACTGCCAGACTGGGCACTGTCCACAAAAAGTTCACTGTGACCCTTGCCATCCCTGCCATCCTTGCCAATGCTATGGAAAGTACTGA

>Xtr-aedc18
ATGTCTGGAGTCAAGGGAGGCCGCAGCCACTGCCAGAAGACTTACTGCCCAGATCCATGCCATCCACAAGTCGTCTGCAAAGATCCATGCCACCCACAAGTTGTCTG
CAAAGATCCATGCCACCCACAAGTCGTCTGCAAAGATCCATGCCACCCACACGTCGTCTGCAAAGATCCATGCCAACCACTAGTTGTCTGCAAAGATCCTTGCCAAC
AAACCGTATGCTATGACCCCTGCCAACCTACCCAGCATTTGCACTGCCAAACTGTGCACTCTTTTCAAAAAGTGTACTATGACCCATGCAATCCCTGCAATCCTAGC
CAATGCTATGGAAAGCGCTGA

>Xtr-aedc19
ATGTCTGGAGTCAAGGGAGGCCGCAGCCACTGCCATCAGAAGACTTACTGCCCAGATCCATGCCAGTCACAAGTTGTCTGCAAAGATCCTTGCCACCCACAAGTTGT
CTGCAAAGATCCATGCCATCCACAAGTTGTCTGCAAAGATCCATGCCAGCCACTAGTTGTCTGCAAAGATCCTTGTGAACTAGCAATATGCCAGGACCCCTGCCAAC
CTGCCCAGCATTACCATTGCAACACTGTGCACTGTACCCAAAAAGTTCACTGTGACCCTTGCAACCCCTGCAATCCTTGTCATCGCTATGGAAAGTACTGA

>Xtr-aedc20
ATGTCTGTATTCAAAGGAGGCCGCAGCCACTGCCATCAGAAGACTTACTGCCCAGATCCATGCCATCCACATGTTGTCTGCAAAGATCCATGCAACCCACAAGTTGT
CTGCAACGATCCTTGCCAACTAGCAATATGTCATGACCCCTGCCAACCTGCCCATCATTACCATTGTGACACTGTGCACTGTACCCACAAAGTTCACTGTGACCCTT
GCAATCCCTGCAATCCCTGCAATCCTTGGCAATTCTATGGAAAGTACTGA

>Xtr-aedc21
ATGTCTGTATTCAAAGGAGGCCGCAGCCACTGCCATCAGAAGACTTACTGCCCAGATCCATGCCATCCACAAGTCGTCTGCAAAGATCCATGCCACCCACAAGTTGT
TTGTAATGATCCCTGCCAAGCAACAATATGCCATGACCCCTGCCAACCTGCCCATCATTACCATTGCAACACTGTGCACTGTACCCACAAAGTTCACTGTGACCCTT
GCAATCCCTGCAATCCTTGTCATTTCTATGGAAAGTACTGA

>Xtr-aedc22
ATGTCTGGAATCAAAGGAGGCCGCAGCCACTGCCATCAGAAGACTTACTGCCCAGATCCATGCCATCCACATGTTGTCTGCAAAGATCCATGTCACCCACAAGTTGT
CTGCAAAGATCCATGCCAATCAACCATATGCTGTGACCCCTGCCAACCTGCCCATCATTTGCATTGCCAGACTGTGCATTGTACCCAAAAAGTTTACTGTGACCCTT
GCAATCCCTGCCAACCTTGCCAGTTCTATGGAAAGCGCTGA

>Xtr-aedc23
ATGTCTGGAGTTAAAGGAGGACACCAGCAAAAATCACACAGTCAGGAACCCTGCAAGGATTCTTGCCAAAAGCATGAAGGCCATGAACAGAGCCATAAAAGTGAAAG
TCATGATTCTCATCATTGCCACCAGGAGGCGAGTCATCAGCAAAAAGACCATTCTGAGCAAAGTCATCATGAACCCCCTAAAAAGCACTGA

>Xtr-aedc24
ATGTCTTTTTTAAAGAAAGGACTTGGAGATATAAAAACCGATGAGATTGTAAAACAAGGAAAGGAAGCTGTGGAGGAGTCTGTAAATCAGTGCATTCAAGAAGGGCA
AAAATTTGTTCAAGAAGTGGTTGACAAAGGCACTGAAGCTACTAAAGATGCTGCAAACAAGGCAGTGACTGATATTAAAGCTGGCTTCAAGCAGAAGTGA

B

>Xla-aedc1.L
ATGCCTGGAAAAGCAGACAAGAAGAAATGCCCAGACCCATGCCCAAAACCAAAGCCCCAGAAATGCAAAGATCCTTGCAAGACTACTCCAAAATGCCCTAAACCTGC
ACCAAAATGCAAAGATCCCTGCAAGCCACAAGATTGTACCCAAGACAAAAAATGTGTCAAGAAGTGTTAA

>Xla-aedc2.L
ATGTCTGGAGTTAAGGGTCAGCAAAAGAAATGCCCTGATCCATGCCCTCCACCACCAAAATGTCAAGATCCCTGCAAGGATCCTTGCCATAAGACAGTCCCACAGTG
CCAAGACCCATGCCAGAAGACTCACCAAAAGCAGGTCCATAGCCAGGACCATATCCAGGACCAGAACCCACCCCATCACTAA

>Xla-aedc3.L
ATGTCTGGAGTTAAAGGTCAACAAAAGAAATGCCAAGAGCAAACCCCACCACAGCCCTGCAATCCATGCCCTCCACAGCCCTGTAACCCATGCCCTCCACCGAAGTG
TCAGGATCCTTGCCCCAAGCCAGTCCCACAATGCCCAGATCCATGTAAGCAACACCAGACCCATGGAGGTCAACACTGA

>Xla-aedc4.L
ATGTCGGCCAAAGGAGGCAGCCATCAATCAAAGGACCCATGTGTCCCAGTACAGCCCACCCCCTGTGTTGAAGTACCAACATGCCAGGACCCTTGTGTGATTTCTGA
CCCTTGTCAAACACAAAGTCATGGGCCTGGTGCTCAAGTCACCTATAGTGGAGGCTGTCAGACACAAAGCCAAGGTCATCAACAGCATCATGGAGGCGGTTCAGCCA
AGAAATGA

>Xla-aedc5.L
ATGTCCGCTAAAGGTGGCCAACAAAAGCAAGATCAATGCAAAGATCCTGACCCCTGTAAACCAGTGCAGCAATGTCAGGATACCAAGCCCCAATGCAAAGATCCTTG
CGCAACAACTAACCCCTGCCAAAAACAAGGTCAAGGTCAAGGTCAAGGGCATGAGCACCATGGCCATCATGGCCACCACGGAGGCCAAGGCAACCATGGAGGTGGAG
GAGCCAAGAAGTAA

>Xla-aedc6.L
ATGTCTGGAACTAAGGGAGGAAGTGGCATCATTGGTGGTGGCGGCTGTGGTGTGAGCAGTGGTGGAGGTAGTGGTGGAACTGGCTGTGGTGGCACTGGATCAGTATC
TGCCCAATGTGGAACTACTGGCACAAATATACCAATACAACAATGCCAGGGCACAAGCACTGTCCAACAAACAAGCACAGGCACAGGCACAGCTAACCCCTGTCTAG
GCCAAGGTGGTAACACTGGTGGAGGTTGTCTCAGTGGAGGTGGTTCAGGAGGCTGTGGAGGCCAGACTGGTGGAGGTTCAGCCAAAAAGTGA

>Xla-aedc7.L
ATGTCTGGTGTAAAGGGTGGTTGTGGTGGAGGAGGCAGTGGATGTGGCAAATCATCAGACCAATACAAAAATACTAATCCATGTGCACCTGTTCAGCAATACCAGGG
TAACAACATTGTCCAGGGATGCACAGGCACTCTTAATCCTTGCCAGCAAGGTCAGGTTGGCCAGATAAGTGGAAGCGGAGGAGCTAATATCAGCCAAATTAGTGGAG
GATGCATCGGTGGAAGTGCAGGAGCTAATACCAGCCAGATTAGTGGAGGTTGCACTGGTGGAAGTGGAGCAGGTGACACCAGCCAGATCAGTGGCAGTTGCATCGGT
GGAAGTGGAGGATATGGCACCAGCCAGATTAGTGGAAGTGGAGGATATGGCACCAGCCAGATTAGTGGAAGTGGAGGATATGGCACCAGCCAGATTAGTGGAGGTTG
CAGCGGTGGAAGTGGAGGAGGTTGCAGCAGCCAGATAAGTGGGAGTGGTGGAAGCTATGGATCAGGCTGTGGAGGAGTAACTGGTGGCCAGAATGGTGGGGGAGGAG
CCAAAAAGTGA

>Xla-aedc8.L
ATGTCTGGTGTAAAGGGTGGTTGTGGTGGAGGAGGCAGTGGAAGTGGAGGATCATCAGACCAATACAAAAATACTAATCCATGTACACCTGTTCAGCAATATCAGGG
TAACAACACTGTCCAGGGATGCACAGGCACTGTTAATCCTTGTCAGCAAGGTCAGGTTGGCCAGATAACTGGAAGCGGAGGAGCTAATATCAGCCAAATTAGTGGAG
GATGCATCGGTGGAAGTGGAGGAGCTAATACCAGCCAGAGTAGTGGAGGTTGCACTGGTGGAAGTGGAGCAATTGACACCAGCCAGATCAGTGGCAGTTGCATCGGT
GGAAGTGGAGGATATGGCACCAGCCAGATTAGTGGAAGTGGAGGATATGGCACCAGCCAGATTAGTGGAAGTGGAGGATATGGCACCAGCCAGATTAGTGGAAGTGG
AGGATATGGCACCAGCCAGATTAGTGGAAGTGGAGGATATGGCACTAGCCAGATTAGTGGAGGTTGCACCGGTGGAAGTGGAGGAGGTTGCAGCAGCCAGATAAGTG
TGAGTGGTGGAAGCTATGGATCAGGCTGTGGAGGAGTATCTGGTGGCCAGAATGGTGGGGGTGGAGCCAAAAAGTGA

>Xla-aedc9.L
ATGTCTGGAGTCAAAGGAGGCCGCAGCCAGTGCCAACAGAAGACTTACTGCCCAGATCCATGCCAGCCACAAGTCCTCTGCAAAGATCCATGCCAGCCTCAAGTTGT
CTGCAAAGATCCATGCCAACCTCAAGTTGTCTGCAAAGATCCATGCCAACCTCAAGTTGTCTGCAAAGATCCTTGCCAACATACCATATGCCATGACCCCTGCCAAC
CTGCCCAACATTTCCACTGCCAAACTGTGCATTGTACCCAAAAAGTTCACTATGACCCATGCCATTCCTGCCATCCTTGGCAATTCCATGGAAAGCACTGA

>Xla-aedc10.L
ATGTCTGGAGTCAAAGGAGGCCACAGCCAGTGCCAGCAGAAGTTTTATTGCCCTGATGTATGCCAGCCACAACTAATCTGCAAAGATCCATGCCAACCACATGTCAT
CTGCAAAGATCCATGCCAGCCCCAAGTCGTTTGCAAAGATCCATGCCAGCCTCAAGTTGTCTGTAAAGATCCTTGCCAACTAACCATATTCCATGATCCCTGCCAAC
CTGCCCAGCATTGCCACTACAAGACTGTGCACTGTCCCCAAAAAGTTTGCCATGATCCATGCCATCCTTGCCAATGCTATGGAAAGCACTGAGTATTCTCACTTTTT
ATCCAGGACTTGA

>Xla-aedc11.L
ATGTCTGGAGTCAAAGGCGGTCGCAGCCAGTGCCAGCAGAAGACTTACTGCCCAGATCCATGCCAGCCACAAGTTGTCTGCAAAGATCCATGCCAGCCACAAGTTGT
CTGCAAAGATCCATGCCAGCCACAAGTCCTCTGCAAAGATCCATATCAGCAGGTTGTATACAAGGATCCATTGCAACCACAGTTTGTCTGTAAAGATCCTTGTCAAC
GAACCATATGTAATGACCCCTGCCAACCTGCCCAGCATTACCATTGCAAGACTGTGCACTGCCCCCAAAAAGTTCACTGTGACCCTTGCCATCCTTGCCAGTGCTAT
GGAAAGCACTGA

>Xla-aedc12.L
ATGTCTGGAGTCAAAGGAGGCCGCAGCCAGTGCCAGCAGAAGACTTACTGCCCAGATCCATGTCAGCCCCAAGTCGTCTGCAAAGATCCATGTCAGCCCCAAGTTAT
CTGCAAAGATCCATGCCAGCCTCAAGTCATCTGCAAAGATCCATGCCAACTAACCATATGCCATGACTCCTGCCAACCTGCCCAGCATTACCATTGCCAGACTGTGC
ATTGTACCCAAAAAGTTCACAACCCTTGCCATCCTTGCCAATGTTATGGAAAGCACTGA

>Xla-aedc13.L
ATGTCTGGAGTTAAAGGAGGACACCAGCAAAAATCCCACTGTCAGGAACCCTGCAAGGATCCTTGCCAAAAGCATGAACCCTGCCATGAACCATGCCATAAAAGTGA
ATGCCATGACCCCTGCAAACCAGCTCAGCACCACCAGGACCCATGCTGCCAGAAAAAAGAGCACTGTGAGCCATGTCATCCTGAGCCCTGTAAAAAGCACTGA

>Xla-aedc14.L
ATGTCTTTTTTAAAGAAAGGACTTGGAGATATTAAACCCGATGGGATTGTAAAACAAGGAAAGGAAGCTGTGGAGGATACTGTAAACCAGTGCATTCAAGAAGGGCA
AAAATGTGTTCAAGAAGTGGTTGACAAAGGCACAGAAGCTGCTAAAGATGCTGCAAACAAGGCAGTAACTGATATTAAAATTGGCTTCAAGCAGAAGTGA

>Xla-aedc1.S
ATGCCTGGAACGGGAGACAAGAAGAAATGTCCAGACCCATGCCCAAAACCAAAGCCCCAGAAATGCAAAGACCCTTGCAAGAGTACTCCACAGTGCCCTAAACCAAC
ACCAAAATGCAAAGATCCCTGCAAGCCACAAGATTGTACCCAAGACAAAAAATGTGTCAAGAAGTGTTAA

>Xla-aedc2.S
ATGCCTGGAACGGGAGACAAGAAGAAATGTCCAGACCCATGCCCAAAACCAAAGCCCCAGAAATGCAAAGACCCTTGCAAGAGTACTCCGCAGTGCCCTAAACCAAC
ACCAAAATGCAAAGATCCCTGCAAGCCGCAAGATTGTACCCAAGACAAAAAATGTGTCAAGAAGTGTTAA

>Xla-aedc3.S
ATGTCTGGAGTTAAGGGTCATCAAACAAAATGCCCAGATCCATGCCCTCCACCACAGAAATGCAAAGATCCTTGCAAGGATCCTTGCCCTAAGCCAGTCCCACAATG
CCACGACCCATGCCAGAAGACTCATCAACATCAGGGGCATGGTCAAGACCATAGCCAAGGCCATAGCCAGGGCAATAACCAATCCCATCACTGA

>Xla-aedc4.S
ATGTCTGGAGTTAAAGGTCAACAGAAGAAATGCCAAGAGCAATGCCCACCACAGCCCTGCAATCCATGCCCTCCACAGCCCTGTAACCCATGCCCTCCACCACAGAA
GTGCCCGGATACTTGCCCAAAGCCAGTCCCACAATGTCAAGATCCATGTAAGCAACAACAGACCCATGGAGGTCAACACCAACACTGA

>Xla-aedc5.S
ATGTCTGGAGTTAAGGGTCATCAAACAAAATGCCCAGATCCATGCCCTCCACCACAGAAATGCAAAGATCCTTGCAAGGATCCTTGCCCTAAGCCAGTCCCACAATG
CCAAGACCCATGCCAGAAGACTCATCAACACCAGGGGCATGGTCAAGACCATACCCAAGGCCATAACCAGGGCAATAACCAACCCCATCACTGA

>Xla-aedc6.S
ATGTCTGGAGTTAAAGGTCAACAGAAGAAATGCCAAGAGCAATGCCCACCACAGCCCTGCAATCCATGCCCTCCACAGCCCTGTAACCCATGCCCTCCACCACAGAA
GTGCCAGGATCCTTGCCCTAAGCCAGTCCCACAATGCCAAGATCCATGTAAGCCACATCAGACCCATGGAGGTCAACACCAACACTGA

>Xla-aedc7.S
ATGTCTGGAGTTAAGGGTCATCAAACAAAATGCCCAGATCCATGCCCTCCACCACAGAAATGCAAAGATCATTGCAAGGATCCTTGCCCTAAGCCAGTCCCACAATG
CCACGACCCATGCCAGAAGACTCATCAACATCAGGGGCATGGTCAAGGCCATAGCCAAGGCCATAGCCAGGGCAATAACCAACCTCATCACTGA

>Xla-aedc8.S
ATGTCTGGAGTTAAAGGTCAACAGAAGAAATGCCAAGAGCAATGCCCACCACAGCCCTGCAATCCATGCCCTCCACAGCCCTGTAACCCATGCCCTCCACCACAGAA
GTGCCAGGATCCTTGCCCTAAGCCAGTCCCACAATGCCAAGATCCATGTAAGCCACATCAGACCCATGGAGGTCAACACCAACACTGA

>Xla-aedc9.S
ATGTCTGGAGTTAAAGGTCAACAGAAGAAATGCCAAGAGCCAGCCCCACCACAGCCCTGCAATCCATGCCCTCCACAGCCCTGTAACCCATGCCCTCCCCCACAGAA
GTGCCCGGATCCTTGCCCTAAGCCAGTCCCACAATGCCAAGATCCATGTAAGCCACATCAGACCCATGGAGGCCAACACCAACACTGA

>Xla-aedc10.S
ATGTCTGGAGTTAAAGGTCAACAGAAGAAATGCCAAGACCAAGCCCCACCACAGCCCTGCAATCCATGCCCTCCACAGCCCTGTAACCCATGCCCTCCACCACAGAA
GTGCCCGGATCCTTGCCCTAAGCCAGTCCCACAATGCCAAGATCCATGTAAGCAAAAACAGACCCATGGAGGTCAACACCAACACTGA

>Xla-aedc11.S
ATGTCCGCTAAAGGAGGTAGCCAATGCAAGCAAGACCAATGTAAAGATCCTGACCCCTGCATACCAGTACAGAAATGTCAGCAAGCCAAGCCCATATGCAAAGATCC
TTGCCAAACAACTAACCCCTGTCAGCAGCAAGGTCAAGGGCATGAGCACCAGGGCCACCATGGAGGAGGTGGAGGAGGCGGAGGAGGTGGAGCAGCCAAGAAGTAA

>Xla-aedc12.S
ATGTCTGGAACTAAGGGTGGAAGTGGCATCATTGGTGGCGGTGGTTGTGGTTCCAGCAGTGGTGGAGGTAGTGGTGGCAGTGGAACAATATCTGCCCAATGTGGAAC
TACTGGCACAAATATACCAATACAACAATGCCAGGGCACAAGCACTGTCCAACAAACAAATCCTTGCCAAACAACTAACCCCTGTCAGCAGCAAAGTCAAGGGCATG
AGCACCATGGCCACCATGGACACCATGGAGGACAAGGCAGCCACGGAGGCGGAGGAGCCAAGAAGTAA

>Xla-aedc14.S

ATGTCTGGAACTAAGGGTGGAAGTGGCATCATTGGTGGTGGCGGTTGTGGTTCCAGCAGTGGTGGAGGTAGTGGTGGATATGGCTGTGTTGGCAGTGGAATAATATC
TGCCCAATGTGGAGATACTGGCACAAATATACCAATACAACAATGCCAAGGCACAAGTACAGTTCAACAAACAAGCACAGATCCTTGCCAAACAACTAACCCCTGTC
AGCAGCAAGGTCAAGGGCATGAGCACCATGGCCACCATGGACACCATGGCCACCATGGAGGACAAGGCAGCCATGGAGGTGGAGGAGCCAAGAAGTAA

>Xla-aedc15.S
ATGTCTGGAGTCAAGGGTGGTGGAGGTACATATTATGGTGGAGGAGGCAGTGGAAGTGGAGGTTCATCAGACCAATGCAAAGATACAAATCCATGTGTACCAGTTCA
GCAATACCAGGGTAACAACGCTGGTCAGGGATCCACAGGCACTGTTAACCCTTGCCAGCAAGGTCAGGTTGGCCAGATAAGTGGAGGTTACACAAGTGGTGCTGGAG
GAACCTATGGATCAAGTGGTATCACTCAGATAAGTGGAAGTGGAGGAGGTAATATCAGCCAGCTTAGTGGAGGTTGCATCGGTGGAAGTGGAGGAAGTGACACCAGC
CAGATAAGTGGAAGTGGAGGATATGGCACCAGCCAGATTAGTGGAGGTTGCCTCAGTGGAAGTGGAGGAGGTAACACCAGCCAGATTAGTGGAAGTGGAGGATATGG
CACCAGCCAGATTAGTGGTGGTTGCATTGGTGGAAGTGGAGGAGGTAACACCAGCCAGATAAGTGGAAGTTGCATTGGTGGAAGTGGAGGATATGGCACCAGACAGA
TTAGTGGAAGCGGAAGCGGTGGAAGTGGTTGCACCAGCCAGATTACCGGAATTTACAGCAGTGGAGTAAGCTATGGATCGGGCTGTGGAGGAAGCTCTGGTGGGCAG
AATGGTGGAGGTGGAGCCAAAAAGTGA

>Xla-aedc16.S
ATGTCTGGAGTCAAAGGTGGTCGTAGCCAGTGCCAGCAGAAGACTTACTGCCCAGATCCATGCCAGCCACAAATTTTCTGCAAAGATCCATGCCAGAAACAGGTTGT
GTGTAAGGATCCATGCCAACCACAAGTTGTCTGTAAAGATCCATGCCAGCCACAAGTTGTCTGCAAAGATCCATGCCAGCCTCAATTTGTCTGTAAAGATCCTTGCC
AACTAACCATGTGTCATGACACCTGTCAACCTGTCCAGCATTACCATTGTAAGACTGTACACTGCCCCCAAAAAGTTCACTGTGACCCTTGCCATCCTTGCCAGTGC
TATGGAAAGCACTGA

>Xla-aedc17.S
ATGTCTGGAGTCAATGGCGGCCGCAGCCAATGCCAGCAGAAGACTTACTGTCCAGACCCATGCCAGCTACAAGTTGTCTGCAAAGATCCATGCCAGCCTCAAGTTGT
GTACAAAGATCCATGCCAGCCTCAAGTTGTGTACAAAGATCCATGCCAGCCTCAAGTTGTCTGCAAAGATCCGTGCCAACTAACCATATGCCATGATCCCTGCCAAC
CTGCCCAGCATTACCATTGCCAAACTGTGCATTGTACCCAAAAAGTTCACTATGACCCTTGCCATCCTTGCCAATGCTATGGAAAGCACTAA

>Xla-aedc18.S
ATGTCTTTTTTAAAGAAAGGACTTGGAGATGTTAAAACCGATGAGATTGTAAAACAAGGGAAGGAAGCTGTGGAGGATACCATAAATCAGTGCATTCAAGAGGGGCA
ACAATATGTTCAAGAAGTGGTTGACAAAGGCACAGAAGCTGCTAAAGATGCTGCAAACAAGGCAGTAACTGATTTTAAAACTGGCTTCAAGCAGAAGTGA

C

>Bbu-aedc1
ATGTCTGGAGTAAAGGGACACCAGAAGAAAACCCCAGAGCCATGTGCACCACCACCCCAACAGCACCAGGATCCTTGTAAACCAGAGCCACAATGTGAGGAACCCAT
TCAACAAGTTTGCGAGCCTGAACCAGTGTGTGAACAACAATGTCCTCCACCTCAGGAGATATGCACACAGTCTAGTCAAAAGAAGGACAACATTGGTGTTAAGAAGT
AA

>Bbu-aedc2
ATGTCTGGAGTAAAGGGACAACAGAAGAAATGCCCAGAACCTTGCCCACCACCCAAAGAATGCCAAGATCCTTGCAAGCCAAAGCCAGTGACTCCAGAGCCAAGATG
TCCTTCTCCTCAAACTCACAAGCCAGAGCCGTCCCATCCAGAACCAAAATGTCCTCATCCACAAGAGCAAACAAAGCCAGCTCCTCGATGCCATGAACCACAAGATA
AGAAATGTGTGAAGAAATAA

>Bbu-aedc3
ATGTCTGGAGTAAAAGGACAACAGAAGAAATGCCCAGAGCCTTGCCCACCACCCCAGCAGGACTGCCAGGATCAGTGCGAGCCAGTGCAGGTGTGCCCTGAACCGAA
ATGTCCTCCTCAAGTGGTACAATGTCCACCACCACAATGTCAGAACCAACAGGACAAGCAATACGTGAAGAAGTGA

>Bbu-aedc4
ATGTCTGGAGTAAAAGGACAACAGAAGAAATGCCCAGAGCCTTGCCCACCACCCCAGCAGGACTGCCAGGATCAGTGCGAGCCAGTGCAGGTGTGCCCTGAACCGAA
ATGTCCTCCTCAAGTGGTACAATGTCCACCACCACAATGTCAGAACCAACAGGACAAGCAATACGTGAAGAAGTGA

>Bbu-aedc5
ATGTCTGGAGTGAAGGGACAGAAGAAATGCCCAGAGCCTTGCCCACCACCCCAAAAGTGCCAGGATCCTTGCAAGCAAGTGTGCCCTGAGCCAAAATGTCCTCCTCC
ACAAGTGTGTGTACCAGTGCCTGTGTGCCCTGAACCGAAATGTCCTCCTCCTCAAGTGCAATGTAAACCAGTCCCACAATGTCAGCAACAACACGGAAAGTAA

>Bbu-aedc6
ATGTCAGCAAAGGGACAAGGCAAAAACCCATGTTGCCCACCACCTGTTTGCCAGGACCAAACACCAAAATGCATCATAGTTGACCCATGTGCTCCATGTCCAAAAGT
TCAATGTCCACCACCGCCTTGTTGCCCAGATCCATGCCAGCAGGGTGGTTATGGACAAGGCTACGGTGGTCAAGGAGGATATGGGTACGGAGGTAACATGAAGTGA

>Bbu-aedc7
ATGTCAGCAAAGGGACAAGGCAAAAACCCATGTTGCCCACCACCTGTTTGCCAGGACCAAACACCCAAATGCATCATAGTTGACCCATGTGCTCCATGTCCAAAAGT
TCAATGTCCACCACCGCCTTGTTGCCCAAATCCATGCCAGCAGGGTGGTTATGGACAAGGCTACGGTGGTCAAGGAGGATATGGGTACGGAGGTAACATGAAGTGA

>Bbu-aedc8
ATGTCAGCAAAGGGACAAGGCAAAAACCCATGTTGCCCACCACCTGTTTGCCAGGACCAAACACCAAAATGCATCATAGTTGACCCATGTGCTCCATGTCCAAAAGT
TCAATGTCCACCACCGCCTTGTTGCCCAGATCCATGCCAGCAGGGTGGTTATGGACAAGGCTACGGTGGTCAAGGAGGATATGGGTATGGAGGTAACATGAAGTGA

>Bbu-aedc9
ATGTCAGCAAAGGGACAAGGCAAAAACCCATGTTGCCCACCACCTGTTTGCCAGGACCAAACACCCAAATGCATCATAGTTGACCCATGTGCTCCATGTCCAAAAGT
TCAATGTCCACCACCGCCTTGTTGCCCAAATCCATGCCAGCAGGGTGGTTATGGACAAGGCTACGGTGGTCAAGGAGGATATGGGTACGGAGGTAACATGAAGTGA

>Bbu-aedc10
ATGGCAACCAAGGGAGGAAAATGCAACCCATGTGCACAAGTACCACAATATATACAAGACCCATGTAAAGAACCTTGCCCTCCAGTTAATCCTTGCCAGGTGCAAGG
CCAAGCAGTAGGAGGAATGGGAGGAATCAGTGGACAAGTGTCACAAGGAGGGTATGGTGGTCCCGGAGGATTCGGTGGACAAATTTCACAAGGAGGGTATGATAGTG
CCGGAGGATTTGGTGGACAAGTTTCACAAGGAGGGTATGATAGTGCCGGAGGATTCGGTGGACAAGTTTCACAAGGAGGGTATGATAGTGCCGGAGGATTCGGTGGA
CAAGTTTCACAAGGAGGATACGGTGGACCCGGAGGATTCAGTGGACAAGTTTCACAAGGAGGGTATGGAGGAGCAGGAGGAATTGGTGGACAAGTTTCACAAGGAGG
GTATGGAGGAGCAGGAGGATTCAGTGGACAAGTTTCACAAGGAGGGTATGGAGGAGCAGGAGGATTGGGTGGGCAAGTTTCACAAGGAGGATATGGAGAAGCCAGAG
GATTTGGTGGAGTAAAGAAATAA

>Bbu-aedc11
ATGTCTGATATTAAATGCAAATCTGGTGGTGGATACCAAAAAGGCAGCTCTGGTGATGACTGTGGTGGTGGATATCAGAAAGGAGGATCTGGCAGTGGTAGCTCTGG
TGGCGGATGTCAGTCAAGTGGCTCTGGTGATGGATGTAAACTAGGTAGCTCAGGAGGGGGCTTTCAACAAGGCAGCTCTGGTGGTGACTGTAGTGGCGGATTTCAAC
AAGGAGGTTCTGGAGGTGGATATCAAAAAGGAAGCTCTGGGAGTGACTGTGGTGGCGCATATCAGCAAGGAGGTTCAGGAGGTGGATATCAACAAGGCAGCTCTGGA
GGTGACTGTGGTGGCGGATATCAGCAAGGAGGTTCTGGAGGAGGATATCAACAAGGTAGCTCTGGAGGTGACTGTGGTGGTGGATATGGCAAGCAGAACATGACCTT
GTGTGGAGATGACCAGCAGAAAGGTGGATCCTGTACATCTGGTGGCTCCGGACAACAATGTCAAGATCAAGGTTCATATGGCAGCGGTAGTAAATCAGGCGGTGGTG
CCGGTTGGGGCCAGGATTCTTGCGATGGTAGCAAATCAGATGCCAGCCAGGGAAAAGGAACAGGAGCATGCAAGAAGTGA

>Bbu-aedc12
ATGTCTGGTGTTAAAGGCAAATCTGGTGGTGGAAACCAAAAAGGCAGCTCTGGTGATGACTGTGGTGGTGGATATCAGAAAGGAGGATCTGGCAGTGGTAGCTCTGG
TGGCGGATGTCAGTCAAGTGGCTCTGGTGATGGATGTAAACTAGGTAGCTCAGGAGGGGGCTTTCAACAAGGCAGCTCTGGTGGTGACTGTAGTGGCGGATTTCAAC
AAGGAGGTTCTGGAGGTGGATATCAAAAAGGAAGCTCTGGGAGTGACTGTGGTGGCGGATATCAGCAAGGAGGTTCAGGAGGTGGATATCAACAAGGCAGCTCTGGA

GGTGACTGTGGTGGCGGATATCAGCAAGGAGGTTCTGGAGGAGGATATCAACAAGGTAGCTCTGGAGGTGACTGTGGTGGCGGATATCAGAAAGATGCCTCTGGAGG
AGATTGTGGTGGTGGATATGGCAAGCAGAACATGACCTTGTGTGGAGATGACCAGCAGAAAGGTGGATCCTGTACATCTGGTGGCTCCGGACAACAATGTCAAGATC
AAGGTTCATATGGCAGCGGTAGTAAATCAGGCGGTGGTGCCGGTTGGGGCCAGGATTCTTGCGATGGTAGCAAATCAGATGCCAGCCAGGGAAAAGGAACAGGAGCA
TGCAAGAAGTGA

>Bbu-aedc13
ATGTCTGGAATCAAAGGAGGACGATGTGGCCAAAAGATCCAGTGCCAGAATCCATGCAAAGACCCCTGCCAAAAAGTCCCTATCTGTGTAAATCCATGCCAAGACCC
ATGTAAATTTGAAATTCAGAATCATGGCTCTCACCATAATCATCATGGTCAACACCATTTCCACAAGCAAGATCCCTGCAATCCCTGCTGTCCAGACCCTTGTCAGG
ATCAAGGAAGCTACTCCTTTAATAAATGA

>Bbu-aedc14
ATGTCTGGAGTTAAAGGAGGACATCAGAAAGCCCAATGCAAGGACCCGTGCCAGAAGCAGACAATCTGCCAGGATCCATGCCAGAAGCAAACCATCTGCCAAGATCC
ATGCCAGAAGCAGACTTTCTACCAGGATCCATGCCAGAAGCAGACCATCTGCCAGGATCCATGCCAGAAGCAGACTTTCTACCAGGATCCATGCCAGAAGCAGACCA
TCTGCCAAGACTCATGCCAAGATCCCTGCAAGCCTATACAGCAGTGCCAAGGATATCAAAAAGATCCTTGCAACCCCTGTGTTCCTGATCCTTGCCAGTCTCAAGGA
AAATATTACTGA

>Bbu-aedc15
ATGTCTGGAATCAAAGGAGGACGATGTGGCCAAAAGATCCAGTGCCAGAATCCATGCAAAGACCCCTGCCAAAAAGTCCCTATCTGTGTAAATCCATGCCAAGACAC
ATGTAAATTTGAAATTCAGAATCATGGCTCTCACCATAATCATCATGGTCAACACCATTTCCACAAGCAAGATCCCTGCAATCCCTGCTGTCCAAACCCTTGTCAGG
ATCAAGGAAGCTACTCCTTTAATAAATGA

>Bbu-aedc16
ATGTCTGGAGTTAAAGGAGGACATCAGAAAGCCCAATGCAAGGACCCGTGCCAGAAGCAGACAATCTGCCAGGATCCATGCCAGAAGCAAACCATCTGCCAGGATCC
ATGCCAGAAGCAGACTTTCTACCAGGATCCATGCCAGAAGCAGACCATCTGCCAGGATCCATGCCAGAAGCAGACCATCTGCCAGGATCCATGCCAGAAGCAGACTT
TTTACCAGGATCCATGCCAGAAGCAGACCATCTGCCAAGACTCATGCCAAGATCCCTGCAAGCCTATACAGCAGTGCCAAGGATATCAAAAAGATCCTTGCAACCCC
TGTGTTCCTGATCCTTGCCAGTCTCAAGGAAAATATTACTGA

>Bbu-aedc18
ATGTCTGGAGTTAAAGGAGGACATCAGAAAGCCCAATGCAAGGACCCGTGCCAGAAGCAGACAATCTGCCAGGATCCATGCCAGAAGCAAACCATCTGCCAGGATCC
TTGCCAGAAGCAGACTTTCTACCAGGATCCATGCCAGAAGCAGACCATCTGCCAAGACTCATGCCAAGATCCCTGCAAGCCTATACAGCAGTGCCAAGGATATCAAA
AAGATCCTTGCAACCCCTGTGTTCCTGATCCTTGCCAGTCTCAAGGAAAATATTAA

>Bbu-aedc19
ATGTCTGGAGTTAAAGGAGGACATCAGAAAGCCCAATGTAAGGACCCGTGCCAGAAGCAGACAATCTGCCAGGATCCATGCCAGAAGCAGACCATCTGCCAGGATCC
ATGCCAGAAGCAGACTTTCTACCAGGATCCATGCCAGAAGCAGACCATCTGCCAAGACTCATGCCAAGATCCCTGCAAGACTATACAGCAGTGCCAAGGATATCAAA
AAGATCCTTGCAACCCCTGTGTTCCTGATCCTTGCCAGTCTCAAGGAAAATATTAA

>Bbu-aedc20
ATGTCTGGAGTTAAAGGAGGACATCAGAAAACCCAATGCAAGGACCCTTGCCAGAAGCAGACAATCTGCCAGGATCCATGCCAGAAGCAGACCATCAGCCAGGATCC
ATGCCAGAAGCAGACTATCTACCAGGATCCATGCCAGAAGCAGACCATATGCCAAGACTCATGCCAAGATCCCTGCAAGCCTATACAGCAGTGCCAAGGATATCAAA
AAGATCCTTGCAACCCCTGTGTTCCTGATCCTTGCCAGTCTCAAGGAAAATATTACTGA

>Bbu-aedc21
ATGTCTGGAATCAAAGGAAAACCAGGATTTAAACAACCAAATCCTGTCCAACCAATCCTGGAGCCACAGCCTATCTGTGAAGACATAGCGCAAGTTACTCCACAACC
TCCTGCTCCTTGTCCTGTGCCTGTGACACCACAGCCTTGTGCACCTGTGCAAGTTACTCCACAACCTCCTGCTCCTGTGCCTGTGGCACCACAGCCTTGTGTCCCCG
TGCAAGTCACTCCACAACCTCCTGCTCCTGTGCCTGTGGCACCACAGCCTTGTGCACCTGAACCCACTGTTAACCAGGAGAAGAAAGGATGCAAAAAGTAA

>Bbu-aedc22
ATGTCTGGAGTTAAAGGAGGACATCAGAAAGCCCAATGCAAGGACCCGTGCCAGAAGCAGACCATCTGCCAGGATCCATGCCAGAAGCAGACCATCTGCCAGGATCC
ATGCCAAAAGCAGACCATCTGCCAGGATCCATGCCAGAAGCAGACCATCTGCCAGGATCCATGCCAGAAGCAGACCATCTGCCAGGATCCATGCCAGAAGCAGACCA
TCTGCCAGGATCCATGCCAGAAGCAGACCATCTGCCAAGACCCATGCCAGGATCCCTGCAAGCCGGTACAGCAGTGCAAAGATCCTTGCAACCCCTGTGTTCCTGAT
CCTTGCCAGTCTCAAGGAAAATATTAA

>Bbu-aedc23
ATGTCTGGAATCAAAGGAAAACCAGGATTTAAACAGCCAAATCCTGTCCAACCAATCCTGGAGCCACAACCTATCTGTGAAGACATAGCGCAAGTCACTCCGAAGCA
TCCTGCTCCTTGTCCTGTGCCTATGACAACACAACCTTGTGCACCTGTGCAAGTTACTCCACAACCTCCTGCTCCTGTGCCTGTGACACCACAGCCTTGTGCACCTG
TGCAAGTTACTCCACAACCTCCTGCTCCTGTGCCTGTGACACCACAGCCTTGTGCACCTGTGCAAGTTACTCCACAACCTCCTGCTCCTGTGCCTGTGACACCACAG
CCTTGTGCACCTGTGCAAGTTACTCCACAACCTCCTGCTCCTGTGCCTGTTGCACCACAGCCTTGTGCACCTGTGCAAGTTACTCCACAACCTCCTGCTCCTGTGCC
TGTTGCACCACAGCCTTGCGTCCCCGTGCAAGTCACTCCACAACCTCCTGCTCCAGTGCCTGTGGCACCACAGCCTTGTGCGCCTGAACCCACTGTTAACCAGGGGA
AGAAAGGATGCAAAAAGTAA

>Bbu-aedc24
ATGTCTTTCTTAAATAAGGGAGTTAATTTAAACACAAGCAACCTCGTGCAGCAAGGAAAAGAGGCGGCCCAGGACTCAGCCAACCAGTGTGTGCAGGAAGGTCAGAA
AATTGCTCAAGAAGTTGTAGACAAAGGATGCCAAGTTGCTAAGGATGCTGGAGGGAAGGCAGTCGATCAACTGAAAGGTGGAATCAAAAATAAGTGA

>Bbu-aedc25
ATGTCTAGCTTCTTAAACCAAGCCCAGCAGAAAGCTGACGAAACCGTTAAGCAAGGAGAACAAACAGTCCAGGATTGTGTTGATAAAGGCAAAGAAGTTGGTCAACA
AGTTATTGACCAAGGATGTCAAACTGCCAAAAACACATGTGATCAAGTAACAGGACAAGTTGGTGATAAGCTCACTGGTGGATGGAAGAAGCAATCATAA

D

>Bbo-aedc1
ATGTCTGGAGTCAAGGGACATCAGAAGCAATGCAAGGATCCATGTGACCCCTGCCCAGATCCATGCCCACCCCCACAGAAATGCCCAGACCCTTGCAAACCAGACCC
ATGCTATAAACCAACTCCCCAGTGCCAAGATCCATGCAAGCCATCACCTCAGCAGTGTCAGCAGGATAAGAATAAATATTGA

>Bbo-aedc2
ATGTCTGGAGTTAAGGGACATCAGAAGCAATGCCCAGATCCTTGCCATGACCCATGCCCACCCCCACAGAAATGCCAAGATCCTTGCAAACAGGATCCTTGCCCAAA
ACCTGTCCCACAATGTCAAGATCCCTGCAAGCCAGAACCGCAGCAGTGTCAACAGGACACCAAATGTGTGAAGAAATGCTAA

>Bbo-aedc3
ATGTCTGGAGTAAAAGGACAGCAACAGAAATGCCACGAGCCTTGCCCACCGAAGCCATGCCCTCCTCCACAGCAATGCCTACCCCCACAGCAATGCCTACCCCCACA
GCCATGCCCTCCTCCACAGCAATGCCCACCTCCACAGCAATGCCTACCACCACAGCAATGCCTACCACCACAGCAATGCCTACCACCACAGCAATGCCCACCACCAC
AGCAATGCCTACCACCACAGCAATGCCCACCACCACAGCAATGCCTACCACCACAGCAATGCCTACCCCCACAGCAATGCTCTCCTCCACAGCAATGCCTACCCCCA
CAGCCATGCCCACCACCACAGCAATGCCAGGATCCTTGCAAACCAGTGTGCCCTGAACCTGTGCAGAAATGTCCTCCTGTTCAAGAACCATGCAAACCACAATGTCC
TCAGGACCAGCAGAAATGTGTTAAGAAATAA

>Bbo-aedc4

ATGTCTGGAGTAAAAGGACAGCAACAGAAATGTCACGAGCCTTGCCCACCGAAGCCATGCCCTCCTCCACAGCAATGCCCACCTCCACAGCAATGCCTACCCCCACA
GCAATGCCCACCTCCACAGCAATGCCTACCCCCACAGCAATGCTCTCCTCCACAGCAATGCCTACCCCCGGCAGAAATGCCCACCCCACAGCCATGCCCTCCACCAC
AGCAATGCCAGGATCCTTGCAAACCAGTGTGCCCTGAACCTGTGCAGAAATGTCCTCCTGTTCAAGAACCATGCAAACCACAATGTCCTCAGGACCAGCAGAAATGT
GTTAAGAAATAA

>Bbo-aedc5
ATGTCTGGAGTAAAGGGACAGCAAAAGAAATGCCCTGAGCCTTGCCCACCAAGCCATGCCCTCCACCACCACAGCAGTGCCAGGATCCTTGCAAACAAGAGCCAAAG
TGTCCTGAACCTGTTCAAAAATGCCCTCCTCCTCAAGAACAATGCAAACCCCAGTGCCCACAAGACCAGCAGAAATGAGCAGAAAATGGACCTTAACACCTGGCAAA
TACTTCTGTCAATTTGA

>Bbo-aedc6
ATGTCTGGAGTAAAGGGACAGCAAAAGAAATGCCCTGAGCCTTGCCCACCACAGCCATGCCCTCCACCACCACAGCAGTGCCAGGATCCTTGCAAACAAGAGCCAAA
GTGTCCTGAACCTGTTCAGAAATGCCCTCCTCCTCAAGAACAATGCAAACCACAGTGCCCACAAGACCAGCAGAAATGA

>Bbo-aedc7
ATGTCTGGAACTAAGGGTGGACACCAGAAGGTAGACAAGTGCAAAGACCCCTGTGTCCCAGACGTGCAATGCCAGGATCCCTGTAAGGTGGTGCCAACCCCACAATG
CCAGGATCCCTGTAAGGTGGTACCAACCCCACAATGCCAAGACCCCTGTGCACCTGATCCTTGCCAAAAACAGGGCCACGGTGGTCATGGAGGAGGAGGAGGAGGAG
GAGGACACGGGGGACACGGAGGTGGAACTAAGAAATGA

>Bbo-aedc8
ATGTCTGGAACTAAAGGTGGCGGATATTCCCAGCAGCAGGTCAACAAATGCACAGACCCTTGTGCACCAGTGCAAAAGTGTGTTGACCCATGCCAAGTCCAAGCCAC
TGTACAATGCAAAGTGCCTGACCCTTGTGCACCTGTTGACCCCTGCCAGCAACAGGGAGGAGGATACCAGTCTCAAGGTCATGGAAGTCATGGTAGCGGCGGATACC
AGTCACAAGGTCATGGAAGTCATGGTAGCGGCGGATACCAGTCACAAAGCCAAGGTGCGGGATACGGAGGCAGTTGCAAGAAGTAA

>Bbo-aedc9
ATGTCTGGAACTAAAGGGGGCGGATATTCCCAGCAGCAGGTCAACAAATGCACAGACCCTTGTGCACCAGTGCAAACGTGTGTTGACCCATGTCAAGTTCAAGCCAC
TGTACAATGCAAAGTGCCTGATCCTTGTGCACCTGTTGACCCCTGCCAGCAACAGGGAGGAGGATACCAGTCTCAAGGTCATGGAAGTCATGGTAGCGGCGGATACC
AGTCACAAGGTCATGGAAGTCATGGTAGCGGCGGATACCAGTCACAAGGTCATGGAAGTCATGGTAGCGGCGGATACCAGTCACAAGGTCATGGAAGTCATGGTAGC
GGCGGATACCAGTCACAAAGCCAAGGTGGGGGATACGGAGGCAGTTGCAAGAAGTAA

>Bbo-aedc10
ATGTCTGCAAGCAAAGGAGGTTCACACCAGCAGTCCAAAGGCCATGACCATGCTGAGGATCATGTGAAAGAGGCTCATCAAGATAAAGGTCATGGGGGTCAAGAAAA
AGGCCACGAGGCTCAAGAGAAAGGTCATGGGGGTCAAGACAAAGGCCATGAAGCTCAAGAAAAAGGTCATGGGGGTCAAGATAAATGTCATCAAGGTCACAGTGGAG
GTGATGGAAGTCATGATAAGAAAGGCCACGATGACTCCAGCAAAGGTCATGGGGGCCAAGATAAAGGTCATGGTGGAGATAAAGGAGGTCATGATGAGAAAGGTCAT
GGGGGTCATGGAGGAGACAATAAAGTTCACAAAGGTCCAGCTAAAAAGTGA

>Bbo-aedc11
ATGCCTGCAGCCAAAGGAGGAGGACAACAGAAACACGATCAATGCAAGGATCCTTGCCAACAACAAGGTCATGGCAGTCAAGGACAAGGGGGTCATGGACATGGAGG
CCAGGATCATGGAGGTCAAGGGCATTGTGATCAAGGTCACGGAGGTCAAGACCATGGAGGCCAAGGGCATGGAGGTCAAGGTCATGGAGGTCAAGGGCATTGTGATC
AGTCTCAGGGCGGACAAGGTCATGGAGGTCATGGAGGTCAAGGGCATTGTGATCAAGGTCACGGAGGTCAAAGTCATGGAGAACAAGGTCATGGGGGCAAAGGGCAT
GGAGATCAAAGTCATGGAGGCCAAGGGCATGGTGGACAAGGACATGGAAGTCAAGGTCATGGAGGTCAAAGTCATGGAGGCCAAGGGCATGGAGGTCAAGGAGGTCA
AAGTCATGGAGGCCAAAGTCATGGAGGCCAAGGGCATGGAGGTCAAGGTCTTGGAGGCCAAGGGCATGGTGGACAAGGAGGTCACTGTTAA

>Bbo-aedc12
ATGTCTGGAGTTAAAGGTGGTTGTGGTGGTGGAGGTGGAGGAGGAGGTGGAGGAGGAGGTGGTGGAGGATACAAAGCTAACCCTTGTGTTACAGTTGATCCATGCCA
AAACGTCAACATCTCAACTCAAAAAGGTGTAGTTGATCCTTGCCAGCAGAGTGGTGGAGGACAAGGTAGTTATGGCAGTGGTGGAGGCTTTGGAGGTCAAGTGAGCT
ATGGAGGTGGTAGTCAAGGTGGTCAAGTCAGCCAATGTACAGGTGTAGTTGATCCTTGCCAGCAGAGTGGTGGAGGACAAGGTAGTTATGGCAGTGGTGGAGGCTTT
GGAGGTCAAGTGAGCTATGGAGGTGGTAGTCAAAGTGGTCAAGTTAGCCAATGTACAGGTGTAGTTGATCCTTGCCAGCAGAGTGGTGGAGGACAAGGTAGTTATGG
CAGTGGTGGAGGCTTTGGAGGTCAAGTGAGCTATGGAGGTGGTAGTCAAGGTGGTCAAGATGCATTCAGGTACTTAGGTATCTGGATCTCTTCTGATCCTAGTGCCT
GGTACGACTTGAATGTTTTACCTGTCCTTAAGAAAGTTATGGAAGCCTTTAAAAACTGGCAGAAACTCCCTTTGTCTTTATCAGGACCAAAGGCACAAAACTCCACA
GGCACATCCCCTTGCAGAACGGTGGTTACCATAATTATGTCTGGAGTTAAAGGTGGTTGTGGTGGTGGAGGTGGAGGAGGAGGAGGTGGTGGAGGATACAAAGC
TAACCTTGATCCATGCCAAAATGTCAACATCTCAACTCAAAAATGTGCAGGTGTAGTTGATCCTTGCCAGCAGAGTGGTGGAGGACAAGGTGGTTATGGCAGTGGTG
GAGGCTTTGGAGGTCAAGTGAGCTATGGAGGTGGTAGTCAAGGTGGTCAAGTCAGCCAATGTACAGGTGTTGTTGATCCTTGCCAGCAGAGTGGTGGAGGACAAGGT
GGTTATGGCAGTGGTGGAGGCTTTGGAGGTCAAGTGAGCTATGGAGGTGGTAGTCAAGGTGGTCAAGTCAGCCAAAGTGGTCAAGCCAGCCATGGTGGTGGTTGTGC
CAAGAAATGA

>Bbo-aedc13
GTCTCTGGGTTCTAGAAGGTTTGACCAAACCGAATACACACAATGTCTGGAGTTAAGGGAGGTCATGGCCAACAGAGCCAACAATGCCAGAAAGGGCAGTTCGGTCA
GGAGAGTCAGCAGTGTCACCAAGGGCTGCAGAGTATAAGTCAGGGCAAAGATTTGTGCCAACAGCAGCAAAAAGGTGTTTGCCAGGATGCCTTTGGGGCTGGACAGC
AGTGCCAGGAGCAAAGTAGCTTTGGATCTAAATCAGCATTTGGTGCTGGGCAGCAGTGTCAGGAGCAAAGCAGCTGTGGATCAAAATCAGCATTTGGTGCTGGGCAG
CAGTGTCAGGAGCAAAGCAGCTGTGGATCAAAATCAGCATTCGGTGCTGGGCAGCAGTGTCAGGAGCAAAGCAGCTGTGGATCAAAATCAGCATTCGGTGCTGGGCA
GCAGTGTCAGGAGCAAAGCAGCTGTGGATCAAAATCAGCATTCGGTGATGGGCAGCAATGTCAGGAGAAAAGCAGCTGTGGATCAAAATCAGATGCTTGCCAGAGCC
AGGCCAAAGCTCAGGGAGTCAAGAAGTAA

>Bbo-aedc14
ATGTCTGGAGTCAAAGGAGGACATTGTCACCAGAAGACCCAGTGCCAGGATCCCTGCAAAGATCCCTGCCAGAAGCAGAGCGTGTGCCAGGATCCTTGCCAAAAGCA
GGGCGCCTGCCATGACCCTTGCCATCCAGTGCAGCATTGTCATGATCAGAGCCCTTGTGGGAAGAAAAAAGATCCATGCAATCCCTGCTGCCCAGATCCCTGCCAGA
GCCAAGGGAAAAATCACTGA

>Bbo-aedc15
ATGTCTGGAGTCAAAGGAGGACATTGTCACCAGAAGACCCAGTGCCAGGATCCCTGCAAAGATCCCTGCCAGAAGCAGAGCGTGTGCCAGGATCCTTGCCAGAAGCA
GAGCGCCTGCCATGACCCTTGCCATCCAGTTCAGCATTGTCATGATCAGAGCCCTTGTGGGAAGAAAAAAGATCCATGCAATCCCTGCTGCCCAGATCCCTGCCAGA
GCCAAGGGAAACATCACTGA

>Bbo-aedc16
ATGTCTGGAGTCAAAGGAGGACATTGTCACCAGAAGACCCAGTGCCAGGATCCCTGCAAAGATCCCTGCCAGAAGCAGAGCGTGTGCCAGGATCCTTGCCAAAAGCA
GGGCGCCTGCCATGACCCTTGCCATCCAGTGCAGCATTGTCATGATCAGAGCCCTTGTGGGAAGAAAAAAGATCCATGCAATCCCTGCTGCCCAGATCCCTGCCAGA
GCCAAGGGAAACATCACTGA

>Bbo-aedc17
ATGTCTGGAGTCAAAGGAGGACATTGTCACCAGAAGACCCAGTGCCAGGATCCCTGCAAAGATCCCTGCCAGAAGCAGAGTGCGTGCCAGGATCCTTGCCAAAAGCA
GAGCGCCTGCCATGACCCATGCCATCCAGTGCAGCATTGTCATGATCAGAGCCCTTGTGGGAAGAAAAAAGATCCATGCAATCCCTGCTGCCCAGATCCCTGCCAGA
GCCAAGGGAAACATCACTGA

>Bbo-aedc18
ATGTCTGGAGTCAAAGGAGGACATTGTCACCAGAAGACCCAGTGCCAGGATCCCTGCAAAGATCCCTGCCAGAAGCAGAACGTGTGCCAGGATCCTTGCCAAAAGCA
GAGCGCCTGCCATGATCCTTGCCAACCAGTGCAGCATTGTCATGATCAGAGCCCTTGTGGGAAGAAAAAAGATCCATGCAATCCCTGCTGCCCAGATCCCTGCCAGA
GCCAAGGGAAACATCACTGA

>Bbo-aedc19
ATGTCTGGAGTCAAAGGAGGACATTGGCACCAGAAGACCCAGTGCCAGGATCCCTGCAAAGATCCCTGCCAGAAGCAGAACGTGTGCCACGATCCTTGCCAAAAGCA
AAGCGCTTGCCATGACCCTTGCCATCCAGTGCAGCATTGTCATGATCAGAGCCCTTGTGGGAAGAAAAAAGATCCATGCAATCCCTGCTGCCCAGATCCCTGCCAGA
GCCAAGGGAAACATCACTGA

>Bbo-aedc20
ATGTCTGGAGTCAAAGGAGGACATTGTCACCAGAAGACCCAGTGCCAGGATCCCTGCAAAGATCCCTGCCAGAAGCAGAGTGCTTGCCATGATCCTTGCCAAAAGCA
GAGCGCCTGCCAGGACCCATGCCATCCAGTACAGCATTGTCATGAGCAGAACCCTTGTGGAAAGAAAAAAGATCCATGCAATCCCTGCTGCCCAGATCCTTGCCAGA
GCCAAGGGAAACATCACTGA

>Bbo-aedc21
ATGTCTGGAGTTAAAGGAGGACATTGTCACCAGAAGACCCAGTGCCAGGATCCCTGCAAGGACCCCTGCCAGAAGCAGAGCGTGTGCCAAGATCCTTGCCAGAAGCA
GAGCGCCTGCCATGACCCATGCAAACCTGTGCAGCATTGCCAGAACCAGAGCACTTGTGTGCAGAAAAAAGACCCTTGCCACCCTGATCCTTGCCAGAGCCAAGCGA
AGCATTGCTAA

>Bbo-aedc22
ATGTCTGGAGTCAAAGGAGGACACTGTCACCAGAAGACCCAGTGCCAGGATCCCTGCCAAAAGCAGAGTGTGTGCCAAGATCCCTGCCAGAAGCAGAGTGTGTGCCA
GGATCCCTGCCAGAAGCAGAGTGTGTGCCAGGATCCCTGCCAGAAGCAGAGTGTGTGCCAGGACCCCTGTAAGCCAGCACAACATTGTCAAGCTCAGAGCGCTGGCG
GCCAGCAAAAAGATCAGTGTCACCAAGATCCATGCCAAAGCCAAGGAAAGCACTGCTGA

>Bbo-aedc23
ATGTCTGGAGTTAAAGGAGGTCCCCACAAGCAACCTGCTCAACAGACCACTGTACAGCCAGCTCCTCAGCCAACTCCTCAGCCAACTCCTCAGCCAACATGCGTTCC
AACACCAGCTCCTCAGCCAACTCCTCAGCCAACATGTCAATCAGTTCCAACACCAGCTCCTCAGCCAACTCCTCAGCCAACATGCGTTCCAACACCAGCTCCTCAGC
CAACTCCTCAGCCAACATGTGTTCCAACACCAGCTCCTCAGCCAACTCCTCAGCCAACATGCGTTCCAACACCAGCTCCTCAGCCAACTCCTCAGACAACATGTGTT
CCAACACCAGCTCCTCAGCCTGCTCCACAGCCAACTCCTCAGTCAACATGTGATTCATCAAAGCCAGCTCCTGTTCCTCATCAGCCCTGCCAGCCAGCTCCCCATCC
TGCCCCTCAGCCAGCTCCACAGCCTCAACTCAACCCTGTGCCTGTTGTGACACCAGCACCTACTCCAGTGCAACCTCCTAAGGGTCAAACCAAGACTGGAGTTAAGA
AATAAAGACGCTTTGTGAACACGTCTGAGGACGGATTGAACAAGCTATAA

>Bbo-aedc24
ATGTCTGGAGTAAAGGGCGGACAGAGTCAATGTCATCAGAATGTCCAGTGTCATGACCCATGCAAGGACCCGTGCCAAAAGCATGACCCATGCAAGGACCTGTGCAA
CGACCCGTGCCAAAAGCATGACCCATGCAAAGACTCATGCCACCCTGTGCAACAGCACTGCCAGGATCATAGCCAGAAATGCTACAAGCAAGATCCCTGTGACCCGT
GCCAGACGCAGACCCACTGTTGTAAAAAGTGA

>Bbo-aedc25
ATGTCAGCCTTTAAGGGAATCTCAGACCTCCAGAACCAAGCTCAACAGAAGACACAAGAAGCTGCAAACCAAGGACAACAAGTGATCCAAGATTGTGTTGATAAAGG
AAAAGAAGTAGGACAACAAGTTATTGACCAAGGATGTGACGCTGCCCAGAAAACATGTGACCAGGTGTCCGGACAAGTTAAAGAAAAGATTCCCGGGGGGTGGCGGA
AATAA

E

>Ame-aedc1
ATGTCAGCCAAGGGAGGATGCAAGGAACCTCAGCAGCCTCAGTGTCAAGAGCCCCAAGTCCAGGGCAAGTGCCAAGAACCCTGTGAACCGGTGCAGCAGTGCCAGGA
CCAGACTCCTCAATGTGTCCCAGAGCCTTGCCCACCTCCATGTCAAGACCAGAAGTCGGTAAAAGGTGTTAAGAAGTGTTAA

>Ame-aedc2
ATGTCAGCTAAGGGAGGATGCAAGGAACCTCAGCAGCCCCAGTGCCAAGAGCCCCAGGTCCAGGGCAAGTGCCAAGAACCCTGTAAACCGGTGCAGCAGTGCCAAGA
CCAGACTCCTCAATGTGTCCCAGAGCCATGCCCACCTCCATGTCAAGACCAGCAGTCAGTAAAAGGAGTTAAGAAGTGTTAA

>Ame-aedc3
ATGTCAGCCAAGGGAGGATGCAAGGAACATCAGCAGCCCCACTGCCAAGAGCCCCAGGTCCAGGGCAAGTGCCAAGAACCCTGTAAACCGGTGCAGCAGTGCCAGGA
CCAGACTCCTCAATGTATCCCAGAGCCATGCCCACCTCCATGTCAAGACCAGCAGTCGGTAAAAGGTGCTAAGAAGTGTTAA

>Ame-aedc4
ATGTCAGCCAAGGGAGGATGCAAGGAACCTCAGCAGCCCCACTGCCAAGAGCCCCAGGTCCAGGGCAAGTGCCAAGAACCCTGTAAACCGGTGCAGCAGTGCCAGGA
CCAGACTCCTCAATGTATCCCAGAGCCATGCCCACCTCCATGTCAAGACCAGCAGTCGGTAAAAGGTGCTAAGAAGTGTTAA

>Ame-aedc5
ATGTCAGCCAAGGGAGGATGCAAGGAACCTCAGCAGCCCCACTGCCAAGAGCCCCAGGTCCAGGGCAAGTGCCAAGAACCCTGTAAACCGGTGCAGCAGTGCCAGGA
CCAGACTCCTCAATGTATCCCAGAGCCATGCCCACCTCCATGTCAAGACCAGCAGTCGGTAAAAGGTGCTAAGAAGTGTTAA

>Ame-aedc6
ATGTCAGCCAAGGGAGGATGCAAGGAACCTCAGCAGCCCCAGTGCCAAGAGACCCAGGTCCAGGCCAAGTGCCAAGAACCCTGTAAACCGGTGCGGCAGTGCCAGGA
CCAGACTGCTCAATGTATCCCAGAGCCTTGCCCACCTCCATGTCAAGACCAGCAGTCAGTAAAAGGAGTTAAGAAGTGTTAA

>Ame-aedc7
ATGTCAGCCAAGGGAGGATGCAAGGAACCTCAGCAGCCCCACTGCCAAGAGCCCCAGGTCCAGGGCAAGTGCCAAGAACCCTGTAAACCGGTGCAGCAGTGCCAGGA
CCAGACTCCTCAATGTATCCCAGAGCCATGCCCACCTCCATGTCAAGACCAGCAGTCGGTAAAAGGAGTTAAGAAGTGTTAA

>Ame-aedc8
ATGTCAGCCAAGGGAGGATGCAAGGAACCTCAGCAGCCCCACTGCCAAGAGCCCCAGGTCCAGGGCAAGTGCCAAGAACCCTGTAAACCGGTGCAGCAGTGCCAGGA
CCAGACTCCTCAATGTATCCCAGAGCCATGCCCACCTCCATGTCAAGACCAGCAGTCGGTAAAAGGTGTTAAGAAGTGTTAA

>Ame-aedc9
ATGTCAGCCAAGGGAGGATGCAAGGAACCTCAGCAGCCCCACTGCCAAGAGCCCCAGGTCCAGGGGAAGTGCCAAGAACCCTGTAAACCGGTGCAGCAGTGCCAGGA
CCAGACTCCTCAATGTATCCCAGAGCCTTGCCCACCTCCATGTCAAGACCAGCAGTCGGTAAAAGGTGTTAAGAAGTGTTAA

>Ame-aedc10
ATGTCAGCCAAGGGAGGATGCAAGGAACCTCAGCAGCCCCACTGCCAAGAGCCCCAGGTCCAGGGCAAGTGCCAAGAACCCTGTGAACCGGTGCAGCAGTGCCAGGA
CCAGACTCCTAAATGTATCCCAGAGCCTTGCCCACCTCCATGTCAAGACCAGCAGTCGGTAAAAGGTGTTAAGAAGTGTTAA

>Ame-aedc11
ATGTCAGCCAAGGGAGGATGCAAGGAACCTCAGCAGCCCCACTGCCAAGAGCCCCAGGTCCAGGGCAAGTGTCAAGAACCCTGTGAACCGGTGCAGCAGTGCCAGGA
TCAGACTCCTCAATGTGTCCCAGAGCCTTGCCCACCTCCATGCCAAGACCAGCAGTCGGTAAAAGGTGTTAAGAAGTGTTAA

>Ame-aedc12
ATGTCTGGATTCAAAGGAGGTGGCTTGGGTGGAGGTGGTGGTGGTGGAAAAGGCTGTGGTGGTGGTGGTTATTATGGCAGTGGTGGGAAAGGCTACAATGGTGGTGG
CTATGGAGGCGGTGGCTATGGTGGCAGTGGTTATTATGGCAGTGGTGGGAAAGGCTACAATGGTGGTGGCTATGGTGGCGGTGGCTATGGTGGCAGTCGTGGGAAAG
GCTATGGTGGTGGTGGTTATGGCCTTGGCGGTTATGGCAGCGGTTCTTTTGGCGGCGGTGGTTTTGGTGGAGGAAACCAGAAGTGGCAAGGCCAAGGTGCATGCCAA

GGAGGAAACACAAATCCATGTGGAGGTTTTCTGGACTGCATACTAGATCCATGTGAATCCTTTTCCCCTTGCTCGGGCCAGGGACAACAATGGGGTGGATGCCAAAA
GTGA

>Ame-aedc13
ATGTCTGCCAAGGGCGGAGGACACCAGAAGACCCAGTGCAATGAACCCTGTCCTCCAGCGCCTTGCCAACAGGTGGACCAGTGCCAGGACCAGCAGCCACAAATATG
CCCAGAACCCTGTAATCCTGATCCATGCCAGGGGGCACAGAAATCGCAAGGAAACCTGCAGGCAGGAGGAGGCGGAGGCATAGGCGGAGGATACGGAGGTAGCGGCG
GCGGATCCTCCAAGAAGTGCTGA

>Ame-aedc14
ATGTCAGCCAAGGGCGGAGGAGGAGGAGGACACCACCACCACCACCACCAGAAGACACAGTGCGAGGAGCCGCAAAACCCCTGCCAAGACCCATGTGCACCAGTCCA
GCAATGCCAAGATAGCAGGAAGGGTTCACAGTCAGTGGATCCTTGCAACCCTTGCCCACCTAATCCCTGCCAGGACCAGCAGGGGGTAGGGCAATATGGTGGAGGAA
GGAAATGA

>Ame-aedc15
ATGTCACTCCCCAAAGGCTTCAAAGGCCTGCCGAAGCAGGCTGAGACTGTGGCCCAAGATGCAGTGGGTCAGGGTGAGCAAGTTGCAAAGGACTGCTTGGAACACGG
AAAAGCAGTTGGACAAGAAGTCATCGATAAAGGATGCAAGGCTGCGAAGGACTCCGGGGAACACGTGGCAGGGCAGATAAAAGGGCAGATCCCCGGATTGAAGAAGA
AGTGA

F

>Pwa-aedc1
ATGTCTGCCAAAGGAGGATACAGGGAAACTCAGAAGACACAGTGCCAAGATACTAAAGTTGTGGTGAAATGCCAGGACCCATGTGCACCAGTGGTGGTGTGCCAGGA
CCCAGTCCCTCAATGTGAACCTCAATGTGAGCCCCAATGTGAGCCAGTCCCATGTGCTCCTGTTTGCCAGGACCAGCACTCAGTAAAAGGAAGCAGGAAGTGTTGA

>Pwa-aedc2
ATGTCTGCCAAGGGAGGACACAGGGAAACTCAGAAGACACAGTGCCAAGATACTAAAGTTGTGGTGAAATGCCAAGAACCATGTGCACCAGTGGTGGTGTGCCAGGA
CCCAGTCCCTCAATGTGAACCTCAATGTGAGCCCCAATGTGAGCCAGCCCCATGTGCTCCTGTTTGCCAGGACCAGCAGTCGGTAAAAGGTGGCAGGAAGTGCTGA

>Pwa-aedc3p
ATGTCTGCCAAGGGAGGACACAGGGAAACTCAGGAGACACAGTGCCAAGATGCTAAAGTTGTGGTGAAATGCCAGGAACCATGTGCACCAGTGGTGGTGCCAGGACC
CAGTCCCTCAATGTGAGCCGCAATGTGA

>Pwa-aedc4
ATGTCTGCTAAGGGCGGAGGACACAGCCAGAAGACCCAGGTCCAGGAGTCTTGCGCTCAAGGCCCATGCAAAACCGTGGACCCATGCCAAGGCCAGCAGTATGGAGG
GAAAGGTCAAGATGCTTGCATGCCTGATCCATGCCAGGGAGCTCAGACTCAACAGCAACAGCAACAGCAGTCAGGCGCCAGCAAAAAATACTGA

>Pwa-aedc5
ATGTCTGCTAAGGGCGGAGGATACAGCCAGAAGACCCAGGTCCAGGAGTCTTGCGCTCAAGGCCCATGCAAAACCGTGGACCCATGCCAAGACCAGCAGTATGGAGG
GAAAGGTCAAGATGCTTGCATGCCTGATCCATGCCAGGGAGCTCAGACTCAACAGCAACAGCAACAGCAGTCAGGCGGCAGCAAAAAATACTGA

>Pwa-aedc6
ATGTCTGCTAAGGGCGGAGGATACAGCCAGAAGACCCAGGTCCAGGAGTCTTGCGCTCAAGGCCCATGCAAAACCGTGGACCCATGCCAAGGCCAGCAGTATGGAGG
GAAAGGTCAAGATGCTTGCATGCCTGATCCATGCCAGGGAGCTCAGACTCAACAGCAACAGCAACAGCAGTCAGGCGCCAGCAAAAAATACTGA

>Pwa-aedc7
ATGTCTGCCAAGGGCGGAGGACACCACCACCAGAAGACGCAGTGCAATGATCCCTGCCAACAGACAAACCCCTGCCAGGACCCATGTGCACCCGTGCAGCAGTGCCA
GGACAACTCACAGAAGTGCGCTCCAGTGGATCCCTGCAATCCATGCATACCTGATCCCTGCCAAGACCAGCAAAGGCCAGGACACTATATCGGAGGCAAGAAGTGA

>Pwa-aedc8
ATGTCTGCCAAGGGCGGAGGACACCACCACCAGAAGACGCAGTGCAATGATCCCTGCCAACAGACAAACCCCTGTCAGGACCCATGTGCACCCGTGCCACAGTGCCA
GGATAACACACAGAAGTGTGCTCCAGTGGATCCCTGCAACCCATGTCCACCTAATCCTTGTCAAGACCAGAATCAAGGGCAATATTATGGAAGCAAGAAGTGTTGA

>Pwa-aedc9
ATGTCTGCCAAGGGCGGAGGACACCACCACCAGAAGCCGCAGTGCAATGATCCCTGCCAACAGACAAACCCCTGTCAGGACCCATGTGCACCCGTGCCACAGTGCCA
GGATAACACACAGAAGTGTGCTCCAGGTGGATCCCTGCAACCCATGTCCGCCTAA

>Pwa-aedc10
ATGTCTGCCAAGGGCGGAGGACACCACCACCAGAAGCCGCAGTGCAATGATCCCTGCCAACAGACAAACCCCTGTCAGGACCCATGTGCACCCGTGCCACAGTGCCA
GGATAACACACAGAAGTGTGCTCCAGTGGATCCCTGCAACCCATGCCCGCCTAATCCTTGCCAAGACCAGAATCGAGGGCAATATTATGGAAGCAAGAAGTGTTGA

>Pwa-aedc11
ATGTCTGCCAAGGGCGGAGGACACCACCACCAGAAGACGCAGTGCAATGATCCCTGCCAACAGACAAACCCCTGTCAGGACCCATGTGCACCCGTGCCACAGTGCCA
GGATAACACACAGAAGTGTGCTCCAGTGGATCCCTGCAACCCATGTCCGCCTGATCCTTGCCAAGACCAGAATCGAGGGCAATATTATGGAAGCAAGAAATGTTGA

>Pwa-aedc12
ATGTCTGCAAAGGGCGGAGGACACCACCAGCAGCAAAAGCAAAAGCCCCAGTGCCCAGAGCCGTGCCCGCCAGAACCCTGCCAAGAACCCTGTGAACCGGTTCAACA
GTGCCAGGACCAGGCAGTTCAGCAGTGCCAGGACCAGGCACCAAAGTGTCCTGAACCAGTTCAACCTTGGAATCCATGCCCACCCGAGCCTTGCCAGGACCAGCAGA
AGCCAGCCCAAAAGGGTGGATGCAAGAAGTGA

>Pwa-aedc13
ATGTCTGCAAAGGGAGGAGGACACCACCAGCAGCACCATCAAAAGCCCCAGTGCCCAGAGCCGTGCCCGCCAGAACCCTGCCAGGAACCATGTAAACCAGTTCAGCA
GTGCCAGGACGAGGTACAGAAGTGTCCTACACCAGTGGAACCTTGTAAACCATCCCCACCGGTGCACTGCCAGGAGCCGCAGAAGCCAGCTCATCAAGGTGGATGCA
AGAAGTGA

>Pwa-aedc14
ATGTCGTACCAAGGACACCACCACCACCAAGGATCCTACTGCCAGGACCCCTGCGACAAGCAGCACCAGCCCTGCCTTGACCCATGCAGACCAGGTCAACGCCATGG
CCATGACCACGGCTACAAGTGTGACAGACCAGATCCATGCTATCCCTCCCCTCCTCATCACCATGCCCATGGTAAATTTACTAACAATTCTGCTGGGTGTAAGAAGT
GA

>Pwa-aedc15
ATGTCGCACCAAGGCCACCACCACCAAGGCCCCCACCACCACCAAGGCAGCCATTGCCAGGACCCGTGCGACAAGCAGCACCAGCCCTGCCATGACCCATGCAAACA
GGGGCACGACCACTGTCATGACCACGGCCACAAGAATGACAAACCAGATCCATGTCATCCTTGCCCTCCTCATCAACACGACCATGGGAAATGCACTCATGGTGGTG
GTGGGTGTAAGAAGTGA

>Pwa-aedc16
ATGTCATTCGCAAAGGGCTGCAAAGGTCTGACAAAACAGGCTCAGAACTTGACGGATGAAGCAGTGGCTCAAGGTGAGCAAGTTGCAAAGGATTGTATGGAACAAGG
GAAGGCAGCAGGACAGGAAGCCATCGACAAAGGGTGCAAGGCTGCTAAGGAGTCAGGCGAGCATGTGGCAGGGCAAGTGAAAGACCAGTTTGCTGGAATGAAGAAAA
AGTGA

G

>Rbi-aedc1
ATGTCTGCAAAGGGTGGTGGTTATCAAAAGCAGCAGTGCCAAGATCCCTGCCAGAAGATACCCAATCCATGCCAAGATCCATGCAAGGATATTCCACAATGTCAGGA
CCAGACATCTAAATGCCAAGATCCATGTGACCCATACCCAAGCCAATGCCAACAGGGTCAAGGAAAAGGATATAAGAAATGTTGA

>Rbi-aedc2
ATGTCTGCAAAGGGTGGAGGTGGTTATCAAAAGCAGCAGTGCCAAGATGATCCATGCCAGAAGATACCGGATCCCTGCCTGAAGACATCGGATCCCTGCCTGAAGAT
ATCGGATCCCTGCCTGAAGATATCGGATCCCTGTCAGAATATACAAGATCCATGCCAAAAGATAGCGGATCCCTGCCAGAAGATAGCGGATCCCTGCCAGAAGTTAG
CGGATCCATGCCTTGACCCATGCAAAACTGTGTCATACTGCCAGGAAAAGACATCCATCTGTCAAGATCCATGTGACCCAGTCCCAACTCAGTGCCAGGAGAAACAC
TGCAAAGGATACACTAAAAATTGTTGA

>Rbi-aedc3
ATGTCTGCAAAGGGTGGTGGTCATCATAAGCAACAGTGCCAAGATCCATGCCAGAAGATATCTGATCCATGTCAAACTTCATGCAAAGATGTGCCACAGTGCCAGGA
CCAGAGACCTAAATGCCCAGATCCATGTGACCCAAGCCCACCCCAGTGCCACCAGGATCAGGGGAAAGGATATACTAAAAAGTGCTAA

>Rbi-aedc4
ATGTCTGCCAAAGGAGGAGGAGGACAGTGGAATCAGAAGACAACATCCCAACAGGACCAAGACAATAACCAAGTGAAAGCCTTTGATCAAACATCCACAATTGGCAA
GGGCGGTGATGGTGGAGGACAGTGGAGTCAGACCACAGGGAGTCAAAGCCAGGGTCAAACAACCAGTGGAGGAAAGTGGAGTCAGACCACAGGGAGTCAAAGCCAGG
GTCAAACAACCACATCTGGCAAGGGCGGTGATTGTGGAGGACAGTGGAGTCAGACCACAGGGAGTCAAAGCACTGGTCAAACAACCAGTGGAGGACAGTGGAGTCAG
ACCACAGGGAGTCAAAGCCAGGGTCAAACAACCACATCAACCACATCAACCACATCTGGCAAAAGCGGTGATTGTGGAGGACAGTGGAGTCAGACCACAGGGAGTCA
AAGCTATGGTCAAACAACCACATCAAGCACATCAACCACATCTGGCAAAGGCGGTGATTGCGGAGGACAGTGGAGTCAGACCACAGGGAGTCAAAGCTATGGTCAAA
CAACCACATCTGGCAAGGGTGGTGATTGTGGAGGTCAGAGCATAGGGGGCTCACAGGTTGGAGGTGATTCATCCCAACAGAAAGGTCAAAGCTATAGTCAGACAAGC
ATAACTTCCAAGGCTGGCAGTGATGGAGGACAGTGCGGTCAGCAGTCCCATGGCTCCCATGATGACCACAAGAAAGGTCACGGTGGAGGTCATCATACAGAGCATCA
CAAACAAGGCCAATCTGGAACAAAGAAGCAGTATTGA

>Rbi-aedc5
ATGTCTGCCAAAGGTGGAGGACAATGGAATCAGAAGTCAAAAGGCCAACAGGATCAAGATGATGACCACTCAAAAGGCCATCATGAACATACATCCTCATCTGGCAA
GGGTGGTGATTGTGGAGGACAGTGGAGTCAGACAACAGGAAGTTCTCAGGTTTCAGTTGACTCAAGCCAACAGAAAAGTCAAGGCCAAACAACCACATCTGGCAAGG
GCGGTGATTGTGGAGGACAGTGGAGTCAGACCACTGGGAGTCAAAGCCAGGGTCAAACAACCACATCTGGCAAGGGCGGTGATTCTGGAGGACAGTGGAGTCAGACC
ACTGGGAGTCAAAGCATTGGTCAAACAACCACATCTGGCAAGGGTGGTGATTCTGGAGGACAGTGGAGTCAGACCACAGGGAGTCAAAGCCAGGGTCAAACAACCAC
ATCTGGCAAGGGCGGTGATTGTGGGGGACAGATGAGTCAGACCACAGGTGGCTCACAGGTTGGAGGCCAAAGCTATAGTCAAACAAGCATAACTTCCAAGGCTGGCA
GTGATGGAGGACAGTGCGGTCAGCAGTCCCACCACTCACATGAAGAACACTCCCATAAGAAAAGCCAAGGTGGAGGTCAATGTGGAGACCAGCAGAAACAAAGCCAA
ACTGGATCAAAGAAGCAATATTGA

>Rbi-aedc6
ATGTCTGCCAAAGGTGGAGGAGGACAGAAATCAAAATGCCAACAGGATCAAGATGATGACCACTCAAAAGGCCATCATGAGCAGAGATCCCCAACTGGCAAGGGCAG
TGACTGTGGAGGACAGTGGAGTCAGACCACAGGGAGTCAAAGCCAGGGTCAGAGATCCCCAACTGGCAAGGGCAGTGACTGTGGAGGACAGTGGAGTCAGAGCACAG
GAGGTCAAGGCCAGAGTCAAACAACCACATCTGGCAAGGGCGGTGATTGTGGAGGGCAGTGGAGTCAAACCACTGGGAGTCAAAGCCATAGTCAAACAACCACATCT
GGCAAGGGTGGTGATTGTGGAGGACAGTTGAGTCAGACCACAGGGAGTCAAAGCCAGGGCCAAACAACCACATCTGGCAAAGGCGGTGATTGTGGAGGACAGTGGAG
TCAGACCACAGGTGGATCGCAGGTTGGCAGTGATTCATGCCAGAAAGGACAAAGCCAAGGCCAGACAAATGTATCTTCCAAGGGTGGTGATGATGGAGGACACTGCG
GTCAGCAGTCACATGAGGACCACAGCCATAAGAAAGGTCAAAGTGGAGGTCAGTGCGGAGACCAGCAGAAACAAAGCCAAAGTGGATCAAAGAAGCTGTATTGA

>Rbi-aedc7
ATGTCTGCCAAAGGTGGAGGACAGTGCAATCAGAATACAAAATGCCAACAGGACCAAGGCAATGACCCATGCCAAGTGAAAGCCTTTGACCAAACAACCACATCTGG
CAAGGGCGGTGATTGTGGAGGACAGTGGAGTCAGACCACTGGGAGTCAAAGCCATAGTCAAACAACCACATCTGGCAAGGGCGGTGATTGTGGAGGACAGAGCACAG
GGAGCCAAAGCTATAGTCAAACAACCACATCTGGCAAGGGCGGTAATTGTGGAGGACAGTGGAGTCAGACCACAGGGGGCTCACAGGCTGGAAGTGATTCATGCCAA
CAGAAAGGTCAAAGCTATGGTCAAACATCCACAAGTGGAAAGGGCAGTGGTTGTGGAGGACAATTGAATCAGAGCACCGGGGGCTCACAGGTTGGAGCTGATTCATG
CCAACAGAAAGGTCAAAACTATAGTCAGACAAACACATCTTCCAAGAGTGGCAATGATGGAGGGCAGTGCGGTCAGCAGTCCCAGTGCGGTCAGCAGTCCCAGGGCT
CACAGGGAGCTGGTAACCAGTGCCAACAGAAAGGTCAAGGTGGAGCTCAGTACACAGAGCAGAAGCAAGGCCAATGTGGATCAAAGAAACAATATTGA

>Rbi-aedc8
ATGTCTGCCAAAGGTGGAGGACAGTGCAATCAGAAGACAACATGCCAACAGGACCAAGGCAATGACCCATGCCAAGTGAAAGCCTTTGATCAAACATCCACAAGTGG
CAAAGTCAGTAATTGTGGAGGACAGTGGAATCAGAATACAGGAGGCTCTCAGGTTGGAGTTGATTCATGCCAACAGAAAGGTCAAAGCTATGGTCAAACATCCACAA
GTGGCAAGGGCAGTAGTTGTGGAGGACAATGGAGTCAGAGCACCGGGGGCTCACAGGTTGGAGTTGATTCATGCCAACAGAAAGGTCAAAGCTATGGCCAAACATCC
ACAAGTGGAAAAGGCAGTGGTTGTGGAGGACAGTCTTATCAGAGCACAGGAGGCTCACAAGTTGGAGTGGATTCATGCCAACAGAAAGGTCAAGGCTATAGTCAAAC
ATCCACAAGTGGCAAAGGCAGTGCTTGTGGAGGACAGTCCTATCAGAGCACAGGGGGCTCACAAGTTGGAGGTGATTCATGCCAACAGAAAGGTCATGGCTATGGTC
AGTCAAGCATTTCATCCAAGGGTGGCAATGATGGAGGGCAGTGCGGTCAGCAGTCCCAGTGTGGTCAGCAGTCTCAGTGCTCACAGGGAGTTGGTAACCAGTGCCAA
CAGAAAGGTCAAAGTGGAAATCAATACACTGACCAGCAGAAACAAGGCCAATGTGGTTCAAAGAAGCAATATTAA

>Rbi-aedc9
ATGTCTGCCAAAGGTGGAAGACAGTGCAATCAGAAGACAACATGCCAACAGGACCAAGGCAATGACTCATGCCAAGTGAAAGCCTTTGATCAAACGTCCACAAGTGG
CAAGGGCAGTGGTTGTGGAGGACAATGGAGTCAGAGCACAGGGGGCTCACAGGTTGTGATTGATTCGTGCCAACAGAAAGATCAAAGCTATGGCCAAACATCCACAA
GTGGCAAAGGCAGTGCTTGTGGAGGACAATGGAATCAGAGCACAGGGGGCTCACAGATTGGAGTTGATTCATGCCAACAGAAAGGTCAAGGCTATAGTCAAACATCC
ACAAGTGGCAAAGGCAGTGCTTGTGGAGGACAATGGAATCAGAGCACAGGGGGCTCACAGATTGGAGTTGATTCATGCCAACAGAAAGGTCAAGGCTATAGTCAAAC
ATCCACAAGTGGCAAAGGCAGTGCTTGTGGAGGACAATGGAATCAGAGCACAGGGGGCTCACAGGTTGGAGTTGATTCATGCCAACAGAAAGGTCAAGGCTATAGTC
AAACATCCACAAGTGGCAAAGGCAGTGGTTGTGGAGGACAGTCGTATCAGAGCACAGGGGGATCACAGGTTGGAGTTGATTCATGCCAACAGAAAGGTCAAGCCTAT
GGTCAGTCAAGCATTTCATCCAAGGGTGGCAATGATGGAGGGCAGTGCGGTCAGCAGTCCCAGTGCGGTCAGCAGTCCCAGTGCTCGCAGGGAGTTGGTAACCAATG
CCAACAGAAAGGTCAAAGTGGAAATCAATACACAGAGCAGCAGAAACAAGGCCAATGTGGTTCAAAGAAGCAATATTAA

>Rbi-aedc10
ATGTCTGCCAAAGGTGGAGGACAGTGCAATCAGATGACAAAATGCCAACAGGACCAAGGCAATGACCCATGCCAAGGAAAAATCTTTGATCAAACATCCACAAGTGG
CAAAGGCAGTGGTTGTGGAGGACAGTCCTATCAGAGCACAGGGGGCTCACAAGTTGATCTGAACTCATGTCAACAGAAAGCTCAAACCTATGGCCAAACATCCACAA
GTGGCAAAGGCAGTGGTTGTGGAGGACAGTCATATCAGAGCACAGGGGGCTCACAGGTTGGAGCTGATTCATGCCAACAGAAAGGTCAAGGCTATGGTCAAACATCC
AACAGTGGCAAAGGCAGTGGTTGTGGAGGACAATTGAATCAGAGCACTGGGGGCTCACAAGTTGGACTGAACTCATGTCAACAGAAAGAAACATCCACAAGTGGCAA
AGGCAGTGGTTGTGGAGGACAATGGAATCAGACCACAGGGGGCTCACAGGTTGGAGTTGATTCATGCCAACAGAAAGGTCAAGGCTATGGTCAAACATCCACAAGTG
GCAAAGGCAATGGTTGTGGAGGACAATTGAATCAGAGCACCGGGGGCTCACAGGTTGGAGTTGCTTCATGCCAACAGAAAGGTCAAAGCTGTGGCCAGACAAACACA
TCTTCCAAGGGCGGCAATGATGGAGGGCAGTGCGGTCAGCAGTCCCAGTGCGGTCAGCAGTCCCAGTGCTCTCAGGGAGTTGGTAACCAATGCCAACAGAAAGGTCA
AGGTGGCGGTCAATACACAGAGCAGCAGAAACAAGGCCAATGTGGATCAAAGAAGCAATATTGA

>Rbi-aedc11
ATGTCTGCCAAAGGTGGAGGACAGTGCAATCAGATGACAAAATGCCAACAGGGCCAAGGCAATGACCCATGCCAAGGAAAAATCTTTGATCAAACATCCACAAGTGG
CAAAGGCAGTGTTTGTGGAGGACAATCGTATCAGAGCACCGGGGGCTCACAAGGTGGTATGAACTCATGTCAACAGAAAGGTCAAACCTATGGCCAAACATCCACAA
GTGGCAAAGGCAGTGGTTGTGGAGGACAGTCATATCAGAGCACAGGGGGCTCACAGGTTGGAGTTGTTTCATGCCAACAGAAAGGTCAAGGCTATGGTCAAACATCC
ACAAGTGGCAAAGGCAGTGGTTGTGGAGGACAATTGAATCAGAGCACCGGGGGCTCACAAGTTGGACTGAACTCATGTCAACAGAAAGGTCAAACCTATGGCCAAAC
ATCCACAAGTGGCAAAGGCAGTGGTTGTGGAGGACAATTGAATCAGAGCACCGGGGGCTCACAGGTTGGAGTTGCTTCATGCCAACAGAAAGGTCAAAGCTGTGGCC

AGACAAACACATCTTCCAAGGGTGGCAATGATGGAGGGCAGTGTGGTCAGCAGTCCCAGTGCGGTCAGCAGTCCCAGTGTGGTCAGCAGTCCCAGTGCTCTCAGGGA
GTTGGTAACCAATGCCAACAGAAAGGTAAAGGTGGAGGTCAATACACAGAGCAACAGAAACAAGGCCAATGTGGATCAAAGAAGCAATATTGA

>Rbi-aedc13
ATGTCTGCCAAAGGTGGAGGAAACTGGAATCAGAAGAAAAAAAGCCAACAGGACCAAGGCAATGACCCACAGCAAGTGAAAGTCTTTGATCAAACATCCACAAGTGG
CAAGAGCAGTGGTTGTGGAGGACAATTGAGTCAGAGCACTGGGGGCTCACAAAGTAGTGGAGATTCATACCAACAGAAAGATCAAAGCTATGGCCAAACATCCACAA
GTGGCAAGAGCAGTGGTTGTGGAGGACAATTGAGTCAGAGCACTGGGAGCTCACAAGGTGGTGGTGATTCATACCAACAGAAAGATCAAAGCTATGGCCAAACATCC
ACTAGTGGCAAGAACAGTGGTTGTGGAGGACAAGTGAGTCAGAGCACTGGGGGCTCACAAGGTGGTCGTGATTCATACCAACAGAAAGATCAAAGCTATGGCCAAAC
ATCCACTAGTGGCAAGAGCAGTGGTTGTGGAGGACAAGTGAGTCAGAGCACTGGGGGCTCACAAGGTGGTCGTGATTCATACCAACAGAAAGATCAAAGCTATAGCG
AAACATCCACAAGTGGCAAGAGCAGTGGTTGTGGAGGACAATTGAGTCAGAGCACTGGGGGCTCACAAGGTGGTGGTGATTCATACCAACAAAAAGATCAAAGCTAT
GGCCAAACATCCACAAGTGGCAAAGGCAGTGATTTTGGAGGACAATGGAAACAGAGCACAGGGGGCTCACAAGGTGGTGGTGATTCATACCAACAAAAAGTTCAAAG
CTCTGGTCAAACATCCACAAGTGGCAAGAGCAGTGGTTGTGGAGGACAATTGAACCAGAGCACTGGGGGCTCACAAAGTAGTGGTGATTCATACCAACAGAAAGATC
AAAGCTATGGCCAAACATCCACGAGTGGCAAGAGCAGTGGTTGTGGAGGACAAATGAGTCAGAGCACTGGGGGCTCACAAGGTGGTCGTGATTCATACCAACAGAAA
GATCAAAGCTATGTCCAAACATCAACGAGTGGCAAGAGCAGTGGTTGTGGTGGACAAGTGAGTCAGAGCACTGGGGGCTCACAAGGTGGTGGTGATTCATACCAACA
GAAAGATCAAAGCTATGTCCAAACATCCACAAGTGGCAAGAGCAGTAGTTGTGGAGGACAATGGAATCAGAGCACTGGGGGCTCACAAGGTGGTGGTGATTCATACC
AACAGAAAGATCAAAGCTATGGCCAAACATCCACAAGTGGCAAAGGCAGTGATTTTGGAGGACAATGGAAACAGAGCACAGGGGGCTCACAAGGTGGTGGTGATTCA
TACCAACAAAAAGTTCAAAGCTCTGGTCAAACATCCACAAGTGGCAAGAGCAGTGGAGGACAATTGAACCAGAGCACTGGGGGCTCACAAAGTAGTGGTGATTCATA
CCAACAGAAAGATCAAAGCTATGGCCAAACATCCACGAGTGGCAAGAGCACTGGTTGTGGAGGACAATGGAACCAGAGCACTGGGGGCTCACAAGGTGGTGGTGATT
CATACCAACAGAAAGATAAAAGCTATGGCCAAACATCCACAAGTGGCAAGAGCAGTGGTTGTGGAGGACAATTGAGTCAGAGCACTGGGGGCTCACAAGGTGGTGGT
GATTCATACCAACAGAAAGATCAAAGCTATGGCCAAACATCAACAAGTGGCAAAGGCAGTGATTTTGGAGGACAATGGAAACAGAGCACAGGGGGCTCGCAAGGTGG
TGGTGATTCATACCAACAGAAAGTTCAAAGCTCTGGTCAAACATCCACGAGTGGCAAGAGCAGTGGTTGTGGAGGACAATTAAGTCAGAGCACTGGGAGCTCACAAG
GTGGTGGTGATTCATACCAACAGAAAGATCAAAGCTATGCCCAAACATCCACAAGTGGCAAGAGCAGTGGTTGTGGAGGACAATTGAACCAGAGCACTGGGGGCTCA
CAAGGTGGTGGTGATTCATACCAACAGAAAGATCAAAGCTATGGCCAAACATCCACAAGTGGCAAAAGCAGTGATTTTGGAGGACAATGGAAACAGAGCACAGGGGG
CTCGCAAGTTGTTGTTGATTCATACCAACAGAAAGTTCAAAGCTCTGGTCAAACATCCACAAGTGGCAAGAGCAGTGGTTGTGGTGGACAATTGAGCCAGAGCACTG
GGAGCTCACAAGGTGGTGGTGATTCATACCAACAGAAAGATCAAAGCTATGGCCGTGGAGGTCAATACACAGACCAACAGAAACAAGGCCAATATGGATCAAAGAAG
CAGTATTGA

>Rbi-aedc14
ATGTCTGGAAAGGGTGGAAGAAGACAGAGACCTAAAAAGAAAGACTGTCAGCAGAATCCAGGCGATAACCCATGCAATGTAGAATTCGAAGTGCTTGATCAGACAGA
GATTTCTGACAAGAATGTTGCTGCTGCTGCTGCTGCTGCTGCTGCTGCTGGGAAATCCAATCAGAAGTCTCAATGCTCACAGAATCCAGTTGATAGCCCATACA
AGAAGTCAAGTCAAACCCAGGATCAGATGGACGTAATTGCTAAGGGAGACAATGATGGAGGACAGTGTAATCAGAAGTCACAGTGCTCACAGCGTGCGGCAGGTGAC
CCATGCAAAAAGTCAAATAAAAAACAGAGTGGTAA

>Rbi-aedc15
ATGGCACATCGTGGTAACTCATGGACAAATAACTACTCCAACCGCTCCAATCATCACTTCAACTCCAACCGCCCCTTCTCCAACTACCAACCTAACCTCTATTTTGG
TCTCACCTCCCCTCAACCCCAACCATACTACCACCACCACCACCACCACCCCAACCGTCCAAGCAACTCATATGTGTATTATTCCAGCCCAGTTGTTGAGAAGCCAA
ATAACCAGACCTGCTGCCAAGACAATTCAAAAGGAAATAAGAAGTAA

>Rbi-aedc16
ATGTTTGGTTGCGGCCGTCAAAGACAATGCTATCAAAACAGACCTCTCTGCCAGGATCTATGTTACAGGCAGGATCCTTGTGCTATTCAAGACCCATGTAATGATCC
ATGCAAACCTACCTACTACAACCAGCGCCAGAGCAACCAATATGTGTATTACCGTAACCCAGGTCAATGCTACCAAAGCTACTGTCAAGACCGTGGCAAAAAATGCA
AGAAGTGA

>Rbi-aedc17
ATGTCTGGTCAAGGTCATTCAGGACACTGCCAGGGACACAAACCCCACTGCCAGGATCTATCTGACAAGCAAGATGAGGGACCATATCCATACAAACCTGACCACCA
CCACCCTCTGCAGCAGCATGACCAGCATGGGCCTCATCCTAAACCAGATCCATACCACCCATCCCATGGTCATGACCATGTGAAAGAATGTAAGAAATGA

>Rbi-aedc18
ATGTCTGCCAAGGGTGGTTATAGTCAATGCCAACAGCAGTCACAGTGCTCAGACCCATGCCAGAACATTGTAATACCAGGTCAATGTCAAGATCCATGTGTACCTGT
GCAGCAGTGGCAGTGCCAGGATCAGACTCCACAGTGTCCTAAGCAAGTTTCCTGCCAGCCATGCCCATCCCAATGCCAAGACCAGGTAAAAGCATACCCTGGTGGAT
GCAAGAAGTGA

>Rbi-aedc19
ATGTCTGCCAAGGGTGGTCACGGTCAGTTCCAACAACAGTCACAGTGCTCACAGCCATGCCAGCAAATACCAGACCAATGTCAAGATCCATGCGCACCTGTACAGCA
GTGCCAGGATCAGACCCCAGAGTGCCTTAAGTCAGTTCCCTGCCAGTCATACCAATCCCAGTGCCAGGAGCAGGTGAAACCCTACCCTGGTGGATGCAAGAAGTAA

>Rbi-aedc20
ATGTCTGCCAAGGGTGGTCACAGTCATTGCCAACAGCAGTCACAGTTCTCAGAGCCATGCCAGCAAATACCAAACCAATGTCAAGATTCATGTGTGCCTATACAGCA
GTGCCAGGATCAGACCCCACAGTGCTCTAAGCCAGATCCCTGTCAGCCATGTCCATCCCAGTGCCAGAACCAGGTGAAACCATTTCCTGGTGGATGCAAGAAGTGA

>Rbi-aedc21
ATGGCTGGTCTCAAGAAAGGTTTTGGTAATTTTAAACACCAGGCTGAGAGTCAAGTGCAGGAAGCAGCATCCCAAGGAGAGCAGATCGTGCAGGACTGCATTGACAA
AGGCAAGCAGTATGGACAGCAAGTTGTAGATGAAGCATGCCACTCTATGCAAGAAGGTTGCGAGCAGGGAGCAGAGAAAGTAAAAAGCCACATACCTGGATTAAAGA
AATGA

H

>Gse-aedc1
ATGTCTGCAAAGGGTGGAGGCTATCAAAAGCAACGATGCCAAGATCCATGCCAGGATCCATGCCAGGATCCATGCAAAGACCCATGTCAGAAGATAGCTGACCCATG
CAAAGACCCGTGCCAGAAGATAGCTGACCCTTGCAAAGACCCATGCCAGCAGATAGCGGATCCATGCCGAGATCCATGTCAGAAGATAGCGGATCCATGCAAAGACC
CATGCCAGAAAATAGCTGACCCATGCAAAGATTCATGCAAGGATGTACCACTGTGCCAGGACCAGATATCTAAGTGCCAAAATCCATGTGACCCATGTGACCCAATC
CAGACCCCATGCCAGCAGGAGAAAGGATATAAGAAGTACTGA

>Gse-aedc2
ATGTCTTCAAAGGGTAGTGGCTATCAAAAGCAACCATGCCAAGATCCTTGCCAGGATCCAAGTCAGCAGATAGGTGACCCAGGCAAAGATTCATTGAAAGGTGTGTC
ACAGTGCCAGGACCAGACATCTAAGTGTCAAAATCCATGTGACCAAGTCTCAAGCCAAAGCCAGCAGGACCAGAACAAAGGATATCAGAAGTGCTGA

>Gse-aedc3
ATGTCTGCAAAGGGTCAATATACTAAGCAGGCATGCCAGGACCCATGCCAGAAGACATCTGTTCCTTGCCAGCAGCCATGTCAGAATGTGTCACAGTGCCAGGATCA
GTCATCTAGTTGCCAAGATCCATGTGCCCCACCACAGAACCCATGCCAGAACCAGGGCCAGGGCAAAGGATATAAGAAGTGCTGA

>Gse-aedc4
ATGTCTTCTTCCAAGGGTGGATGGAAACAAAGCCAACAGGATGAAAGCTATGAAAAAATCACCAGCGGTAGCCATGGCGGTAGCCAGGGTGGTAGCCAGGGCGATGA
CTGTGGAGGAAAATCATGGAATCAGGGCTCAGGAAAGGTTGGCGTTGATGCATCGTACCAGCAAGGCTCTAGCCACACAACTACAACTACAACTTCTTCATCTTCAT
CTTCATCTGGCAAGGGTGGCGACTGTGGAGGAGATCAGTCATGGACCTCACAGGGCTCTCAAGTTACAGTAGGTGATGCAGGCCAAAAGTCAAGTCATCACAGTGGC
CAGACAATCACAACATCAACAACATCTTCAGGTGGAAAGGGCGGTGATTGTGGAGGATCCAGCCAGGTTATAACTGACCAAGAATATAGCCAGACAACCTCTTCTTC
CAAGGGTAGCCAGGATCAAGGACAGTGTGGCCAGAAGTCCCAGCATGAACATCATTCACATCACACACATCATGAGGAATCTCACCAAAAGGGCCACCAAGgtggaa
atcagaaacaatgCACCAGCGGAGGAAAGAAGTGA

>Gse-aedc5
ATGTCTTCCAAGGGAGGTAGTGGCCAGTGGAATCAGCAGGAACAAGGCAACAGCTCATGCCAAAATAAAGGACAAAGCTATGAACAAACATCTACATCTACATCTAC
CCAGGGCAGCGGCTGTGGAGGGCAGTGGAGTCAGAGCTCACAGAGCTCTCAGGGTGGAGCTGGCTCCTGCCAACAGAATACTCAATCCAGTGGAAAAACATCCACAA
CTGTCCAGAGCAGCGGCTGTGGAGGACAGGGGAGTCAGAGCTCACAGAGCTCTCAAAGCTCTCAGAGTGGAGGTGGCTCCTGCCAACAGAATACTCAAATCACTGGA
CAAACAACTACAACTAGTCAGGGCAGTGACTGTGGAGGAAAGTGGAGTCAGAGCTCAAACAGCTCACAGATCTCTCAGGGAGGAGGCAGCTCCTACCAACAGGGAGG
TCAATCCTCTGGACAAACAACTACAACTAGCCAGGGCAGTGACTGTGGAGGAAAGTGGAGTCAGAGCTCAAACAGCTCACAGATCTCTCAGGGAGGAGGTAGCTCCT
ACCAACAGGGAGGTCAATCCTCTGGACAAACAACTACAACTAGCCAGGGCAGTGACTGTGGAGGAAAGTGGAGTCAGAGCTCAAACAGCTCACAGATCTCTCAGGGA
GGAGGCAGCTCCTGCCAACAGGGAGGTCAATCCTCTGGACAAACAACTACAACTAGCCAGGGCAGTGACTGTGGAGGAAAGTGGAGTCAGAGCTCAAACAGCTCACA
GATCTCTCAGGGAGGAGGTAGCTCCTACCAACAGGGAGGTCAATCCTCTGGACAAACAACTACAACTAGCCAGGGCAGTGACTGTGGAGGAAAGTGGAGTCAGAGCT
CAAACAGCTCACAGATCTCTCAGGGAGGAGGCAGCTCCTGCCAACAGGGAGGTCAATCCTCTGGACAAACAACTACAACTAGCCAGGGCAGTGACTGTGGAGGAAAG
TGGAGTCAGAGCTCAAACAGCTCACAGATCTCTCAGGGAGGAGGCAGCTCCTGCCAACAGGGAGGTCAATCCTCTGGACAAATATCCACAAGTGGCCATGGCAGCGG
CTCTGGAGGACAATGGAGTCAGAGCTCACAGAGCTCTCAAAGTACAGTAAGTGGCTCCTGCCAAGACAAAGGACAATCTTATACCCAGACAAGGTCATCTTCCAAGA
GTGGTAATGATGGAGGACAGTGTGGTCAGACCTCACAGGGTGGATCTTATGAGTCATCCCAAAACAAAGGCCAAGGTGGATGTCAAAGCACCGGTGGATCCAAGAAG
TGA

>Gse-aedc6
ATGTCTGCCAAGGGTGGTCGCGGTAGCAGCCAGTGCAATCAGCAGACAACTTACCAACAAAATCAGGGCAACATCTCATGCCAAAACATAAGTCAAGGCTATGATCA
AATATCCACAACTGGCCAGGGCAGTGGCTGTGGAACACAGTGGGGTCAGAGCTCACAGAGCTCTCAGGTTGCAGTTGACTCCTGCCAACAGAAGGGTCAAGTGTCTG
TTCAAATAGGAACTGGCCAGGGCAGTGGCTGTGGAAACCAATGGAGTCAAAACTCACAGAGCTCTCAGGTTGTACAGGTCCAACAAGGTCAAGTTTCTGGACAAACA
TCCACAACCATTCAGGGTAGTGGCTGTGGAGGACAGTGGAATCAGAGCTCACAAGCTGGAGTTAATTCCTGCCAACAGAAAGGTCAAGTTTCTGGACAAACATCTAC
AACCATCCAGGGTAGTGGCTGTGGAGGACAGTGGAATCAGAGCTCACAAGCTGGAGTTAATTCCTGCCAACAGAAAGGTCAAGTTTCTGGACAAATAACCACAACTA
ACCAGAGCAGTGGCTGTGGAGGACAGCAGAGCCAGAGCTCACAGAGCTCTCAGAGTGGAATTGGCTCTTGTCAACAGATAAATCAAGCCTCTACCCAAATTGGAATG
GGCAGTAACTGTGGAGGATCATGGAATCAGCAGACTACACAGAGCTCACAGAATCTAGTCAGTAACTTCTACCAAAATAATGGTCAAGGCGCTAATCAGTCAATGGG
TGGATATGATGGTGGACAGTGTGGTCAGACATCACAGTACTCTCAGAGTGGAGCTTATGACTCATTCCAAAATAAAGGCCAAGGTGGGTGCCAAAACACAGCCCAGC
AGAAGCAATGGATAGGCGGAGCAAAGAAGTGA

>Gse-aedc7
ATGTCTGCCAAGGGTGGTCGCGGTAGTAGCCAGTGCAATCAGCAGACAACTTACCAACAAAATCAAGGCAACATCTCATGCCAAAATAAAAATCAAGACTATGAACA
AACATCCACAACTGGCCAAGGCAATGCATGTGGAGGCCAGTGGGGTCAGAGTTCACAGAGCTCTCAGGTTGTAGTTGTAGTTGACTCCTGCCAACAGAAAGGTCAAG
TGTCTGGACAAATAGGAACAACTGGGCAGGGCAGTGGCTGTGGAAACCAATGGAGTCAAAACTCACAGAGCTCTCAGGTTGTAGTGAATGACTCCTGCCAACAAGGT
CAAGTTTCTGGACAAGCATCCACAACTATCCAGAGTAGTGGCTGTGGAGGACAGTGGAGTCAGAGCTCACAGAGCTCTCAAGTTGGAGTTAATTCCTGCCAACAGAG
AGGTCAAGTTTCTCAACAAATAATCACAACTGGCCAGAGCAGTGGCTGTGGAGGACAGCAGAGCCAGAGCTCTCAGAGCTCTCAGGTTGGAGTTGGCTCCTGTCAAC
AGAAAGATCAAGTCTCTGCTAATATTGGCAAGGGCAGCGACTGTGGAGGATCATGGAACCAGCAAACCACACAGAGCTCACAAAATCTAGTCAATAACTCCTGCCAA
AATAATGGTCAAGGCTCTAATCGTGGAAGTCAATACACCGACCAACAGAAACAATGTACAGGAGCTGCAAAGAAGTGA

>Gse-aedc8
ATGTCTGCCAAGGGTAATAGTGGTTCTGGACAGTGCAGTCAGCAGACATCAAGTCAACAGAATCAAGGCAACAACTCGTGCCAAACTAAAGGACAGAGCTATAATCA
AACATCCACAACTGGCCAGGGCAGTGGCTGTGGAGGGCAAGGGAGTCAGAGCTCACATAGCTCTCAAGTTACAGTAAATAACTCCTGCCAACAGAATAGTCAAGTCT
CTGGAAAAACATCCACAGCAATCCAGGGCAGTGGCTGTGGAGGACAAGGGAGTCAAATCTCACAGAACTCTCAGGGCCAACAGAGAGGCCAAGGATCTGTCCAAACA
TCCACAACTAGCCAGGTTAGTGGCTGTGGAGGACAGGGGAGTCAAAGCTCACAGAGCTCTCAGGTTGTAGCTGGTTCCTGCCAACAGAAAGGTCAAGTGTCTGGACA
AACATCCACATCTAGCCAAAGTAGTAGCTGTGGACAGGGGAGTCAGAGCTCACAGAGTTCTCAGGTTGTAGTGAATAATTCCTGCCAACAGAAAGGTCAAGTGTCTG
AACAAACATCCTGTGGAGGACAGCAGAGCCAGAGCTCCCAGGTTTCAGTAACTAACTCCTGCCAAATTAATAGTCAAGCCTCTGGACAAACATCCACAACTAGCCAG
GGCAGTGGCTGTGGAGGACAGCAGAGCCAGAGCTCACAGAGCTCTCAGGGTGTAGTGAATAACTCCTGCCAACAGAGAAGTCAAGTATCTGGACAATCAAGCTCATC
TTCCAAGGGGGACAATCATGGAGGACAGTGTGGTCAGACATCACAGTACTCACAGGGTGGAGCTTATAACTCATCCCAAAACAAAGGCCAAGGTGAATGTCAAAACA
CAGACCAGCAGAAACAATACACAGGTGGAGTAAAGAAGTGA

>Gse-aedc9
ATGTCTGCCAAGGGTGGTAATGGTGCAGGACTGTGCAATCAGCAGACATCAAGCCAACAGAATCAAGGCAACAACTCATGCCAAAATATAGGACAAGGTTATAATCA
AACATCTACAAGTAGCCAGGACAATGCCTGTGGAGGACAGAGGAGTCAGAGCTCACAGAATTCACAGGTTGTAGTGAATAACTCCTGCCCACAGAAAGGTCAAGTGT
CTGGACAAACATCTACAATTGGTCAGGTCAGTAGCTGTGGAGGACAGGGGAATCAGAGTTCACAGAGCTCTCAGGTTATAGCTGGCTCCTGTCAACAGAAAAGTCAA
GTGATTGGGCAAATATCTACATCTGGTCAGGGCAGTGGTTGTGGAGGACAGCAGAGCCAGAGCTCACAGAGTTCTCAGGTTGTAGTGAATAACTCCTCCCAACAGAA
AGGTCAAATGTCTGGACAATCATCTACATTTGTTCAGAGCAGTGGCTGTGGAGGACAGCAGAACCAGAGCTCACAGATCTCCCAGGTTTCAGTAAATAACTTCTGCC
AAAAGAATAATCAAGTCTCTGGACAAACATCTACATCTGTCCAAGGTAGTGGCTGTGGAGGACAGTGGAGTCAAAGCTCACAGAGCTCTCAAGTTGTAGCCGGCTCC
TGCCAACAGAAAGGTCAACTCTCTGGACAAACATCTATCTCTGTTCAGGGCGGTAACTGTGGAGGACAGTCAAGTCAGAGCTCCCAGAGCTCTCAAGTTGGAGTTGG
CTCCTGCCAACAGAATAGTCAAGTCTCTGGCCGTGGATGTCAAAACACAGACCAGCAGAAGCAATGCACAGGTGGATCAAAGAAGTGA

>Gse-aedc10
ATGTCTGCCAAGGGTGGTAATGGTGCAGGACAGTGCAATCAGCAGACATCAAGCCAACAGAATCAAGGCAACAACTCATGCCAAAATATAGGACAAGGTTATAATCA
AACATCTACAAGTAGCCAGGGCAATGCCTGTGGAGGACAGATGAGTCAGAGCTCACAGAATTCACAGGTTGTAGTGAATAACTCCTGCCAACAGAAAGGTCAAGTGT
CTGGACAAACATCTACATTTGGTCAGGGCAGTAGCTGTGGAGGACAGGGGAATCAGAGTTCACAGAGCTCTCAGGTTATAGCTGGCTCCTGTCAACAGAAAGGTCAA
GTGTCTGGGCAAATATCTACATCTGGTCAGGGCAGTGGTTGTGGAGGACAGCAGAGCCAGAGCTCACAGAGTTCTCAGGTTGTAGTGAATAACTCCTCCCAACAGAA
AGGTCAAATGTCTGGACAATCATCTACATTTGTTCAGAGCAGTGGCTGTGGAGGACAGCAGAACCAGAGCTCACAGATCTCCCAGGTTTCAGTAAATAACTTCTGCC
AAAAGAATAATCAAGTCTCTGGACAAACATCTACATCTGTCCAAGGTAGTGGCTGTGGAGGACAGTGGAGTCAAAGCTCACAGAGCTCTCAAGTTGTAGCCGGCTCC
TGCCAACAGAAAGGTCAACTCTCTGGACAAACATCTACCTCTGTCCAGGGCAGTAGCTGTGGAGGACAGTCAAGTCAGAGCTCCCAGAGCTCTCAAGTTGGAGTTGG
CTCCTGCCAACAGAATAGTCAAGTCTCTGGACGTGGATGTCAAAACACAGACCAGCAGAAGCAATGCACAGGTGGATCAAAGAAGTGA

>Gse-aedc11
ATGTCTGCCAAGGGTGGTAATGGTGCAGGACAGTGCAATCAGCAGTCATCAAGCCAACAGAATCAAGGAAACAACTCATGTCAAAATAAAGGACAAGACTATAATCA
AACATCTTCAACTAGCCAGAACAATGCCTGTGGAGGACAGGGGAGTCAAAGCTCACAGAGCTCTCAGGTCGTAGTGAATAACTCCAGCCAACAGAAAGGCCAAGCAT
CTGGGCAAACATCCACAACTAGCCAGGTTAGTGGCTGTGGAGGACAGGGGAGTCAAAGTTCACAGAGCTCTCAGGTTGTTACTGGTTCCAGCCAACAGAAAGGTCAA
GTGTCTGGACAAACATCTTCATCTGGTCAGGGCAGTGGCTGTGGAGGACAGCAGAGCCAGAGTTCACAGAGTTCACAGGTTGTAGTGAATAACTCCTCCCAACAGAA
AGGTCAAGTGTCTGAACAAACATCCACATTTAGCCAGGGTAGTAGCTGTGGACAGGGGAGTCAGAGCTCACAGAGTTCTCAGGTTGTAGCTGGTTCCAGCCAACAGA
AAGGTCAAGTGTCTGGGCAAACATCATTTGGTCAGGGCAGTAGCTGCGGAGGACAGGAGAGTCAGAGCTCTCAAAGTTCTCAGGTTGTAGTGAATAACTCCTCCCAA
CAGAAAGGTCAAGTGTCTGAACAAACATCTACATCTGTTCAGGGAAGTGGCTGTGGAGGACAGAAGAGCCAGAGCTCACAGAGTTCACAGGTTGTAGTAAATAACTC
CTCCCAACAGAAAGGTCAAGTGTCTGGACAAACATCTACATCTGGTCAAGGCAGTGGCTGTGGAGGACAGCAGAGCCAGAGCGCCCAGAGTTCACAGGTTGTAGTAA
ATAACTCCTCCCAACAGAAAGGTCAAGTGTCTGAACAATCATCTACTTCAGTTCATGGAAGTGGCTGTGGAGGACAGCAGAGTCAGAGCTCACAGAGCTCCCAGGTT
TCAGTAAATAACTCCTGCCAAAAGAATAAAGTCTCTGAACAAACATCTACCTCTGTCCAGGGCAGTAGCTGTGTAGGACAGTCAAGTCAGAGCTCCCAGAGCTCTCA
AGTTGGAGTTGGCTCCAGTCAACAGAATAATCAAGTCTCTGGACGTGGATGTCAAAACCCAGACCAGCAGAAGCAATGCACAGGCGGATCAAAGAAGTAA

>Gse-aedc12p
ATGAACTCCTGCCAACAAAAAGGCCAAACCTCTAGTCAAACAACTTCAACTAGTCAGGGCACTGGCTGTGGAAACCAGTTGAGCCAGAGCTCCCAGAGCTCTCAGGT
TTCAGTCAACTCCTGTCAACAGAGAAATGAAGCCTCTGGTCAAACATCTTCCACTGGCCAGAGCAGTTCCTGTGGAAACCAATAG

>Gse-aedc13
ATGTCTGCCAAGGGTGGTAGTAGAAGTAGCCAGTGCAATCAGCAGACAACACAACAGGAACAAGGCAACACCTCTTGCCAAAACAAAAGTCAAGGCTATGATCACAC
ATCAACAACTGGCAAGGGCAGTGCCTGTGGAGGTCAATCCTCTGGACAAACATCTTCAACTAGCCAGAGCACTGGCTGTGGAAGCCAGTGGACTCAGAGCTCACAGA
GCTCTCAAGGCTCTAGTCAAACATCATCCACTGGCCAGAGCAGTTCCTGTGGAAACCAACGGAGTCAGAGCTCACAGAATCAAGTCTCTGGTCAATCAAGCTCATCT

TCCTCCGGTAGCTATGCTGGAGGACAGTGTGGTCAGACATCCCAGTACTCGCAGAGTGGAGCTAATAATTCATCCCAAAACAAAGGACAAGGTGGATGTCAAAACAC
AAGCCAGCAGAACCAATGTACTGGTGGATCAAAGAAGTGA

>Gse-aedc14
ATGTCTGCCAAGGGTGGTGGTCGTAGTAGCAGCCAGTGGAATCAACAGACAACATACCAACAGGATCAAGGCAACAGCTCATGCCAAAACACAAGTCAAGGCTATGA
TCAAACATCATCAATTGGCCAGACGAGTGACCATGGAAACCAGTTGAGCCAGAGCTCACAGAGCTCTCAGGTTTTAATTGACTCCTCTAAACAGATAAATCAAGGAC
ATGGCCAAATATCTTCAACTGGCCAGAGCAGTGGCAGTGGAAACCAGTGGGGCCAGAGCTCCCAGAGCTCTCAGGTTGCAATTGATTCCTCTCAACAGAGCAGTGGC
TGTGGAAACCAGTGGGGTCAGAGCTCCCAGAGCTCTCAGGTTGCAGTTGACTCCTCTCAACAGAACTATCAAACCTCTAGTCAAATATCTTCAACTGGCCAGAGCAG
TGGCAGTGGAAACCAGTGGGGCCAGAGCTCCCAGAGCTCTCAGGTTGCAATTGATTCCTCTCAACAGAGCAGTGGCTGTGGAAACCAGTGGGGCCAGAGCTCCCAGA
GCTCTCAGGTTGCAGTTGACTCCTCTCAACAGAACTATCAAACCTCTAGTCAAATATCTTCAACTGGCCAGAACAGTGGCAGTGGAAACCAGTGGGGCCAGAGCTCC
CAGAGCTCTCAGGTTGCAATTGATTCCTCTCAACAGAGCAGTGGCTGTGGAAACCAGTGGGGCCAGAGCTCCCAGAGCTCTCAGGTTGCAATTGATTCCTCTCAACA
GAACTATCAAACCTCTAGTCAAATATCTTCAACTGGCCAGAACAGTGGCAGTGGAAACCAGTGGGGCCAGAGCTCCCAGAGCTCTCAGGTTGCAATTGATTCCTCTC
AACAGAGCAGTGGCTGTGGAAACCAGTGGGGCCAGAGCTCCCAGAGCTCTCAGGTTGCAATTGATTCCTCTCAACAGAACTATCAAACCTCTAGTCAAATATCTTCA
ACTGGCCAGAACAGTGGCAGTGGAAACCAGTGGGGCCAGAGCTCCCAGAGCTCTCAGGTTGCAATTGATTCCTCTCAACAGAACTATCAAACCTCTAGTCAAATATC
ATCAACTGGCCAGGGCACAGGCTGTGGAAACCAAGGAGGTTATAGTTCGCAGATTCAAGTCTCTGATCAAACCTTCATTAGTGGTCAGGGAGGTGCTTCTGGAAACC
AGTGGAATCAGAGCTCACAGAGTTCTCAGATTGCAGTTGACAACTTAATGCAACAGAAAGGTCCAGTCTATGGTCAAGCCTTCATTACTGGCCAGGGAGGTGCTTCT
GGAAACCAGTGGAATCAGAGTTCACAGAGTTCTCAGAGTGCAGTTAACAACTTAATGCAGCAGAAAGGTCCAGTCTATGGTCAAACCTTTACTAGTGGCCAGGGAGG
TGCTTCTGGGAACCAGTGGAATCAGAGCTCACAGAATTCCCAAATTTCAGTTGACAGCTCAATGCAACAGAAAGGTTCAGGATTTGGTCAATCAAGCTCGTTTTCCC
AAGGTGGCTATGATGGAGGACAGTATGGTCAGACATCACAGTACTCGCAGAGTGGAGCCCAAAACTTTGGCCAAGGTGGATATCAAAACACAAGCCAGCAGAACCAA
TTCATTGGTGGACAAAAGAAGTGA

>Gse-aedc15
ATGGCTACCAAGGGTCGTAGAAGACAAACAGCTCCAAAGGCAGAGTGTCATCAGGAACCAGGTGGTGCCCCATGCACCATAGAAACATCTGTGTCTGATCATGATGA
CATATCTGACTACAATGCTGAGGTTAGTTTGATGAGCATCCATAATTCAACAATCTCATTTAAACATGATGATGTTCAAAATGAACCTGATGATGCTCTCCGAAACT
TAGGTCACACAAGGGATTGTGGTAATAATGGAAGACAGTATGAGTATAACCAGCAATCACAGGACTCACAGGTTTCAGTAGAGAACCAATGCAGACAGTCAGGCCAT
AGCCAGAACCGTGGTAATAATGGAAGACAGTATGAGTATAACCAGCAATCACAGGACTCACAGGTTTCAGTAGAGAACCAATGCAGACAGTCAGGCCATAGCCAGAA
CCAGACAGATGTAATCTACAAAGGTGGCAGTGATGAGGGGCAGTGTTATATGCAGTCACAGTGCTCAAAGGCTCCAATAGAGAACACATGCACACAGCCAAGCCAAA
GGCAAAACCTTTCAACAAGTGGCTCTGTATCAACGTGCCAGAACCAAAGTTCCTCTTCACCCGCCAGATCAGGCGTACAGTATGGAGATGTCCAGAAACAGTGCCCA
GGTACAAGAAAGAACTGA

>Gse-aedc16
ATGTCCTTTTGGGGCCGTCAAAGTCAATGCTGTCCTCCCAGCCCAGACTGCTGTCCCACACAGGATCCTTGTGCAAGACAAAACCCATGTTATAATCCCTGCAAGCC
TGCCCAATGCTACCCACAGAATAACCAGTATACATATTACCGTGGCATAGGTTCATGGTCTCAAAACAAAGGTTGCCAAAGCCCTGGAAAATATTGCAAGAAGTGA

>Gse-aedc17
ATGTCTGACCATGGCCATCCAGGACACTGCCAGGAACACAAACCCCATGATCACCACCACAAGCATGACATGGGAAAACATTCGTGCAAACCTGACCACCATCACCC
TGAGCATGAGCATCACCATGACCACCATGCACATCACCCTAACGCAGCTCCGTGTGATCCACACCATCCATCCCAAAGCCACAGCCACGTGAAAGAACATAAGAAAT
GA

>Gse-aedc18
ATGGCTAGTCTCAAGAAAGGATTAGGTAATATTCAACAACAGGCTAAGAGTGAGGTGCAGGATGCAGCATGCCAAGGTGAGCAGGTCGTGCATGACTGTATTGATAA
GGGGAAGCAGGTTGGCCAACAAGTTCTAGATCAAGCATCCCATGCTGTCCAGGAGGCCTGTGAGGAAGTAACAGGCCAAGTGAAAAGCCATATGCCATCTGTGCATA
AAAAATGA

Figure S9. Coding sequences of AEDC genes. Species: (**A**) Western clawed frog (*Xenopus tropicalis,* Xtr), (**B**) African clawed frog (*Xenopus laevis,* Xla), (**C**) common toad (*Bufo bufo*, Bbu), (**D**) fire-bellied toad (*Bombina bombina*, Bbo), (**E**) axolotl (*Ambystoma mexicanum,* Ame), (**F**) iberian ribbed newt (*Pleurodeles waltl*, Pwa), (**G**) two-lined caecilian (*Rhinatrema bivittatum,* Rbi), (**H**) Gaboon caecilian (*Geotrypetes seraphini*, Gse).
